# Gene Expression Changes under Cyclic Mechanical Stretching in Rat Retinal Glial (Müller) Cells

**DOI:** 10.1371/journal.pone.0063467

**Published:** 2013-05-27

**Authors:** Xin Wang, Jiawen Fan, Meng Zhang, Zhongcui Sun, Gezhi Xu

**Affiliations:** 1 Department of Ophthalmology and Vision Sciences, Eye and ENT Hospital, Shanghai Medical College, Fudan University, Shanghai, China; 2 Shanghai Key Laboratory of Visual Impairment and Restoration, Fudan University, Shanghai, China; Institut de la Vision, France

## Abstract

**Objective:**

The retina is subjected to tractional forces in various conditions. As the predominant glial element in the retina, Müller cells are active players in all forms of retinal injury and disease. In this study, we aim to identify patterns of gene expression changes induced by cyclic mechanical stretching in Müller cells.

**Methods:**

Rat Müller cells were seeded onto flexible bottom culture plates and subjected to a cyclic stretching regimen of 15% equibiaxial stretching for 1 and 24 h. RNA was extracted and amplified, labeled, and hybridized to rat genome microarrays. The expression profiles were analyzed using GeneSpring software, and gene ontology analysis and the Kyoto Encyclopedia of Genes and Genomes (KEGG) were used to select, annotate, and visualize genes by function and pathway. The selected genes of interest were further validated by Quantitative Real-time PCR (qPCR).

**Results:**

Microarray data analysis showed that at 1 and 24 h, the expression of 532 and 991 genes in the Müller cells significantly (t-test, p<0.05) differed between the mechanically stretched and unstretched groups. Of these genes, 56 genes at 1 h and 62 genes at 24 h showed more than a twofold change in expression. Several genes related to response to stimulus (e.g., Egr2, IL6), cell proliferation (e.g., Areg, Atf3), tissue remodeling (e.g., PVR, Loxl2), and vasculogenesis (e.g., Epha2, Nrn1) were selected and validated by qPCR. KEGG pathway analysis showed significant changes in MAPK signaling at both time points.

**Conclusions:**

Cyclic mechanical strain induces extensive changes in the gene expression in Müller cells through multiple molecular pathways. These results indicate the complex mechanoresponsive nature of Müller cells, and they provide novel insights into possible molecular mechanisms that would account for many retinal diseases in which the retina is often subjected to mechanical forces, such as pathological myopia and proliferative vitreoretinopathy.

## Introduction

The retina, which is responsible for encoding and processing visual stimulus, is subjected to tractional forces in various conditions. For instance, pathological myopia, which is one of the leading causes of blindness, is characterized by excessive and progressive elongation of the eyeball with concomitant degenerative changes in the posterior segment of the eye [1], [2]. During the progressive distension of the posterior pole, the retina is overstretched, as a result of which retinal remodeling occurs. Moreover, mechanical stretching of the retina can also be observed over the course of posterior vitreous detachment [3], proliferative vitreoretinopathy, and so on. However, the cellular and molecular effects of mechanical stretching of the retina are relatively unexplored, and therefore, further research is required in this regard.

As the predominant glial element in the sensory retina, Müller cells are responsible for the homeostatic and metabolic support of retinal neurons, and they are active players in virtually all forms of retinal injury and disease [4], [5], [6]. Moreover, structurally, Müller cells span the entire retinal thickness, extending from the inner to the outer limiting membranes, with cell bodies located in the inner nuclear layer and lateral processes expanding into the plexiform layers of the tissue [6]. Because of this unique morphology, Müller cells can sense even minute changes in the retinal structure because of the mechanical stretching of their long processes or side branches. Thus, it is reasonable to infer that Müller cells also participate in ocular diseases where the retina is overstretched. In fact, a recent study confirmed that they were sensitive and responsive to tissue stretching [7]. However, the molecular effects of mechanical stretching on Müller cells remain unclear.

In this study, we aim to investigate the genome regulation of Müller cells under mechanical stretching in detail; this may provide clues to understanding the molecular mechanisms that would account for many retinal diseases in which the retina is often subjected to mechanical forces.

## Results

### Identification of Differentially Expressed Genes

Differential gene expression analysis showed that at 1 and 24 h, the expression of 532 and 991 genes significantly (t-test, p<0.05) changed between the mechanically stretched and the control groups (Tables S1 and S2). Of these, at 1 h, 56 genes, with 48 genes up and 8 genes down, showed more than a twofold change in expression (Table 1). At 24 h, 62 genes, with 16 genes up and 46 genes down, showed more than a twofold change in expression (Table 2). Subsequent analysis focused on these genes that showed a more than twofold change in expression (which was considered significant).

**Table 1:** Up- and downregulated Genes (p<0.05, more than twofold change) in Müller cells after stretching for 1 h.

Accession#	Gene	Gene Title	Fold^a^
NM_017352	Nr4a3	Nuclear receptor subfamily 4, group A, member 3	15.14
NM_133578	Dusp5	Dual specificity phosphatase 5	8.16
NM_001008826	LOC360231	MHC class I RT1.O type 149 processed pseudogene	7.29
NM_012912	Atf3#	Activating transcription factor 3	5.72
NM_017123	Areg#	Amphiregulin	5.47
NM_138526	Ccrn4l	CCR4 carbon catabolite repression 4-like	5.43
NM_017259	Btg2	BTG family, member 2	5.24
NM_001108510	Dusp8	Dual specificity phosphatase 8	5.10
NM_024388	Nr4a1	Nuclear receptor subfamily 4, group A, member 1	4.82
XM_001056859	Sprr1al	Small proline-rich protein 1A-like	4.62
NM_017259	Btg2	BTG family, member 2	4.43
NM_021689	Ereg	Epiregulin	4.05
NM_012945	Hbegf#	Heparin-binding EGF-like growth factor	3.76
NM_001014071	Errfi1	ERBB receptor feedback inhibitor 1	3.66
NM_031707	Homer1	Homer homolog 1 (Drosophila)	3.60
NM_053883	Dusp6	Dual specificity phosphatase 6	3.53
NM_012589	IL6#	Interleukin 6	3.20
NM_012603	Myc#	Myelocytomatosis oncogene	3.11
NM_053769	Dusp1#	Dual specificity phosphatase 1	2.97
NM_001079890	Gprc5a	G protein-coupled receptor, family C, group 5, member A	3.06
NM_153724	Rcan1	Regulator of calcineurin 1	2.99
NM_017180	Phlda1	Pleckstrin homology-like domain, family A, member 1	2.97
NM_053633	Egr2#	Early growth response 2	2.92
NM_019328	Nr4a2	Nuclear receptor subfamily 4, group A, member 2	2.78
NM_012953	Fosl1	Fos-like antigen 1	2.75
NM_053713	Klf4	Kruppel-like factor 4 (gut)	2.71
NM_013058	Id3	Inhibitor of DNA binding 3	2.65
NM_053382	Tnfaip6	Tumor necrosis factor alpha induced protein 6	2.56
NM_001047858	Srxn1	Sulfiredoxin 1 homolog (S. cerevisiae)	2.56
NM_021835	Jun#	Jun oncogene	2.35
NM_031971	Hspa1	Heat shock 70kD protein 1	2.51
NM_013153	Has2#	Hyaluronan synthase 2	2.49
NM_012620	Serpine1	Serine (or cysteine) peptidase inhibitor, clade E, member 1	2.48
NM_001012046	Spry2	Sprouty homolog 2	2.43
NM_031971	Hspa1a	Heat shock 70kD protein 1A	2.43
NM_024381	Gk	Glycerol kinase	2.38
NM_017232	Ptgs2#	Prostaglandin-endoperoxide synthase 2	2.37
XM_002728821	LOC100360845	Hypothetical protein LOC100360845	2.16
NM_001110860	Crem	cAMP responsive element modulator	2.15
NM_001014094	Plscr2	Phospholipid scramblase 2	2.14
NM_019242	Ifrd1	Interferon-related developmental regulator 1	2.12
NM_001169116	RGD1306119	Similar to transcriptional regulating protein 132	2.11
NM_012620	Serpine1	Serine (or cysteine) peptidase inhibitor, clade E, member 1	2.09
NM_023985	Trib1	Tribbles homolog 1	2.05
NM_001047858	Srxn1	Sulfiredoxin 1 homolog	2.05
NM_017076	PVR#	Poliovirus receptor	2.04
NM_001108977	Epha2#	Eph receptor A2	2.02
NM_001106779	Nedd1	Neural precursor cell expressed, developmentally down-regulated 1	0.48
XM_001077448	Dact1	Dapper, antagonist of beta-catenin, homolog 1	0.48
NM_001107250	Znf503	Zinc finger protein 503	0.48
NM_080906	Ddit4	DNA-damage-inducible transcript 4	0.46
NM_013148	Htr5a	5-Hydroxytryptamine (serotonin) receptor 5A	0.45
XM_001072241	Maml2	Mastermind like 2	0.40
NM_001008767	Txnip	Thioredoxin interacting protein	0.34
NM_001108654	Tox	Thymocyte selection-associated high mobility group box	0.31

a Fold change greater than 1.0 represents increases, while less than 1.0 indicates decreases in stretching versus control group.

# Indicates gene expression result obtained from microarray analysis was further verified using qPCR.

**Table 2:** Up- and downregulated Genes (p<0.05, more than twofold change) in Müller cells after stretching for 24 h.

Accession#	Gene	Gene Title	Fold^a^
NM_001106637	Gem	GTP binding protein	2.70
XM_001063122	LOC685277	Similar to liver-specific bHLH-Zip transcription factor	2.66
NM_053346	Nrn1#	neuritin 1	2.60
NM_001191721	Rps6ka6	Ribosomal protein S6 kinase polypeptide 6	2.60
NM_001109344	RGD1562846	Similar to Docking protein 5 (Downstream of tyrosine kinase 5)	2.55
NM_019176	Stmn4	Stathmin-like 4	2.40
NM_053802	Tgfbi	Transforming growth factor, beta induced	2.33
NM_001014193	RGD1359529	Similar to chromosome 1 open reading frame 63	2.28
NM_001107464	Dact2	Dapper, antagonist of beta-catenin, homolog 2	2.28
NM_001167840	IL1rap	Interleukin 1 receptor accessory protein	2.27
NM_001134986	Rnf180	Ring finger protein 180	2.25
NM_031522	Neu1	Sialidase 1 (lysosomal sialidase)	2.20
NM_130812	Cdkn2b	Cyclin-dependent kinase inhibitor 2B (p15, inhibits CDK4)	2.19
NM_001106909	RGD1309095	Similar to hypothetical protein BC015148	2.15
NM_017094	Ghr	Growth hormone receptor	2.06
NM_001106047	Loxl2#	Lysyl oxidase-like 2	2.05
NM_001106550	Nkain4	Na+/K+ transporting ATPase interacting 4	0.50
NM_001106134	Ska1	Spindle and kinetochore associated complex subunit 1	0.50
NM_019189	Hapln1	Hyaluronan and proteoglycan link protein 1	0.49
NM_175578	Rcan2	Regulator of calcineurin 2	0.49
NM_001107956	Car9	Carbonic anhydrase 9	0.49
NM_019212	Acta1	Actin, alpha 1	0.49
NM_022183	Top2a	Topoisomerase (DNA) II alpha	0.49
NM_138502	Mgll	Monoglyceride lipase	0.48
NM_001106623	RGD1311164	Similar to DNA segment, Chr 6	0.48
NM_001039549	Ugt1a	UDP glucuronosyltransferase 1 family, polypeptide A	0.48
NM_138905	Ppap2b	Phosphatidic acid phosphatase type 2B	0.48
NM_031582	Aoc3	Amine oxidase, copper containing 3 (vascular adhesion protein 1)	0.48
NM_012545	Ddc	Dopa decarboxylase (aromatic L-amino acid decarboxylase)	0.47
NM_001106465	Ntng1	Netrin G1	0.47
NM_012715	Adm#	Adrenomedullin	0.46
NM_001013222	Rnd1	Rho family GTPase 1	0.46
NM_001106306	Cpxm2	Carboxypeptidase X (M14 family), member 2	0.45
NM_001007648	Cdca3	Cell division cycle associated 3	0.45
NM_181635	Kif15	Kinesin family member 15	0.45
NM_012550	Ednra	Endothelin receptor type A	0.44
NM_001108009	Rasgrp3	RAS guanyl releasing protein 3 (calcium and DAG-regulated)	0.44
NM_022183	Top2a	Topoisomerase (DNA) II alpha	0.44
NM_024388	Nr4a1	Nuclear receptor subfamily 4, group A, member 1	0.44
NM_172033	Plekhb1	Pleckstrin homology domain containing, family B (evectins) member 1	0.43
NM_053848	Opcml	Opioid binding protein/cell adhesion molecule-like	0.43
NM_138905	Ppap2b	Phosphatidic acid phosphatase type 2B	0.43
XM_001078892	Gbp4	Guanylate binding protein 4	0.42
NM_181087	Cyp26b1	Cytochrome P450, family 26, subfamily b, polypeptide 1	0.42
XM_001069190	RGD1563437	Similar to KIAA1217	0.41
NM_001011893	4-Sep	Septin 4	0.41
NM_031834	Sult1a1	Sulfotransferase family, cytosolic, 1A, phenol-preferring, member 1	0.41
XM_001059692	RGD1307396	Similar to RIKEN cDNA 6330406I15	0.40
NM_053633	Egr2#	Early growth response 2	0.40
XM_001056542	LOC679475	Hypothetical protein LOC679475	0.39
NM_017226	Padi2	Peptidyl arginine deiminase, type II	0.39
NM_153737	Sostdc1	Sclerostin domain containing 1	0.39
NM_013122	Igfbp2	Insulin-like growth factor binding protein 2	0.36
NM_001107221	C1qtnf7	C1q and tumor necrosis factor related protein 7	0.34
NM_022707	Pln	Phospholamban	0.34
NM_022197	Fos#	FBJ osteosarcoma oncogene	0.33
NM_019292	Car3	Carbonic anhydrase 3	0.32
NM_022257	Masp1	Mannan-binding lectin serine peptidase 1	0.31
NM_031739	Kcnd3	Potassium voltage-gated channel, Shal-related subfamily, member 3	0.31
NM_012598	Lpl	Lipoprotein lipase	0.30
NM_021576	Nt5e	5′-Nucleotidase, ecto	0.22
NM_001135855	Scara5	Scavenger receptor class A, member 5 (putative)	0.21

a Fold change greater than 1.0 represents increases, while less than 1.0 indicates decreases in stretching versus control group.

# Indicates gene expression result obtained from microarray analysis was further verified using qPCR.

**Figure 1:**
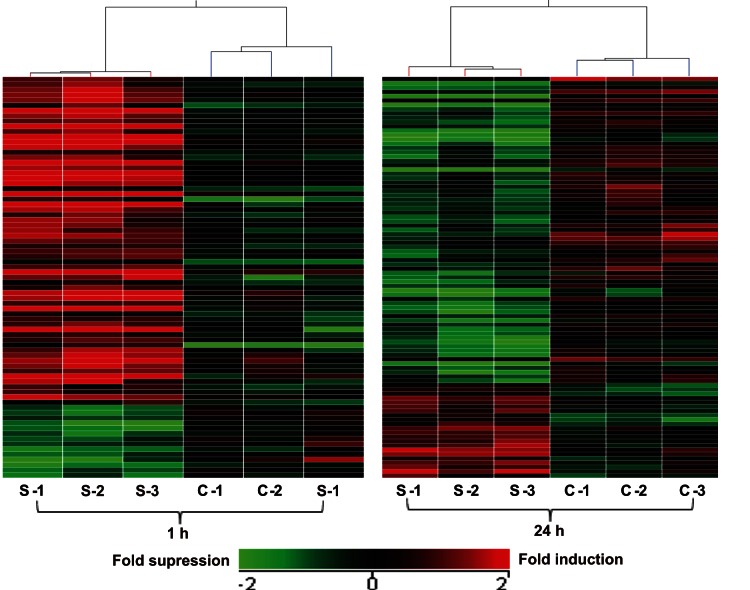
Hierarchical cluster analysis of differentially expressed genes (p<0.05, more than twofold change) at 1 and 24 h. Each row represents a probe and each column represents one sample. The values represent the fold changes compared with the corresponding control. Positive and negative fold changes are shown in red and green, respectively, as shown in the color bar. S, stretching group; C, control group.

**Figure 2:**
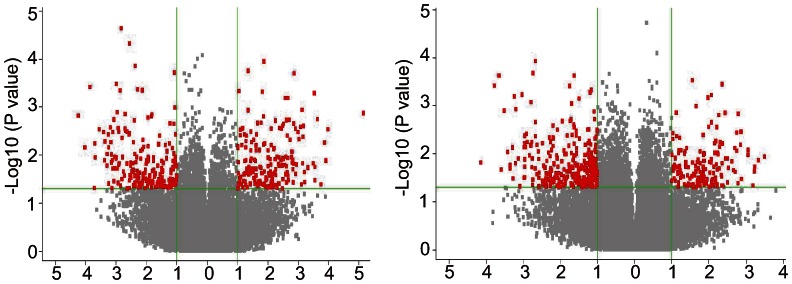
Volcano plot of stretching vs. control at 1 and 24 h. Each point represents a gene plotted as a function of fold change (Log2(fold change), x-axis) and statistical significance (-Log10(p-value), y-axis). The vertical dotted line represents twofold changes. The horizontal dotted line represents p = 0.05. The red dots represent selected differentially expressed genes with p<0.05 and more than twofold change.

To visualize gene expression profiling at each time point, a hierarchical clustering analysis was carried out (Figure 1). The mechanically stretched and control cell cultures clustered independently in two separate primary branches of the dendrogram at both 1 and 24 h, indicating that Müller cells were responsive to stretching. More genes were up regulated at 1 h than at 24 h. To better demonstrate the process of identifying significant genes, volcano plots were also presented based on the microarray result (Figure 2). The red dots represent selected differentially expressed genes with p<0.05 and more than twofold change, most of which are listed in Tables 1 and 2.

### Functional Gene Categories Regulated by Mechanical Stretching

As summarized in Table S3, after mechanical stretching for 1 h, 6 molecular functions, 5 cellular components, and 99 biological processes were significantly upregulated. These biological processes included cellular response to stimulus, multicellular organismal development, anatomical structure formation involved in morphogenesis, cell development, cell death, biosynthetic process, cell motion, cell proliferation, tissue remodeling, positive regulation of anti-apoptosis, positive regulation of metabolic process, and positive regulation of biological process. In contrast, only 1 cellular component, organelle envelope lumen, was significantly downregulated.

However, after mechanical stretching for 24 h, the number of significantly upregulated gene categories was much less than that of downregulated ones. The former only involved 1 molecular function, phosphate binding, and 1 biological process, multicellular organismal metabolic process. In contrast, the latter included 2 molecular functions, lyase activity and pattern binding, and 2 cellular components, extracellular region part and extracellular space. Moreover, 36 biological processes were also downregulated, including response to stimulus, multicellular organismal development, cell development, cell division, and anatomical structure formation involved in morphogenesis (Table S4).

### Pathways Associated with Differentially Expressed Genes

KEGG pathway analysis was used to further analyze differentially expressed genes (p<0.05, more than twofold change) after mechanical stretching of Müller cells at both 1 and 24 h. The enrichment analysis revealed that 16 pathways were significant in differentially expressed genes at 1 h, and 20 pathways were significant at 24 h (p<0.05). At 1 h, the top five pathways were ErbB signaling pathway, MAPK pathway, Jak-STAT signaling pathway, pathways in cancer, and Wnt signaling pathway (Table 3). At 24 h, the top five pathways were MAPK signaling pathway and 4 metabolism pathways (Table 4).

**Table 3:** Significantly changed pathways in Müller cells after stretching for 1 h.

Pathway Name	Genes (n)	P-value
ErbB signaling pathway	5	0.0000
MAPK signaling pathway	9	0.0000
Jak-STAT signaling pathway	3	0.0013
Pathways in cancer	4	0.0013
Wnt signaling pathway	3	0.0013
Prion diseases	2	0.0014
Leishmania infection	2	0.0042
Colorectal cancer	2	0.0051
TGF-beta signaling pathway	2	0.0067
Small cell lung cancer	2	0.0083
Toll-like receptor signaling pathway	2	0.0088
GnRH signaling pathway	2	0.009
Antigen processing and presentation	2	0.0104
Spliceosome	2	0.0154
Thyroid cancer	1	0.0441
Endocytosis	2	0.0458

**Table 4:** Significantly changed pathways in Müller cells after stretching for 24 h.

Pathway Name	Genes (n)	P-value
Glycerolipid metabolism	3	0.0001
Phenylalanine metabolism	2	0.0004
Nitrogen metabolism	2	0.0008
MAPK signaling pathway	4	0.0013
Tyrosine metabolism	2	0.0016
Sphingolipid metabolism	2	0.0026
Retinol metabolism	2	0.0066
B cell receptor signaling pathway	2	0.0089
Metabolic pathways	6	0.0171
Sulfur metabolism	1	0.0203
Vascular smooth muscle contraction	2	0.0209
Axon guidance	2	0.0227
Other glycan degradation	1	0.0286
beta-Alanine metabolism	1	0.0385
Nicotinate and nicotinamide metabolism	1	0.0401
Pentose and glucuronate interconversions	1	0.0401
Histidine metabolism	1	0.0418
Calcium signaling pathway	2	0.0431
Ascorbate and aldarate metabolism	1	0.0434
Homologous recombination	1	0.0450

**Figure 3:**
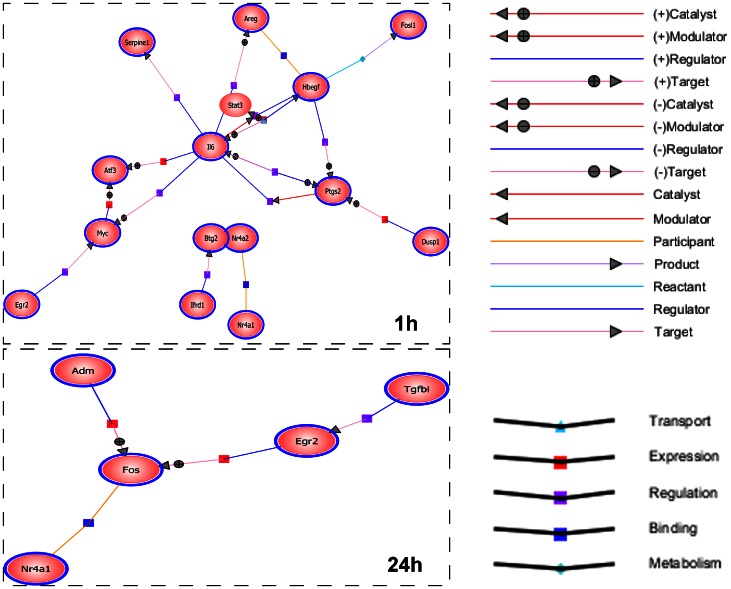
Interaction networks of gene products induced by mechanical stretching at 1 and 24 h. This diagram, prepared using Gene Spring Software, illustrates the known direct interactions between the proteins encoded by 56 genes at 1 h and 62 genes at 24 h (p<0.05, more than twofold change). IL 6, Hbegf, Ptgs2, and Myc at 1 h and Fos at 24 h show strong interaction centers. Ovals surrounded by blue lines represent proteins included in the above list.

The Protein-Protein Interactions analysis was further performed to identify the direct interaction of these genes products using GeneSpring GX 11.5 (Figure 3). The diagram illustrates particularly strong interaction centers for IL 6, Hbegf, Ptgs2, and Myc at 1 h and for Fos at 24 h. In addition, all genes in the 1 h interaction network were upregulated whereas those in the 24 h network were downregulated.

### Validation of Selected Differentially Expressed Genes by qPCR

Based on a combination of statistical analysis of microarray data and potential biological importance of the genes of interest, 16 genes were chosen for qPCR confirmation (Table 5). These 16 genes are related to response to stimulus (Areg, Egr2, Jun, PVR, Myc, Dusp1, IL6, Ptgs2, Adm, and Fos), cell proliferation (Areg, Jun, Myc, IL6, Ptgs2, Hbegf, and Atf3), tissue remodeling (Areg, PVR, IL6, Loxl2, and Has2), and vasculogenesis (Epha2, Nrn1). Though genes varied slightly in the changes they showed between microarray and qPCR results, there was a clear consistency between the two techniques (Tables 6 and 7), validating the results obtained from microarray analysis.

**Table 5:** Primers Used for qPCR.

Gene Symbol	Genebank	Primer Sequence	Product Size (bp)	
Areg	NM_017123	F: CGCAGCTATTGGCATCATTA R: TTCTGCTTCTTCATATTCCCTGAA		71
Egr2	NM_053633	F: TCCTCTGTGCCTTGTGTGATG R: ACCCAGGGAGTGATTTTTTTTTC		89
Jun	NM_021835	F: GATTGCTTCTGTAGTGCTCCGTAA R: ATCCGCAATCTAGCCTGGTACTC		89
PVR	NM_017076	F: CCAGTGTGGCGAACATAGCA R: CCTTTCGACAATGCGGAATT		68
Atf3	NM_012912	F: TTCCTTCCCACCAAAAACCA R: CCACAGTGCAGACACCTTCCT		81
Myc	NM_012603	F: GCTTCGAAACTCTGGTGCATAA R: AACCGTTCTCCTTACACTCGAGAT		73
Dusp1	NM_053769	F: TCGAGCCCCTCTGACAAAAC R: AAACAGGCAAGGGAAGAAACTG		72
IL6	NM_012589	F: AATGGAGAAGTTAGAGTCACAGAAGGA R: CCGAGTAGACCTCATAGTGACCTTT		106
Ptgs2	NM_017232	F: GAAGGCCCCGTAGGTATTTTA R: CTCGATGCACAAATTCTAATGC		90
Epha2	NM_001108977	F: CTGGCCGACTTGTAGGAGACTT R: ACAGCGACAGTTGTCCAAAGG		69
Hbegf	NM_012945	F: CTGGCCCGCCTCTCTTG R: ACTACAGATGGGTTACAGAGCAGATG		75
Has2	NM_013153	F: GCCTTGTGTGCAGTCCCATT R: GCTTCACCACAAGGCTTCTGT		72
Adm	NM_012715	F: GGCAGAGGAACCCAAGATCA R: CCTGCCCGGAGAGAGTATCA		75
Loxl2	NM_001106047	F: CGATTGCCACCTCCTTGCTA R: CCAGAGCCCTGCCCCTAA		77
Nrn1	NM_053346	F: AAGAGGTTGGGTATAGTAGGACAGGTT R: CGACGACAATAGCAGGTGAAAC		84
Fos	NM_022197	F: CTCTTCAGCGTCCATGTTCA R: TGTCAGAACATTCAGACCACCT		70
GAPDH	NM_017008	F: TGGCCTCCAAGGAGTAAGAAAC R: GGCCTCTCTCTTGCTCTCAGTATC		69

**Table 6:** Validation of Microarray Gene Expression at 1 h by qPCR.

Gene	Fold change (Mean±SD)	
	Microarray (n = 3)	RT-qPCR (n = 3)
Areg#	5.47±2.50*	5.51±2.46*
Atf3	5.72±1.37*	6.94±1.91*
IL6	3.20±1.36*	3.38±2.27*
Hbegf	3.76±0.73*	4.14±1.95*
Egr2#	2.92±0.74*	3.02±1.13*
Myc	3.11±0.40*	2.96±0.49*
Dusp1	2.97±0.53*	4.08±1.54*
Has2	2.49±0.76*	2.56±1.23*
Ptgs2	2.37±0.63*	4.91±1.29*
Jun#	2.35±0.48*	2.40±0.08*
PVR#	2.04±0.49*	2.01±0.39*
Epha2	2.02±0.41*	1.90±0.13*

# Genes validated at both 1 and 24 h.

* P<0.05.

**Table 7:** Validation of Microarray Gene Expression at 24 h by qPCR.

Gene	Fold change (Mean±SD)	
	Microarray (n = 3)	RT-qPCR (n = 3)
Nrn1	2.60±1.02*	3.07±0.93*
Areg#	1.89±0.29*	2.56±0.61*
Loxl2	2.05±0.14*	2.57±1.37*
Jun#	1.25±0.20*	1.56±0.27*
PVR#	1.18±0.10*	1.44±0.31*
Adm	0.46±0.18*	0.61±0.41
Egr2#	0.40±0.13*	0.46±0.18*
Fos	0.33±0.08*	0.35±0.06*

# Genes validated at both 1 and 24 h.

* P<0.05.

## Discussion

Herein, we first report the differential gene expression profile of Müller cells responding to cyclic mechanical stretching for 1 and 24 h. We identified a number of genes related with response to stimulus, cell proliferation, tissue remodeling, and vasculogenesis and also highlighted some pathways such as MAPK pathway that were significantly involved and that might account for mechanisms of the effects of mechanical forces on Müller cells.

In this study, we used the Flexcell vacuum-driven system to stretch Müller cells by subjecting flexible-bottom culture dishes to distension [8], which has become a standard model for studying the effects of mechanical forces on a variety of ocular cell types, including trabecular meshwork cells [9], retinal microvascular endothelial cells [10], lamina cribrosa cells [11], scleral fibroblasts [12], [13], and retinal pigment epithelial cells [14]. Although there exist some differences in the stretching regimens employed in these studies, elongation of 15% [9], [11], [12] and cyclic stretching [9], [11] was a popular regimen, and axial length elongation by 15% can be observed almost only in cases of pathological myopia. This is why we selected this regimen in this study.

Microarray data analysis identified more significantly differentially expressed genes under mechanical stretching in Müller cells at 24 h than at 1 h. However, when comparing the differentially expressed genes between these two time points, we were surprised to find that there were no overlapped differential genes except for Nr4a1 and Egr2. Furthermore, these two genes were upregulated at 1 h but downregulated at 24 h. Another interesting finding was that at 1 h, 86% of differential genes were upregulated, whereas at 24 h, 74% of differential genes were downregulated. These data revealed that gene expression induced in Müller cells by mechanical stretching at an early time (1 h) was temporary, suggesting a possible distinct response pattern to mechanical stretching at different stages.

Gene ontology analysis showed that numerous biological processes were involved in Müller cells after stretching, suggesting the active response of Müller cells to mechanical strain. This confirmed the mechanoresponsivity of Müller cells, which is consistent with a previous report [7], though cellular reactions might vary widely under mechanical stretching in Müller cells in confluent cultures, compared to those in retinal tissues. Herein, we mainly focus on those genes that are related to cell proliferation, tissue remodeling, and vasculogenesis because these might participate in pathological processes of ocular diseases in which the retina is overstretched.

The proliferation of Müller cells has been suggested to play a central role in the development of epiretinal membranes associated with proliferative vitreoretinopathy [5], [15]. We identified two highly differentially expressed genes that were related to proliferation–Areg and Atf3. Areg, which is significantly upregulated at both 1 and 24 h, is a member of the epidermal growth factor family. Previous studies have shown that it could reactivate astrocytes and promote cell proliferation [16], [17], [18]. Atf3, which is significantly upregulated at only 1 h, is a member of the mammalian activation transcription factor/cAMP responsive element-binding protein family of transcription factors. It responded to cellular injury [19] and could enhance cell proliferation [20], [21], [22]. Thus, it can be inferred that Müller cells could sense the mechanical traction in proliferative vitreoretinopathy and were activated to proliferate, contributing to the development of epiretinal membranes.

In pathological myopia, during the progressive distension of the posterior pole, the retina, choroid, and sclera are subjected to constant mechanical force, as a result of which tissue remodeling occurs [1], [2]. Active remodeling of the sclera in myopia has been intensively studied [23], [24]. Scleral fibroblasts are responsive to mechanical strain [13], and they regulate extracellular matrix synthesis [12]. In comparison, retinal remodeling under mechanical stretching has attracted little attention. Herein, we identified some tissue remodeling related genes, for instance, PVR and Loxl 2. PVR belongs to a transmembrane glycoprotein belonging to the immunoglobulin superfamily, and its expression could promote the production of matrix metalloproteinases-2 [25], a well-known regulator of tissue remodeling [26]. Loxl 2, a member of the lysyl oxidase gene family, was also involved in matrix remodeling [27], [28].

Mechanical force was also one postulated mechanism of myopic choroidal neovascularization during the progressive and excessive elongation of the anteroposterior axis [2]. Mechanical stretching induced the expression and secretion of angiogenic factors in retinal pigment epithelial cells [14]. Here, we show that mechanical stretching induces some other angiogenic factors in Müller cells, such as Epha2 and Nrn1. Soluble Epha2 receptor could inhibit retinal neovascularization [29], and it might become an effective target for ocular neovasculatures [30]. Nrn1, a neurotrophic factor, was recently identified to function as a novel angiogenic factor [31].

The mechanisms of mechanosensing in Müller cells in response to stretching remain unclear. Using KEGG pathway analysis, we identified several significantly changed pathways at 1 and 24 h. Interestingly, only the MAPK pathway was involved at both time points. A previous study also reported that the MAPK pathway was activated in stretched Müller cells [7]. All these data highlight the MAPK pathway as a possible key pathway underlying the mechanosensitivity of Müller cells subjected to mechanical stretching. Moreover, this pathway has been intensively studied in stretched vascular cells [32], [33], [34]. Other pathways such as the TGF-beta signaling pathway and nitrogen metabolism were also triggered in stretched vascular cells [32], [34].

The results of this study should be interpreted with caution because stretching during pathology is likely to be much slower than in our experiments. It is unknown whether the changes we found would occur during much slower stretching in vivo.

### Conclusion

In summary, this study identified several differentially expressed genes and related pathways in Müller cells subjected to mechanical stretching. These results indicate the complex mechanoresponsive nature of Müller cells, and they provide novel insights into possible molecular mechanisms that would account for many retinal diseases in which the retina is often subjected to mechanical forces, such as degenerative axial myopia and proliferative vitreoretinopathy.

## Materials and Methods

### Ethics Statement

The animals were cared for in accordance with the ARVO Statement for the Use of Animals in Ophthalmic and Vision Research. The protocols were reviewed and approved by the Animal Ethics Committee of Fudan University (Shanghai, China).

### Primary Müller Cell Culture

Müller cells were prepared from Sprague-Dawley rats on postnatal days 1–3. Briefly, isolated retinas were digested with 0.25% trypsin (Invitrogen, Carlsbad, CA), and dissociated retinal cells were then cultured at 37°C in 5% CO_2_ and 95% air in Dulbecco’s modified Eagle’s medium (DMEM) supplemented with 20% fetal bovine serum (Invitrogen, Carlsbad, CA) and 100 U/mL penicillin/100 µg/mL streptomycin. Confluent cultures were passaged no more than four times, and cultures with the same number of passages were used for each independent experiment [35]. Cells at passages 2 to 4 were used for experiments. Isolated cells were confirmed by positive staining of three Müller cell markers, antibodies glutamine synthetase [36], vimentin, and SOX9 (data not shown).

### Application of Mechanical Stretching

To apply mechanical strain, the Müller cells were plated on type-I collagen-coated flexible silicone bottom plates (Flexcell International, USA) at an initial density of 2 × 10^5^ cells per well (9.32 cm^2^). After seeding for 24 h, the cells were pretreated with serum-free DMEM for 24 h prior to the experiments to arrest their growth and to synchronize them. The Müller cells were then subjected to 15% cyclic stretching (strain magnitudes, 15%; frequency, 1 Hz; duration, 1 h and 24 h) that was produced by a computer-controlled vacuum (FX-4000T Strain Unit, Flexcell International) as previously described [37]. Briefly, the silicone bottom plates with cultured cells were placed on a vacuum manifold situated in an incubator. When a vacuum was applied to the bottoms of plates, controlled by a computer, the silicone membranes were deformed to a prearranged elongation percentage and returned to their original conformation once the vacuum was released. During this course, Müller cells were tightly attached to the silicone membrane surface, and the deformation of the membrane is directly transmitted to the cells. Müller cells cultured under the same conditions but with no applied mechanical strain were considered as the unstretched control.

### RNA Isolation

At each time point (1 and 24 h), three totally independent experiments (3 stretched samples and 3 control samples) were conducted. After being mechanically strained, as described above, Müller cells were extensively washed with cold PBS and total RNA was extracted using TRIZOL Reagent (Life Technologies, Carlsbad, CA, US) following the manufacturer’s instructions, and RNA quality was confirmed using the Agilent 2100 Bioanalyzer. The qualified total RNA was further purified by RNeasy micro kit (QIAGEN, GmBH, Germany) and RNase-Free DNase Set (QIAGEN, GmBH, Germany).

### Microarray Hybridization and Analysis

The total RNA was amplified, labeled, and purified by using GeneChip 3′IVT Express Kit (Affymetrix, Santa Clara, CA, US) following the manufacturer’s instructions to obtain biotin-labeled cRNA. Array hybridization and washing was performed using Affymetrix Rat Genome 230 2.0 Array Hybridization, Wash, and Stain Kit (Affymetrix, Santa Clara, CA, US) in Hybridization Oven 645 (Affymetrix, Santa Clara, CA, US) and Fluidics Station 450 (Affymetrix, Santa Clara, CA, US) following the manufacturer’s instructions. The slides were scanned using GeneChip® Scanner 3000 (Affymetrix, Santa Clara, CA, US) and Command Console Software 3.1 (Affymetrix, Santa Clara, CA, US) with default settings. Before the statistical analyses, all microarrays were subjected to quality and filtering criteria. All microarray data have been deposited in the NIH/NLM Gene Expression Omnibus (GEO, http://www.ncbi.nlm.nih.gov/projects/geo/provided in the public domain by the National Center for Biotechnology Information, Bethesda, MD) and are accessible through GEO Series accession number GSE 43516. The raw data were normalized using MAS 5.0 algorithm, Gene Spring Software 11.5 (Agilent Technologies, Santa Clara, CA, US).

Data analysis was carried out by using GeneSpring GX 11.5 Software. It was also used for performing gene hierarchical clustering. Student’s t-test (GeneSpring GX11.5) was used to identify genes that were differentially expressed between the stretched and the control groups at the level of significance (p<0.05), but we mainly focused on those genes that showed more than a twofold change in expression. Gene Ontology analysis and the Kyoto Encyclopedia of Genes and Genomes (KEGG) were used to select, annotate, and visualize these genes by function and pathway.

### Real-Time PCR

RNA extracted for the microarray experiments was used to generate cDNA for qPCR using SuperScript III Reverse Transcriptase (Invitrogen, Carlsbad, CA) as per the manufacturer’s instructions. The qPCR was sequenced using the ABI 7900 HT Sequence Detection System (Applied Biosystems). The reactions were set up with 5 µL SYBR Green PCR Master Mix (Takara, Shuzo, Kyoto, Japan), 0.4 µL 10 µM primer mixture, and 5 ng cDNA template. Real-time PCR was performed under the following conditions: 50°C for 120 s, 95°C for 15 s followed by 40 cycles at 95°C for 15 s, and 60°C for 60 s. The relative mRNA levels of the target genes were normalized to GAPDH. The sequences of the primers used for the amplifications (Shenggong Company, China) are shown in Table 5. Statistical analysis was performed using Student’s t-test (Stata, ver. 10.0; Stata Corporation, College Station, TX, USA), and a value of p<0.05 was considered significant.

## Supporting Information

Table S1 **Up- and downregulated genes in Müller cells after stretching for 1 h.**
(DOC)

Table S2 **Up- and downregulated Genes in Müller cells after stretching for 24 h.**
(DOC)

Table S3 **Significantly Upregulated Gene Categories after stretching for 1 h.**
(DOCX)

Table S4 **Significantly downregulated Gene Categories after stretching for 24 h.**
(DOCX)
