# Supplementary material for: Gene Expression Changes under Cyclic Mechanical Stretching in Rat Retinal Glial (Müller) Cells
Source: PLoS One. 2013 May 27;8(5):e63467. doi: 10.1371/journal.pone.0063467 (PMC3664568; doi:10.1371/journal.pone.0063467)
Supplement: Table S1 — Up- and downregulated genes in Müller cells after stretching for 1 h. (DOC) [file pone.0063467.s001.doc]

| Table S1. Up- and downregulated genes (p<0.05) in Müller cells after stretching for 1 h | | | | |
| --- | --- | --- | --- | --- |
| **ProbeSetID** | **Accession#** | **Gene** | **GeneTitle** | **fold^a^** |
| 1369067_at | NM_017352 /// NM_031628 | Nr4a3 | nuclear receptor subfamily 4, group A, member 3 | 15.14 |
| 1368124_at | NM_133578 | Dusp5 | dual specificity phosphatase 5 | 8.16 |
| 1381029_at | NM_001008826 /// NM_001166403 /// NR_002597 | LOC360231 /// RT1-T24-3 /// RT1-T24-4 | MHC class I RT1.O type 149 processed pseudogene /// RT1 class I, locus T24, gene 3 /// RT1 class I, locus T24, gene 4 | 7.29 |
| 1393389_at | NM_017352 /// NM_031628 | Nr4a3 | nuclear receptor subfamily 4, group A, member 3 | 6.99 |
| 1369268_at | NM_012912 | Atf3 | activating transcription factor 3 | 5.72 |
| 1369871_at | NM_017123 | Areg | amphiregulin | 5.47 |
| 1377869_at | NM_138526 | Ccrn4l | CCR4 carbon catabolite repression 4-like (S. cerevisiae) | 5.43 |
| 1386995_at | NM_017259 | Btg2 | BTG family, member 2 | 5.25 |
| 1386396_at | NM_001108510 | Dusp8 | dual specificity phosphatase 8 | 5.10 |
| 1386935_at | NM_024388 | Nr4a1 | nuclear receptor subfamily 4, group A, member 1 | 4.82 |
| 1371248_at | XM_001056859 /// XM_574984 | Sprr1al | small proline-rich protein 1A-like | 4.62 |
| 1386994_at | NM_017259 | Btg2 | BTG family, member 2 | 4.43 |
| 1369587_at | NM_021689 | Ereg | epiregulin | 4.05 |
| 1368983_at | NM_012945 | Hbegf | heparin-binding EGF-like growth factor | 3.76 |
| 1373093_at | NM_001014071 | Errfi1 | ERBB receptor feedback inhibitor 1 | 3.66 |
| 1370997_at | NM_031707 | Homer1 | homer homolog 1 (Drosophila) | 3.60 |
| 1377064_at | NM_053883 | Dusp6 | dual specificity phosphatase 6 | 3.53 |
| 1369191_at | NM_012589 | Il6 | interleukin 6 | 3.20 |
| 1387024_at | NM_053883 | Dusp6 | dual specificity phosphatase 6 | 3.17 |
| 1368308_at | NM_012603 | Myc | myelocytomatosis oncogene | 3.11 |
| 1368147_at | NM_053769 | Dusp1 | dual specificity phosphatase 1 | 3.07 |
| 1376828_at | NM_001079890 | Gprc5a | G protein-coupled receptor, family C, group 5, member A | 3.06 |
| 1382778_at | NM_053883 | Dusp6 | Dual specificity phosphatase 6 | 3.03 |
| 1388686_at | NM_153724 | Rcan1 | regulator of calcineurin 1 | 2.99 |
| 1368146_at | NM_053769 | Dusp1 | dual specificity phosphatase 1 | 2.97 |
| 1368860_at | NM_017180 | Phlda1 | pleckstrin homology-like domain, family A, member 1 | 2.97 |
| 1387306_a_at | NM_053633 | Egr2 | early growth response 2 | 2.92 |
| 1387410_at | NM_019328 | Nr4a2 | nuclear receptor subfamily 4, group A, member 2 | 2.78 |
| 1368489_at | NM_012953 | Fosl1 | fos-like antigen 1 | 2.75 |
| 1369007_at | NM_019328 | Nr4a2 | nuclear receptor subfamily 4, group A, member 2 | 2.72 |
| 1387260_at | NM_053713 | Klf4 | Kruppel-like factor 4 (gut) | 2.71 |
| 1387769_a_at | NM_013058 | Id3 | inhibitor of DNA binding 3 | 2.65 |
| 1371193_at | NM_053382 | Tnfaip6 | tumor necrosis factor alpha induced protein 6 | 2.56 |
| 1372510_at | NM_001047858 | Srxn1 | sulfiredoxin 1 homolog (S. cerevisiae) | 2.56 |
| 1374404_at | NM_021835 | Jun | Jun oncogene | 2.55 |
| 1368247_at | NM_031971 /// NM_212504 | Hspa1a /// Hspa1b | heat shock 70kD protein 1A /// heat shock 70kD protein 1B (mapped) | 2.51 |
| 1387548_at | NM_013153 | Has2 | hyaluronan synthase 2 | 2.49 |
| 1392264_s_at | NM_012620 | Serpine1 | serine (or cysteine) peptidase inhibitor, clade E, member 1 | 2.48 |
| 1374864_at | NM_001012046 | Spry2 | sprouty homolog 2 (Drosophila) | 2.43 |
| 1370912_at | NM_031971 | Hspa1a | heat shock 70kD protein 1A | 2.43 |
| 1387491_at | NM_024381 | Gk | glycerol kinase | 2.38 |
| 1368527_at | NM_017232 | Ptgs2 | prostaglandin-endoperoxide synthase 2 | 2.37 |
| 1389528_s_at | NM_021835 | Jun | Jun oncogene | 2.35 |
| 1369788_s_at | NM_021835 | Jun | Jun oncogene | 2.22 |
| 1372385_at | XM_002728821 | LOC100360845 | hypothetical protein LOC100360845 | 2.16 |
| 1393550_at | NM_001110860 /// NM_017334 | Crem | cAMP responsive element modulator | 2.15 |
| 1372725_at | NM_001014094 | Plscr2 | phospholipid scramblase 2 | 2.14 |
| 1367795_at | NM_019242 | Ifrd1 | interferon-related developmental regulator 1 | 2.12 |
| 1379100_at | NM_001169116 | RGD1306119 | similar to transcriptional regulating protein 132 | 2.11 |
| 1368519_at | NM_012620 | Serpine1 | serine (or cysteine) peptidase inhibitor, clade E, member 1 | 2.09 |
| 1371019_at | NM_023985 | Trib1 | tribbles homolog 1 (Drosophila) | 2.05 |
| 1384331_at | NM_001047858 | Srxn1 | sulfiredoxin 1 homolog (S. cerevisiae) | 2.05 |
| 1370177_at | NM_017076 | PVR | poliovirus receptor | 2.04 |
| 1374143_at | NM_001108977 | Epha2 | Eph receptor A2 | 2.02 |
| 1368223_at | NM_024400 | Adamts1 | ADAM metallopeptidase with thrombospondin type 1 motif, 1 | 1.98 |
| 1387060_at | NM_031642 | Klf6 | Kruppel-like factor 6 | 1.96 |
| 1391010_at | NM_012934 | Dpysl3 | dihydropyrimidinase-like 3 | 1.94 |
| 1380229_at | NM_001130573 | Maff | v-maf musculoaponeurotic fibrosarcoma oncogene homolog F (avian) | 1.94 |
| 1373767_at | NM_001008363 | Zfand2a | zinc finger, AN1-type domain 2A | 1.91 |
| 1372069_at | NM_001037197 | Kank1 | KN motif and ankyrin repeat domains 1 | 1.87 |
| 1387269_s_at | NM_017350 /// NM_134352 | Plaur | plasminogen activator, urokinase receptor | 1.85 |
| 1387068_at | NM_019361 | Arc | activity-regulated cytoskeleton-associated protein | 1.85 |
| 1367802_at | NM_001193568 /// NM_001193569 /// NM_019232 | Sgk1 | serum/glucocorticoid regulated kinase 1 | 1.82 |
| 1367894_at | NM_022392 | Insig1 | insulin induced gene 1 | 1.80 |
| 1373928_at | NM_001004091 | Il17re | interleukin 17 receptor E | 1.79 |
| 1373866_at | NM_001009671 | Coq10b | coenzyme Q10 homolog B (S. cerevisiae) | 1.79 |
| 1384991_a_at | NM_001035000 | Hdac10 | histone deacetylase 10 | 1.75 |
| 1368488_at | NM_053727 | Nfil3 | nuclear factor, interleukin 3 regulated | 1.75 |
| 1368804_at | NM_022196 | Lif | leukemia inhibitory factor | 1.71 |
| 1383519_at | NM_012735 | Hk2 | Hexokinase 2 | 1.70 |
| 1387408_at | NM_134457 | Siah2 | seven in absentia 2 | 1.68 |
| 1392579_at | NM_001014216 | Obfc2a | oligonucleotide/oligosaccharide-binding fold containing 2A | 1.68 |
| 1368868_at | NM_001033653 /// NM_057103 | Akap12 | A kinase (PRKA) anchor protein 12 | 1.68 |
| 1371259_at | XM_001067130 /// XM_227525 | Ngf | nerve growth factor (beta polypeptide) | 1.67 |
| 1387951_at | NM_022269 | Cd55 | Cd55 molecule | 1.67 |
| 1385827_at | NM_207615 | Clcf1 | cardiotrophin-like cytokine factor 1 | 1.67 |
| 1378782_at | XM_002725889 /// XM_002729007 | LOC100362324 | DEAH (Asp-Glu-Ala-His) box polypeptide 29 | 1.65 |
| 1399022_at | NM_001106913 | Clk1 | CDC-like kinase 1 | 1.63 |
| 1370174_at | NM_133546 | Ppp1r15a | protein phosphatase 1, regulatory (inhibitor) subunit 15A | 1.62 |
| 1368596_at | NM_021693 | Sik1 | salt-inducible kinase 1 | 1.62 |
| 1388589_at | NM_001108733 | Dot1l | DOT1-like, histone H3 methyltransferase (S. cerevisiae) | 1.61 |
| 1387395_at | NM_017161 | Adora2b | adenosine A2B receptor | 1.61 |
| 1368851_at | NM_012555 | Ets1 | v-ets erythroblastosis virus E26 oncogene homolog 1 (avian) | 1.60 |
| 1372326_at | NM_017102 | Slc2a3 | Solute carrier family 2 (facilitated glucose transporter), member 3 | 1.59 |
| 1389297_at | NM_138528 | Ero1l | ERO1-like (S. cerevisiae) | 1.58 |
| 1398302_at | NM_022530 | Prl7a3 | prolactin family 7, subfamily a, member 3 | 1.55 |
| 1382873_at | NM_001107712 | Cttnbp2nl | CTTNBP2 N-terminal like | 1.55 |
| 1372144_at | NM_001108004 | Dnajb5 | DnaJ (Hsp40) homolog, subfamily B, member 5 | 1.55 |
| 1369737_at | NM_001110860 /// NM_017334 | Crem | cAMP responsive element modulator | 1.55 |
| 1370108_a_at | NM_053514 | Lin7a | lin-7 homolog a (C. elegans) | 1.52 |
| 1379910_at | NM_001191930 /// XM_001072323 /// XM_573497 | Uap1 | UDP-N-acteylglucosamine pyrophosphorylase 1 | 1.52 |
| 1370314_at | NM_031148 | Slc20a1 | solute carrier family 20 (phosphate transporter), member 1 | 1.52 |
| 1369738_s_at | NM_001110860 /// NM_017334 | Crem | cAMP responsive element modulator | 1.52 |
| 1387750_at | NM_053530 | Twist1 | twist homolog 1 (Drosophila) | 1.51 |
| 1391231_at | NM_001012220 | Catsper2 | cation channel, sperm associated 2 | 1.51 |
| 1375852_at | NM_013134 | Hmgcr | 3-hydroxy-3-methylglutaryl-Coenzyme A reductase | 1.51 |
| 1371542_at | NM_001007004 | Tuba4a | tubulin, alpha 4A | 1.49 |
| 1378925_at | NM_001110860 /// NM_017334 | Crem | cAMP responsive element modulator | 1.49 |
| 1368303_at | NM_031678 | Per2 | period homolog 2 (Drosophila) | 1.49 |
| 1385702_at | NM_001012029 | Ifi204 | interferon activated gene 204 | 1.49 |
| 1385566_at | NM_001011974 | Akap2 | A kinase (PRKA) anchor protein 2 | 1.48 |
| 1369415_at | NM_053328 | Bhlhe40 | basic helix-loop-helix family, member e40 | 1.48 |
| 1387714_at | NM_001110860 /// NM_017334 | Crem | cAMP responsive element modulator | 1.47 |
| 1382511_at | NM_001100778 | E2f1 | E2F transcription factor 1 | 1.46 |
| 1384687_at | NM_001003401 | Enc1 | ectodermal-neural cortex 1 | 1.45 |
| 1374903_at | NM_001001511 | Gcnt2 | glucosaminyl (N-acetyl) transferase 2, I-branching enzyme | 1.44 |
| 1394359_at | NM_001047852 | Bclaf1 | BCL2-associated transcription factor 1 | 1.44 |
| 1373106_at | NM_001036626 | Zfp36l2 | zinc finger protein 36, C3H type-like 2 | 1.44 |
| 1379450_at | NM_001107712 | Cttnbp2nl | CTTNBP2 N-terminal like | 1.44 |
| 1372745_at | NM_053326 | Pdlim5 | PDZ and LIM domain 5 | 1.44 |
| 1386065_at | NM_001109364 | Ankrd57 | ankyrin repeat domain 57 | 1.43 |
| 1369958_at | NM_022542 | Rhob | ras homolog gene family, member B | 1.42 |
| 1368050_at | NM_053662 | Ccnl1 | cyclin L1 | 1.42 |
| 1372417_at | NM_001007735 | Sertad1 | SERTA domain containing 1 | 1.42 |
| 1373989_at | NM_001007754 /// NM_001037555 | Rassf1 | Ras association (RalGDS/AF-6) domain family member 1 | 1.41 |
| 1394710_at | NM_001025705 | Azi2 | 5-azacytidine induced 2 | 1.41 |
| 1380168_at | NM_001108299 | Etv4 | ets variant 4 | 1.41 |
| 1371754_at | NM_145677 | Slc25a25 | solute carrier family 25 (mitochondrial carrier, phosphate carrier), member 25 | 1.41 |
| 1393086_at | NM_001107213 | Uba6 | ubiquitin-like modifier activating enzyme 6 | 1.40 |
| 1375360_at | NM_013216 | Rheb | Ras homolog enriched in brain | 1.40 |
| 1384254_at | XM_001074167 /// XM_574086 | Otud1 | OTU domain containing 1 | 1.40 |
| 1368874_a_at | NM_022386 | Mafg | v-maf musculoaponeurotic fibrosarcoma oncogene homolog G (avian) | 1.40 |
| 1369959_at | NM_017172 | Zfp36l1 | zinc finger protein 36, C3H type-like 1 | 1.39 |
| 1393138_at | NM_138875 | Jund | jun D proto-oncogene | 1.39 |
| 1374139_at | NM_001025682 | Cdr2 | cerebellar degeneration-related 2 | 1.39 |
| 1371785_at | NM_181086 | Tnfrsf12a | tumor necrosis factor receptor superfamily, member 12a | 1.38 |
| 1384392_at | NM_181087 | Cyp26b1 | cytochrome P450, family 26, subfamily b, polypeptide 1 | 1.36 |
| 1388722_at | NM_001108441 | Dnajb1 | DnaJ (Hsp40) homolog, subfamily B, member 1 | 1.36 |
| 1391815_at | NM_001108641 | Rpusd3 | RNA pseudouridylate synthase domain containing 3 | 1.35 |
| 1388868_at | NM_001106356 | Zfand5 | zinc finger, AN1-type domain 5 | 1.34 |
| 1380106_at | NM_001110793 /// NM_058210 | Sf1 | splicing factor 1 | 1.34 |
| 1367960_at | NM_019186 | Arl4a | ADP-ribosylation factor-like 4A | 1.34 |
| 1389129_at | NM_171996 /// XM_001062691 /// XM_002724662 /// XM_002727755 /// XM_220262 | Decr2 /// Rab11fip3 | 2,4-dienoyl CoA reductase 2, peroxisomal /// RAB11 family interacting protein 3 (class II) | 1.34 |
| 1380619_at | NM_001108822 | RGD1305537 | similar to RIKEN cDNA 3110001I22 | 1.34 |
| 1382214_at | NM_001107444 | Nhlrc2 | NHL repeat containing 2 | 1.34 |
| 1389417_at | NM_001107174 | Rbbp5 | retinoblastoma binding protein 5 | 1.33 |
| 1376410_at | NM_001105925 | Mmp17 | matrix metallopeptidase 17 | 1.33 |
| 1370848_at | NM_138827 | Slc2a1 | solute carrier family 2 (facilitated glucose transporter), member 1 | 1.32 |
| 1374797_at | NM_001107293 | Eaf1 | ELL associated factor 1 | 1.32 |
| 1373816_at | NM_134460 | Ap1g1 | adaptor-related protein complex 1, gamma 1 subunit | 1.32 |
| 1374484_at | NM_001013865 | Tmem39a | transmembrane protein 39a | 1.31 |
| 1383302_at | NM_001108441 | Dnajb1 | DnaJ (Hsp40) homolog, subfamily B, member 1 | 1.31 |
| 1381564_at | NM_001105993 | Glmn | glomulin, FKBP associated protein | 1.31 |
| 1375533_at | NM_001015004 | Vgll4 | vestigial like 4 (Drosophila) | 1.30 |
| 1385361_at | NM_001141935 | Atp10a | ATPase, class V, type 10A | 1.30 |
| 1389253_at | NM_001025623 | Vnn1 | vanin 1 | 1.30 |
| 1382154_at | NM_057115 | Ptpn12 | protein tyrosine phosphatase, non-receptor type 12 | 1.29 |
| 1371130_at | NM_019225 | Slc1a3 | solute carrier family 1 (glial high affinity glutamate transporter), member 3 | 1.28 |
| 1370931_at | NM_177419 | Xrcc5 | X-ray repair complementing defective repair in Chinese hamster cells 5 | 1.28 |
| 1379853_at | NM_001131002 | Hspb11 | heat shock protein family B (small), member 11 | 1.28 |
| 1390972_at | NM_001013926 | Tprkb | Tp53rk binding protein | 1.28 |
| 1382379_at | NM_053588 | Rnf138 | ring finger protein 138 | 1.27 |
| 1368118_at | NM_031328 | Bcl10 | B-cell CLL/lymphoma 10 | 1.27 |
| 1368132_at | NM_133317 | Tob1 | transducer of ErbB-2.1 | 1.27 |
| 1374940_at | NM_001107069 | Usp36 | ubiquitin specific peptidase 36 | 1.27 |
| 1398321_a_at | XM_001060689 /// XM_243912 | Col12a1 | collagen, type XII, alpha 1 | 1.26 |
| 1388858_at | NM_001100674 | Map2k3 | mitogen activated protein kinase kinase 3 | 1.25 |
| 1375537_at | NM_001029897 | Strn3 | striatin, calmodulin binding protein 3 | 1.25 |
| 1387101_at | NM_053623 | Acsl4 | acyl-CoA synthetase long-chain family member 4 | 1.25 |
| 1373917_at | NM_001008344 | Etf1 | Eukaryotic translation termination factor 1 | 1.24 |
| 1391972_a_at | NM_001107163 | Zswim4 | zinc finger, SWIM-type containing 4 | 1.23 |
| 1393365_at | NM_001107465 | Chd1 | chromodomain helicase DNA binding protein 1 | 1.22 |
| 1370357_at | NM_172066 | Slc30a4 | solute carrier family 30 (zinc transporter), member 4 | 1.22 |
| 1373629_at | NM_001107424 | Slc7a6 | solute carrier family 7 (cationic amino acid transporter, y+ system), member 6 | 1.22 |
| 1370680_at | NM_001007149 /// NM_001007150 /// NM_134466 | Stau2 | staufen, RNA binding protein, homolog 2 (Drosophila) | 1.22 |
| 1374976_a_at | NM_031118 | Soat1 | Sterol O-acyltransferase 1 | 1.21 |
| 1387046_at | NM_053792 | Ift172 | intraflagellar transport 172 homolog (Chlamydomonas) | 1.21 |
| 1371350_at | NM_134351 | Mat2a | methionine adenosyltransferase II, alpha | 1.21 |
| 1385809_at | XR_085786 /// XR_086207 | Fastkd5 | FAST kinase domains 5 | 1.20 |
| 1383500_at | XM_001053669 /// XM_230637 | Rrbp1 | ribosome binding protein 1 | 1.20 |
| 1382267_at | NM_001108955 | Fjx1 | four jointed box 1 (Drosophila) | 1.19 |
| 1384268_at | XM_001072068 /// XM_237211 | Ccnyl1 | cyclin Y-like 1 | 1.19 |
| 1375019_at | NM_001108532 | Hnrnph3 | heterogeneous nuclear ribonucleoprotein H3 (2H9) | 1.19 |
| 1399050_at | NM_001105975 | Adss | adenylosuccinate synthase | 1.19 |
| 1371527_at | NM_012843 | Emp1 | epithelial membrane protein 1 | 1.19 |
| 1379262_at | NM_001013960 | Acot9 | acyl-CoA thioesterase 9 | 1.19 |
| 1367727_at | NM_053911 | Cyth2 | cytohesin 2 | 1.19 |
| 1367761_at | NM_133320 | Ndel1 | nuclear distribution gene E-like homolog 1 (A. nidulans) | 1.19 |
| 1371431_at | NM_001170584 /// XM_001062081 /// XM_232343 | Pex5 | peroxisomal biogenesis factor 5 | 1.19 |
| 1379302_at | NM_001106631 | Rbpj | recombination signal binding protein for immunoglobulin kappa J region | 1.18 |
| 1374500_at | NM_001024305 | Prpf38b | PRP38 pre-mRNA processing factor 38 (yeast) domain containing B | 1.18 |
| 1372133_at | NM_001013434 | Rras2 | related RAS viral (r-ras) oncogene homolog 2 | 1.17 |
| 1378079_at | NM_001107847 | Golga3 | golgi autoantigen, golgin subfamily a, 3 | 1.17 |
| 1383089_at | NM_001004238 | Rab21 | RAB21, member RAS oncogene family | 1.17 |
| 1367614_at | NM_012904 | Anxa1 | annexin A1 | 1.17 |
| 1390577_at | NM_001135875 | Ranbp10 | RAN binding protein 10 | 1.16 |
| 1373182_at | NM_001100813 | Cldn12 | claudin 12 | 1.16 |
| 1374279_at | NM_053757 | Aimp1 | aminoacyl tRNA synthetase complex-interacting multifunctional protein 1 | 1.16 |
| 1398899_at | NM_001012473 | Polr2c | polymerase (RNA) II (DNA directed) polypeptide C | 1.16 |
| 1377860_at | NM_001106665 | Ttc39b | tetratricopeptide repeat domain 39B | 1.16 |
| 1370928_at | NM_001105735 | Litaf | lipopolysaccharide-induced TNF factor | 1.15 |
| 1376161_at | NM_001107238 | Tmed4 | transmembrane emp24 protein transport domain containing 4 | 1.15 |
| 1390689_at | NM_001105984 | Rps6kc1 | ribosomal protein S6 kinase, polypeptide 1 | 1.15 |
| 1370007_at | NM_053849 | Pdia4 | protein disulfide isomerase family A, member 4 | 1.15 |
| 1376446_at | NM_001005556 | Mavs | mitochondrial antiviral signaling protein | 1.15 |
| 1367666_at | NM_080896 | Hnrph1 | heterogeneous nuclear ribonucleoprotein H1 | 1.15 |
| 1388620_at | NM_001025734 | Tada3l | transcriptional adaptor 3 (NGG1 homolog, yeast)-like | 1.15 |
| 1377779_at | NM_001025709 | Pdcl3 | phosducin-like 3 | 1.14 |
| 1370376_a_at | NM_031979 | Csda | cold shock domain protein A | 1.14 |
| 1376576_at | NM_001025650 | Dusp11 | dual specificity phosphatase 11 (RNA/RNP complex 1-interacting) | 1.14 |
| 1398998_at | NM_001106972 | RGD1309748 | similar to CG4768-PA | 1.14 |
| 1382058_at | NM_001013434 | Rras2 | related RAS viral (r-ras) oncogene homolog 2 | 1.13 |
| 1391928_at | NM_001107631 | Gopc | golgi associated PDZ and coiled-coil motif containing | 1.13 |
| 1398802_at | NM_031237 | Ube2d3 | ubiquitin-conjugating enzyme E2D 3 (UBC4/5 homolog, yeast) | 1.13 |
| 1390966_at | NM_001047849 | Agpat6 | 1-acylglycerol-3-phosphate O-acyltransferase 6 (lysophosphatidic acid acyltransferase, zeta) | 1.13 |
| 1390889_at | NM_001109645 | LOC691543 | hypothetical protein LOC691543 | 1.13 |
| 1377848_at | NM_001009633 | Fam175a | family with sequence similarity 175, member A | 1.12 |
| 1368584_a_at | NM_053878 | Cplx2 | complexin 2 | 1.12 |
| 1377644_at | NM_001134421 | RGD1308706 | similar to RIKEN cDNA 4921524J17 | 1.12 |
| 1368470_at | NM_012960 | Ggh | gamma-glutamyl hydrolase (conjugase, folylpolygammaglutamyl hydrolase) | 1.11 |
| 1387807_at | NM_031763 | Pafah1b1 | platelet-activating factor acetylhydrolase, isoform 1b, subunit 1 | 1.11 |
| 1390485_at | NM_001106372 | 40973 | membrane-associated ring finger (C3HC4) 5 | 1.10 |
| 1378387_at | NM_001006962 | Tinf2 | TERF1 (TRF1)-interacting nuclear factor 2 | 1.10 |
| 1389290_at | NM_001106486 | Dnajc10 | DnaJ (Hsp40) homolog, subfamily C, member 10 | 1.10 |
| 1382602_at | NM_001134550 | RGD1565257 | similar to zinc finger protein 650 | 1.09 |
| 1373653_at | NM_001107359 | Mtpap | mitochondrial poly(A) polymerase | 1.09 |
| 1388810_at | NM_001108446 | Abce1 | ATP-binding cassette, sub-family E (OABP), member 1 | 1.09 |
| 1388893_at | NM_001007683 | Glt8d1 | glycosyltransferase 8 domain containing 1 | 1.09 |
| 1373495_at | NM_001106642 | Ube2j1 | ubiquitin-conjugating enzyme E2, J1 (UBC6 homolog, yeast) | 1.08 |
| 1390151_at | XM_001061393 | LOC683034 | hypothetical protein LOC683034 | 1.08 |
| 1368049_at | NM_012670 | Tcp1 | t-complex 1 | 1.08 |
| 1388463_at | NM_001007665 | Tex264 | testis expressed 264 | 1.08 |
| 1388136_at | XM_001072892 /// XM_001080501 /// XM_002726750 /// XM_002726751 /// XM_002729633 /// XM_002729634 | Timm9 | translocase of inner mitochondrial membrane 9 homolog (yeast) | 1.08 |
| 1372552_at | NM_182843 | Acbd3 | acyl-Coenzyme A binding domain containing 3 | 1.07 |
| 1376666_at | XM_001063512 /// XM_225667 | Socs6 | suppressor of cytokine signaling 6 | 1.07 |
| 1371329_at | NM_001033681 | Eif5a | eukaryotic translation initiation factor 5A | 1.05 |
| 1398927_at | NM_001025669 | RGD1307161 | similar to 0610010K06Rik protein | 1.04 |
| 1373229_at | NM_001105843 | Lsm12 | LSM12 homolog (S. cerevisiae) | 0.96 |
| 1382807_at | NM_001014218 | Atg9a | ATG9 autophagy related 9 homolog A (S. cerevisiae) | 0.96 |
| 1374467_at | NM_001039001 | Trap1 | TNF receptor-associated protein 1 | 0.96 |
| 1389907_at | XR_085843 /// XR_086264 | Zbtb8os | zinc finger and BTB domain containing 8 opposite strand | 0.94 |
| 1371424_at | NM_001037196 | Jmjd5 | jumonji domain containing 5 | 0.94 |
| 1370354_at | NM_031339 | Parg | poly (ADP-ribose) glycohydrolase | 0.94 |
| 1373499_at | NR_002704 | Gas5 | growth arrest specific 5 | 0.94 |
| 1393806_at | NM_001109603 | Mansc1 | MANSC domain containing 1 | 0.94 |
| 1399129_at | NM_001169115 | RGD1309765 | similar to hypothetical protein | 0.93 |
| 1388514_at | NM_147209 | Ppm1g | protein phosphatase 1G (formerly 2C), magnesium-dependent, gamma isoform | 0.93 |
| 1399062_at | XM_002725888 /// XM_002729013 | LOC100359861 | mCG51409-like | 0.93 |
| 1372477_at | NM_212542 | Ppp1r11 | protein phosphatase 1, regulatory (inhibitor) subunit 11 | 0.92 |
| 1392454_at | NM_001106203 | Znhit6 | zinc finger, HIT type 6 | 0.92 |
| 1370327_at | NM_139108 | Commd5 | COMM domain containing 5 | 0.92 |
| 1367719_at | NM_001191990 /// XM_001061308 /// XM_576252 | Ubr5 | ubiquitin protein ligase E3 component n-recognin 5 | 0.92 |
| 1372313_at | NM_001139486 | Gpr89 | G protein-coupled receptor 89 | 0.92 |
| 1371809_at | NM_212534 | Mrps18b | mitochondrial ribosomal protein S18B | 0.91 |
| 1368297_at | NM_033442 | Gata2 | GATA binding protein 2 | 0.91 |
| 1367875_at | NM_031030 | Gak | cyclin G associated kinase | 0.91 |
| 1372044_at | NM_001108323 | RGD1310348 | similar to Ser/Thr-rich protein T10 in DGCR region | 0.91 |
| 1371531_at | XM_001053139 | LOC678880 | similar to mammalian retrotransposon derived 8b | 0.91 |
| 1384327_at | NM_001024774 | Def8 | differentially expressed in FDCP 8 homolog (mouse) | 0.91 |
| 1399023_at | NM_001100520 | Ric8a | resistance to inhibitors of cholinesterase 8 homolog A (C. elegans) | 0.91 |
| 1371551_at | NM_001107017 | Traf4 | Tnf receptor associated factor 4 | 0.91 |
| 1383720_at | XM_002727525 /// XM_002727526 /// XM_002727527 /// XM_002727528 /// XM_002727529 /// XM_002730185 | Utx | ubiquitously transcribed tetratricopeptide repeat, X chromosome | 0.91 |
| 1396109_at | NM_001044249 | Antxr1 | anthrax toxin receptor 1 | 0.91 |
| 1383223_at | NM_001015030 | Ubl3 | ubiquitin-like 3 | 0.91 |
| 1370505_at | NM_183325 | Adprh | ADP-ribosylarginine hydrolase | 0.91 |
| 1376609_at | NM_001106160 | RGD1309102 | similar to TRS85 homolog | 0.90 |
| 1373388_at | XR_085589 /// XR_085997 | Sppl3 | signal peptide peptidase 3 | 0.90 |
| 1378191_at | NM_001014785 | Ncbp1 | nuclear cap binding protein subunit 1, 80kDa | 0.90 |
| 1369065_a_at | NM_001110139 /// NM_001110823 /// NR_027839 | Atp2a2 | ATPase, Ca++ transporting, cardiac muscle, slow twitch 2 | 0.90 |
| 1375523_at | XM_002728965 | Marcks | Myristoylated alanine rich protein kinase C substrate | 0.90 |
| 1374284_at | NM_001024275 | Rassf4 | Ras association (RalGDS/AF-6) domain family member 4 | 0.90 |
| 1390863_at | NM_001030024 | Slc19a2 | solute carrier family 19 (thiamine transporter), member 2 | 0.90 |
| 1372804_at | NM_001006959 | RGD1359460 | MMR_HSR1 domain containing protein RGD1359460 | 0.90 |
| 1372155_at | NM_053916 | Trim28 | tripartite motif-containing 28 | 0.90 |
| 1398952_at | NM_001105946 | RGD1564093 | similar to RIKEN cDNA 2310036O22 | 0.89 |
| 1380072_at | NM_001004221 | MGC93975 | similar to 2310044H10Rik protein | 0.89 |
| 1368012_at | NM_022591 | Tep1 | telomerase associated protein 1 | 0.89 |
| 1371876_at | NM_001106138 | Psmg2 | proteasome (prosome, macropain) assembly chaperone 2 | 0.89 |
| 1374733_at | NM_001100830 | Sympk | symplekin | 0.89 |
| 1399134_at | NM_001024322 | Pot1a | protection of telomeres 1A | 0.89 |
| 1388623_at | NM_001011939 | Ehd1 | EH-domain containing 1 | 0.89 |
| 1388588_at | NM_001015013 | Mtvr2 | mammary tumor virus receptor 2 | 0.89 |
| 1374568_at | NM_001108044 | RGD1309492 | similar to mKIAA1737 protein | 0.89 |
| 1388641_at | NM_001011899 | Gart | phosphoribosylglycinamide formyltransferase | 0.89 |
| 1389260_at | NM_001108812 | Ubxn6 | UBX domain protein 6 | 0.88 |
| 1389598_at | NM_001191794 /// XM_001074158 /// XM_236325 | Cln6 | ceroid-lipofuscinosis, neuronal 6 | 0.88 |
| 1368403_at | NM_031094 | Rbl2 | retinoblastoma-like 2 | 0.88 |
| 1373835_at | NM_001012050 | Fbxo8 | F-box protein 8 | 0.88 |
| 1398857_at | NM_172068 | Surf1 | surfeit 1 | 0.88 |
| 1367471_at | NM_001109614 | Polr2e | polymerase (RNA) II (DNA directed) polypeptide E | 0.88 |
| 1371347_at | NM_001025014 | Tmem50b | transmembrane protein 50B | 0.88 |
| 1382303_at | NM_214457 | Phactr1 | phosphatase and actin regulator 1 | 0.88 |
| 1373550_at | NM_001108276 | Trim11 | tripartite motif-containing 11 | 0.88 |
| 1374092_at | NM_001024869 | Wwtr1 | WW domain containing transcription regulator 1 | 0.88 |
| 1398911_at | NM_001034136 | Mrpl2 | mitochondrial ribosomal protein L2 | 0.88 |
| 1373924_at | NM_001013963 | Cpped1 | calcineurin-like phosphoesterase domain containing 1 | 0.88 |
| 1382428_at | NM_001044243 | Tasp1 | taspase, threonine aspartase 1 | 0.88 |
| 1370460_at | NM_145184 | Usp15 | ubiquitin specific peptidase 15 | 0.88 |
| 1375068_at | NM_001077679 | Med22 | mediator complex subunit 22 | 0.87 |
| 1369690_at | NM_021748 | Nsf | N-ethylmaleimide-sensitive factor | 0.87 |
| 1373589_at | NM_001012038 | Mtmr3 | myotubularin related protein 3 | 0.87 |
| 1373460_at | NM_001037215 | Slc35b2 | solute carrier family 35, member B2 | 0.87 |
| 1378080_at | NM_001173374 /// XM_001078805 /// XM_573117 | Sco1 | SCO cytochrome oxidase deficient homolog 1 (yeast) | 0.87 |
| 1382725_at | NM_001108807 | Cops7b | COP9 constitutive photomorphogenic homolog subunit 7B (Arabidopsis) | 0.87 |
| 1372901_at | XM_001054658 /// XM_227139 | Nhlrc3 | NHL repeat containing 3 | 0.87 |
| 1391559_at | NM_001013858 | Tlcd1 | TLC domain containing 1 | 0.87 |
| 1394033_at | NM_019219 | Rbbp9 | retinoblastoma binding protein 9 | 0.86 |
| 1370337_at | NM_031824 | Ctcf | CCCTC-binding factor (zinc finger protein) | 0.86 |
| 1376294_at | XM_001077609 /// XM_573103 | Smcr7 | Smith-Magenis syndrome chromosome region, candidate 7 homolog (human) | 0.86 |
| 1376260_at | NM_001106470 | Mettl14 | methyltransferase like 14 | 0.86 |
| 1372633_at | NM_001106433 | Spg20 | spastic paraplegia 20 (Troyer syndrome) homolog (human) | 0.86 |
| 1398435_at | NM_001107707 | Slc22a15 | solute carrier family 22, member 15 | 0.86 |
| 1376608_at | NM_001107122 | Rnf216 | ring finger protein 216 | 0.86 |
| 1369702_at | NM_001033974 /// NM_021842 | Ensa | endosulfine alpha | 0.86 |
| 1372932_at | NM_001012073 | Rrp1 | ribosomal RNA processing 1 homolog (S. cerevisiae) | 0.86 |
| 1399167_a_at | NM_001108444 | Gab1 | GRB2-associated binding protein 1 | 0.86 |
| 1398375_at | XM_002726673 /// XM_002729591 | LOC100362346 | metastasis associated 3 | 0.86 |
| 1389791_at | NM_001007686 | Cln8 | ceroid-lipofuscinosis, neuronal 8 | 0.86 |
| 1369743_a_at | NM_031594 | P2rx4 | purinergic receptor P2X, ligand-gated ion channel 4 | 0.86 |
| 1376050_at | NM_001106365 | Taf5 | TAF5 RNA polymerase II, TATA box binding protein (TBP)-associated factor | 0.86 |
| 1388609_at | NM_001034149 | Ddi2 | DNA-damage inducible protein 2 | 0.86 |
| 1379337_at | NM_001009673 | Fastkd2 | FAST kinase domains 2 | 0.86 |
| 1373339_at | NM_001122781 | Letmd1 | LETM1 domain containing 1 | 0.86 |
| 1391530_a_at | NM_001100508 | Oxsm | 3-oxoacyl-ACP synthase, mitochondrial | 0.85 |
| 1390851_at | NM_001024247 | Lactb2 | Lactamase, beta 2 | 0.85 |
| 1397824_at | XR_086070 /// XR_086657 | RGD1562407 | similar to WAC | 0.85 |
| 1378041_at | NM_001007796 | Abcb8 | ATP-binding cassette, sub-family B (MDR/TAP), member 8 | 0.85 |
| 1371680_at | NM_001044294 | Gabarapl1 | GABA(A) receptor-associated protein like 1 | 0.85 |
| 1376159_at | NM_001107788 | Gzf1 | GDNF-inducible zinc finger protein 1 | 0.85 |
| 1378014_at | NM_022271 | LOC64038 | Sertolin | 0.85 |
| 1388812_at | NM_001105818 | Tp53i13 | tumor protein p53 inducible protein 13 | 0.85 |
| 1392070_at | NM_001024303 | Lix1l | Lix1 homolog (mouse)-like | 0.85 |
| 1373471_at | NM_001002279 | Rnf166 | ring finger protein 166 | 0.85 |
| 1389247_at | XM_001056048 /// XM_341388 | Polr3a | polymerase (RNA) III (DNA directed) polypeptide A | 0.85 |
| 1373216_at | NM_001198796 /// XM_001079851 /// XM_002727082 | LOC691849 | hypothetical protein LOC691849 | 0.84 |
| 1372942_at | NM_001107493 | Exosc5 | exosome component 5 | 0.84 |
| 1370002_at | NM_021694 | Arhgef1 | Rho guanine nucleotide exchange factor (GEF) 1 | 0.84 |
| 1375913_at | NM_001106196 | Galnt2 | UDP-N-acetyl-alpha-D-galactosamine:polypeptide N-acetylgalactosaminyltransferase 2 (GalNAc-T2) | 0.84 |
| 1384306_at | NM_001108743 | Elk3 | ELK3, member of ETS oncogene family | 0.84 |
| 1398942_at | XR_085872 /// XR_086292 | LOC100125365 | hypothetical protein LOC100125365 | 0.84 |
| 1368785_a_at | NM_001042505 /// NM_019334 | Pitx2 | paired-like homeodomain 2 | 0.84 |
| 1369085_s_at | NM_031117 /// NM_130738 | Snrpn /// Snurf | small nuclear ribonucleoprotein polypeptide N /// SNRPN upstream reading frame | 0.84 |
| 1388522_at | NM_001107141 | Pptc7 | PTC7 protein phosphatase homolog (S. cerevisiae) | 0.84 |
| 1387499_a_at | NM_022247 | Pdcl | phosducin-like | 0.84 |
| 1373982_at | NM_001025626 | RGD1306595 | similar to hypothetical protein | 0.84 |
| 1395526_at | NM_001109620 | Mrpl10 | mitochondrial ribosomal protein L10 | 0.84 |
| 1371980_at | NM_001034922 | Atad3a | ATPase family, AAA domain containing 3A | 0.84 |
| 1382866_at | NM_001108339 | Gatc | glutamyl-tRNA(Gln) amidotransferase, subunit C homolog (bacterial) | 0.84 |
| 1379074_at | NM_001107565 | Oraov1 | oral cancer overexpressed 1 | 0.84 |
| 1370326_at | NM_001004132 /// NM_031077 | Pctk1 | PCTAIRE protein kinase 1 | 0.84 |
| 1370370_at | NM_172040 | Hyal2 | hyaluronoglucosaminidase 2 | 0.83 |
| 1374024_at | XM_001081514 | LOC688211 | hypothetical protein LOC688211 | 0.83 |
| 1389988_at | XM_001081684 /// XM_573223 | Kctd2 | potassium channel tetramerisation domain containing 2 | 0.83 |
| 1392461_at | NM_001106659 | Prpf4 | PRP4 pre-mRNA processing factor 4 homolog (yeast) | 0.83 |
| 1383573_at | XM_001060440 /// XM_225688 | Tshz1 | teashirt zinc finger homeobox 1 | 0.83 |
| 1373136_at | NM_001008308 | Zufsp | zinc finger with UFM1-specific peptidase domain | 0.83 |
| 1399016_at | NM_181081 | Myst2 | MYST histone acetyltransferase 2 | 0.83 |
| 1374860_at | NM_001109451 | LOC684993 | hypothetical protein LOC684993 | 0.83 |
| 1389498_at | NM_001108932 | Tysnd1 | trypsin domain containing 1 | 0.83 |
| 1375444_at | NM_001100719 | Ap3d1 | adaptor-related protein complex 3, delta 1 subunit | 0.83 |
| 1390379_at | NM_001009708 | Lmo4 | LIM domain only 4 | 0.82 |
| 1383715_at | NM_001014191 | Tmem38b | transmembrane protein 38B | 0.82 |
| 1373619_at | XM_001075954 /// XM_341466 | Ankrd10 | ankyrin repeat domain 10 | 0.82 |
| 1384167_at | XM_001057182 | LOC681647 | similar to F43G9.2 | 0.82 |
| 1392385_at | XM_001072953 /// XM_215947 | Ncoa3 | Nuclear receptor coactivator 3 | 0.82 |
| 1377270_a_at | XM_001057512 /// XM_575654 | Dcp1b | DCP1 decapping enzyme homolog b (S. cerevisiae) | 0.82 |
| 1390926_at | NM_001107801 | Zswim3 | zinc finger, SWIM-type containing 3 | 0.82 |
| 1388846_at | NM_001108480 | Bcl2l12 | BCL2-like 12 (proline rich) | 0.81 |
| 1372689_at | NM_001108782 | Tmem103 | transmembrane protein 103 | 0.81 |
| 1387009_at | NM_019152 | Capn1 | calpain 1 | 0.81 |
| 1398912_at | NM_001007696 | mrpl9 | mitochondrial ribosomal protein L9 | 0.81 |
| 1374904_at | NM_053759 | Six1 | SIX homeobox 1 | 0.81 |
| 1372956_at | NM_001034157 | Bat4 | HLA-B associated transcript 4 | 0.81 |
| 1387407_at | NM_133402 | Nap1l3 | nucleosome assembly protein 1-like 3 | 0.81 |
| 1399099_at | NM_001108477 | Hnrnpul1 | heterogeneous nuclear ribonucleoprotein U-like 1 | 0.81 |
| 1375818_at | NM_001106833 | Lactb | lactamase, beta | 0.81 |
| 1377837_at | NM_001004445 | Rnf113a2 | ring finger protein 113A2 | 0.81 |
| 1373946_at | NM_001106576 | Armc10 | armadillo repeat containing 10 | 0.81 |
| 1373378_at | NM_001106100 | Agtpbp1 | ATP/GTP binding protein 1 | 0.81 |
| 1371090_at | NM_023955 | Scamp2 | secretory carrier membrane protein 2 | 0.81 |
| 1378221_at | NM_001005536 | Srfbp1 | Serum response factor binding protein 1 | 0.81 |
| 1382948_at | NM_001191815 /// XM_001054365 /// XM_237535 | Arhgap28 | Rho GTPase activating protein 28 | 0.81 |
| 1376644_at | NM_001107741 | Med19 | mediator complex subunit 19 | 0.80 |
| 1399120_at | NM_001108713 | Trmt5 | TRM5 tRNA methyltransferase 5 homolog (S. cerevisiae) | 0.80 |
| 1389303_at | NM_001014122 | Mif4gd | MIF4G domain containing | 0.80 |
| 1392157_at | NM_001105988 | Plxna2 | plexin A2 | 0.80 |
| 1376061_at | NM_001109030 | RGD1564036 | similar to RIKEN cDNA 3010026O09 | 0.80 |
| 1389771_at | XM_002724787 /// XM_221915 | Zfp853 | zinc finger protein 853 | 0.80 |
| 1371749_at | NM_001130696 | RGD1306001 | similar to 2210021J22Rik protein | 0.80 |
| 1377768_at | XM_001062225 /// XM_214617 | Kctd1 | potassium channel tetramerisation domain containing 1 | 0.80 |
| 1368264_at | NM_057125 | Pex6 | peroxisomal biogenesis factor 6 | 0.80 |
| 1381404_at | NM_001011924 | Mbd1 | Methyl-CpG binding domain protein 1 | 0.79 |
| 1372135_at | NM_001135238 | LOC684352 | similar to twinfilin-like protein | 0.79 |
| 1372637_at | XM_001067331 | LOC683751 | similar to trophinin isoform 1 | 0.79 |
| 1374544_at | NM_001044300 | MGC112715 | platelet receptor Gi24 | 0.79 |
| 1384349_at | NM_001107693 | Pogz | pogo transposable element with ZNF domain | 0.79 |
| 1384016_at | NM_001081447 | RGD1563634 | similar to R31449_3 | 0.79 |
| 1383523_at | NM_001107115 | Arhgef18 | rho/rac guanine nucleotide exchange factor (GEF) 18 | 0.79 |
| 1376909_at | NM_001108862 | Rasl10a | RAS-like, family 10, member A | 0.79 |
| 1390418_at | NM_001039028 | Actr1b | ARP1 actin-related protein 1 homolog B (yeast) | 0.79 |
| 1378326_at | NM_001014257 | Trub2 | TruB pseudouridine (psi) synthase homolog 2 (E. coli) | 0.78 |
| 1373083_at | NM_001034854 | Ppapdc2 | phosphatidic acid phosphatase type 2 domain containing 2 | 0.78 |
| 1388756_at | NM_001039010 | Ppcs | phosphopantothenoylcysteine synthetase | 0.78 |
| 1391699_at | NM_031335 | Polr2f | polymerase (RNA) II (DNA directed) polypeptide F | 0.78 |
| 1387029_at | NM_130409 | Cfh | complement factor H | 0.78 |
| 1378194_a_at | NM_001025127 | Rufy3 | RUN and FYVE domain containing 3 | 0.78 |
| 1371856_at | NM_001103360 | Pnrc2 | proline-rich nuclear receptor coactivator 2 | 0.78 |
| 1383558_at | NM_001109249 | Eefsec | eukaryotic elongation factor, selenocysteine-tRNA-specific | 0.78 |
| 1395579_at | NM_001130039 | Dhx32 | DEAH (Asp-Glu-Ala-His) box polypeptide 32 | 0.78 |
| 1373838_at | NM_022219 | Fut4 | fucosyltransferase 4 (alpha (1,3) fucosyltransferase, myeloid-specific) | 0.78 |
| 1395520_at | NM_001107850 | Rbm28 | RNA binding motif protein 28 | 0.78 |
| 1371778_at | NM_001012060 | Rnf146 | ring finger protein 146 | 0.78 |
| 1384929_at | NM_001109154 | Apob48r | apolipoprotein B48 receptor | 0.77 |
| 1370574_a_at | NM_013016 | Sirpa | signal-regulatory protein alpha | 0.77 |
| 1393074_at | NM_001107981 | RGD1563072 | similar to hypothetical protein FLJ38984 | 0.77 |
| 1381088_at |  | Podn | podocan | 0.77 |
| 1379731_at | NM_001107194 | Pogk | pogo transposable element with KRAB domain | 0.77 |
| 1384852_at | NM_017317 | Rab27a | RAB27A, member RAS oncogene family | 0.77 |
| 1391970_at | NM_175708 | Car11 | carbonic anhydrase 11 | 0.77 |
| 1383405_at | XM_002726078 | LOC100364681 | rCG45515-like | 0.77 |
| 1386247_at | XM_001061047 /// XM_224863 | Snx25 | sorting nexin 25 | 0.77 |
| 1389568_at | NM_001008306 | Calhm2 | calcium homeostasis modulator 2 | 0.77 |
| 1372742_at | NM_001108019 | Ankmy2 | ankyrin repeat and MYND domain containing 2 | 0.76 |
| 1389782_at | NM_001127452 | RGD1305587 | similar to RIKEN cDNA 2010107G23 | 0.76 |
| 1392744_at | NM_001012097 | Atg7 | ATG7 autophagy related 7 homolog (S. cerevisiae) | 0.76 |
| 1393131_at | NM_001106223 | Fiz1 | FLT3-interacting zinc finger 1 | 0.76 |
| 1381919_at | NM_181432 | Hps6 | Hermansky-Pudlak syndrome 6 | 0.76 |
| 1383466_at | NM_001014185 | Sfrs6 | splicing factor, arginine/serine-rich 6 | 0.76 |
| 1384551_at | NM_001107584 | Ranbp6 | RAN binding protein 6 | 0.76 |
| 1383099_at | NM_001106410 | Gpbp1 | GC-rich promoter binding protein 1 | 0.76 |
| 1374159_at | XM_001076120 /// XM_002727139 /// XM_002729977 /// XM_236654 | Als2cl | ALS2 C-terminal like | 0.76 |
| 1384455_at | XM_002726998 | LOC100365921 | rCG31672-like | 0.76 |
| 1381042_at | NM_001108445 | Anapc10 | Anaphase promoting complex subunit 10 | 0.76 |
| 1385519_at | NM_001108657 | Runx1t1 | runt-related transcription factor 1; translocated to, 1 (cyclin D-related) | 0.76 |
| 1369402_at | NM_022681 | Adnp | activity-dependent neuroprotector homeobox | 0.75 |
| 1393132_at | NM_001109301 | RGD1560271 | similar to inhibitor of MyoD family-a | 0.75 |
| 1391595_at | NM_001108154 | Larp6 | La ribonucleoprotein domain family, member 6 | 0.75 |
| 1371897_at | NM_001012349 | Ppapdc3 | phosphatidic acid phosphatase type 2 domain containing 3 | 0.75 |
| 1367828_at | NM_022512 | Acads | acyl-Coenzyme A dehydrogenase, C-2 to C-3 short chain | 0.75 |
| 1392019_at | NM_001044245 | Asap1 | ArfGAP with SH3 domain, ankyrin repeat and PH domain 1 | 0.75 |
| 1388891_at | NM_001134780 | Parvb | Parvin, beta | 0.75 |
| 1370711_a_at | NM_139091 | Nupl1 | nucleoporin like 1 | 0.75 |
| 1373420_at | NM_001006986 | Ecsit | ECSIT homolog (Drosophila) | 0.74 |
| 1377949_s_at | NM_001107613 | Anks1a | ankyrin repeat and sterile alpha motif domain containing 1A | 0.74 |
| 1388710_at | NM_001107348 | Rreb1 | ras responsive element binding protein 1 | 0.74 |
| 1367535_at | NM_001107483 | Irf2bp1 | interferon regulatory factor 2 binding protein 1 | 0.74 |
| 1393662_at | XM_001056513 /// XM_341399 | Mapk8 | mitogen-activated protein kinase 8 | 0.74 |
| 1382363_at | NM_001108034 | Mpp5 | membrane protein, palmitoylated 5 (MAGUK p55 subfamily member 5) | 0.74 |
| 1383186_at | NM_001106759 | RGD1307749 | similar to RIKEN cDNA 1600013K19 | 0.74 |
| 1383359_at | NM_001108329 | Lnx2 | ligand of numb-protein X 2 | 0.74 |
| 1384237_at | NM_001109405 | Pex10 | peroxisomal biogenesis factor 10 | 0.74 |
| 1371934_at | NM_001127638 | Usp21 | ubiquitin specific peptidase 21 | 0.73 |
| 1392550_at | XM_001077448 /// XM_002726752 /// XM_002729635 /// XM_576044 | Dact1 | Dapper, antagonist of beta-catenin, homolog 1 (Xenopus laevis) | 0.73 |
| 1378218_at | NM_001134714 | Tcfcp2 | transcription factor CP2 | 0.73 |
| 1389657_at | NM_001108905 | Znf524 | zinc finger protein 524 | 0.73 |
| 1383282_at | NM_001107422 | Thap11 | THAP domain containing 11 | 0.73 |
| 1383535_at | NM_001108109 | Asb8 | ankyrin repeat and SOCS box-containing 8 | 0.73 |
| 1391461_at | NM_001106648 | RGD1306576 | similar to hypothetical protein | 0.73 |
| 1390995_at | NM_001002818 | Fkbpl | FK506 binding protein-like | 0.73 |
| 1384432_at | XM_002725276 /// XM_002728485 | Zscan12 | zinc finger and SCAN domain containing 12 | 0.73 |
| 1378049_at | NM_001191612 /// XM_001060148 /// XM_216345 | Zfp362 | Zinc finger protein 362 | 0.72 |
| 1380668_at | NM_198772 | MGC72974 | Hypothetical LOC316976 | 0.72 |
| 1391701_at | NM_001100570 | Myst3 | MYST histone acetyltransferase (monocytic leukemia) 3 | 0.72 |
| 1383245_at | NM_001108712 | Mbip | MAP3K12 binding inhibitory protein 1 | 0.72 |
| 1374693_at | NM_001014093 | Parp16 | poly (ADP-ribose) polymerase family, member 16 | 0.71 |
| 1369227_at | NM_017067 | Chm | choroideremia (Rab escort protein 1) | 0.71 |
| 1389261_at | XR_006091 /// XR_009409 | RGD1305508 | similar to hypothetical protein MGC23280 | 0.71 |
| 1367593_at | NM_013027 | Sepw1 | selenoprotein W, 1 | 0.71 |
| 1398710_at | NM_001024779 | Cyp2u1 | cytochrome P450, family 2, subfamily u, polypeptide 1 | 0.71 |
| 1389437_at | NM_001107262 | Sall2 | sal-like 2 (Drosophila) | 0.71 |
| 1383670_at | NM_001079943 | Zfp426 | zinc finger protein 426 | 0.70 |
| 1391702_at |  | Zfp446 | Zinc finger protein 446 | 0.70 |
| 1391817_at | NM_001106661 | RGD1304595 | similar to RIKEN cDNA 6330416G13 gene | 0.70 |
| 1397717_at | NM_001008367 | Isoc2b | isochorismatase domain containing 2b | 0.70 |
| 1398607_at | NM_001106888 | RGD1305680 | Similar to KIAA0240 | 0.70 |
| 1370469_at | NM_145683 | Ptpn7 | protein tyrosine phosphatase, non-receptor type 7 | 0.70 |
| 1379475_at | XM_001081289 /// XM_575241 | RGD1563120 | similar to RIKEN cDNA 2210009G21 | 0.70 |
| 1372248_at | NM_001106396 | Sesn1 | sestrin 1 | 0.70 |
| 1393583_at | NM_001109094 | Etaa1 | Ewing tumor-associated antigen 1 | 0.70 |
| 1372396_at | NM_001108755 | RGD1308026 | similar to 2310047B19Rik protein | 0.70 |
| 1372384_at | NM_001173371 /// XM_001076726 /// XM_343738 | Znf609 | zinc finger protein 609 | 0.70 |
| 1375799_at | XM_001079243 /// XM_002726782 | Tmem121 | transmembrane protein 121 | 0.70 |
| 1387393_at | NM_031639 | Dlg3 | discs, large homolog 3 (Drosophila) | 0.70 |
| 1379196_at | NM_001109128 | Fbxl8 | F-box and leucine-rich repeat protein 8 | 0.69 |
| 1371672_at | NM_199117 | Cbx7 | chromobox homolog 7 | 0.69 |
| 1395695_at | NM_001100970 | Aebp1 | AE binding protein 1 | 0.69 |
| 1385046_at | NM_001109435 | Fam55c | family with sequence similarity 55, member C | 0.69 |
| 1387982_at | NM_019178 | Tlr4 | toll-like receptor 4 | 0.69 |
| 1384377_at | NM_001108898 | Ddx28 | DEAD (Asp-Glu-Ala-Asp) box polypeptide 28 | 0.69 |
| 1393127_at | NM_001108328 | Zfp358 | zinc finger protein 358 | 0.69 |
| 1382993_at | NM_173837 | Bbc3 | Bcl-2 binding component 3 | 0.69 |
| 1379295_at | NM_001135767 | Gngt2 | guanine nucleotide binding protein (G protein), gamma transducing activity polypeptide 2 | 0.68 |
| 1385534_at | NM_053401 | Ngfrap1 | nerve growth factor receptor (TNFRSF16) associated protein 1 | 0.68 |
| 1374032_at | NM_001013117 | Phf12 | PHD finger protein 12 | 0.68 |
| 1384175_at | NM_001107692 | Efna4 | ephrin A4 | 0.68 |
| 1398585_at | NM_001108338 | Fbxo21 | F-box protein 21 | 0.68 |
| 1381208_at | NM_021840 | Hist3h2a | histone cluster 3, H2a | 0.68 |
| 1389774_at | NM_001107428 | Znf23 | zinc finger protein 23 (KOX 16) | 0.68 |
| 1383414_at | NM_001134571 | RGD1308117 | similar to 9930012K11Rik protein | 0.67 |
| 1389190_at | NM_001014247 | Lzts2 | leucine zipper, putative tumor suppressor 2 | 0.67 |
| 1377288_at | NM_001106177 | Hsf4 | heat shock transcription factor 4 | 0.67 |
| 1387429_at | NM_012776 | Adrbk1 | adrenergic, beta, receptor kinase 1 | 0.67 |
| 1376607_a_at | NM_001013883 | RGD1310414 | similar to hypothetical protein FLJ23263 | 0.67 |
| 1369723_at | NM_022296 | Xylt2 | xylosyltransferase II | 0.67 |
| 1392057_at | NM_001029926 | LOC500893 | similar to GLI-Kruppel family member GLI4 | 0.67 |
| 1368693_at | NM_024145 | Fgr | Gardner-Rasheed feline sarcoma viral (v-fgr) oncogene homolog | 0.67 |
| 1384084_at | NM_001134575 | RGD1308106 | LOC361719 | 0.67 |
| 1369203_at | NM_053738 | Wif1 | Wnt inhibitory factor 1 | 0.66 |
| 1386793_at | NM_001017512 | Zfp61 | zinc finger protein 61 | 0.66 |
| 1385556_at | XM_001069457 /// XM_342892 | RGD1308616 | similar to KIAA0467 protein | 0.66 |
| 1379766_at | NM_178097 | Sla | Src-like adaptor | 0.65 |
| 1390907_at | XM_001077082 /// XM_002726875 | LOC691170 | Similar to zinc finger protein 84 (HPF2) | 0.65 |
| 1376630_at | NM_001014020 /// NM_001106267 | Tarsl2 /// Tm2d3 | threonyl-tRNA synthetase-like 2 /// TM2 domain containing 3 | 0.65 |
| 1377748_at | NM_001126281 | LOC314600 | similar to zinc finger protein 422, related sequence 1 | 0.65 |
| 1388274_at | NM_001013163 | Bmyc | brain expressed myelocytomatosis oncogene | 0.64 |
| 1383252_at | NM_001106240 | Sars2 | seryl-tRNA synthetase 2, mitochondrial | 0.64 |
| 1373786_at | NM_001109425 | Znf703 | zinc finger protein 703 | 0.64 |
| 1381895_at | NM_001100708 | Nrf1 | nuclear respiratory factor 1 | 0.64 |
| 1398575_at | NM_001106657 | Zbtb5 | zinc finger and BTB domain containing 5 | 0.64 |
| 1390861_at | NM_001163062 | Zfp709l2 | zinc finger protein 709-like 2 | 0.63 |
| 1384756_at | NM_001105812 | Slc43a2 | solute carrier family 43, member 2 | 0.63 |
| 1383608_at | NM_001107303 | Arrdc2 | arrestin domain containing 2 | 0.62 |
| 1368156_at | NM_031662 | Camkk1 | calcium/calmodulin-dependent protein kinase kinase 1, alpha | 0.62 |
| 1370589_at | NM_153731 | Zfp709 | zinc finger protein 709 | 0.62 |
| 1390034_at | NM_001100680 | Ralgps2 | Ral GEF with PH domain and SH3 binding motif 2 | 0.62 |
| 1370984_at | NM_001106691 | Zfp46 | zinc finger protein 46 | 0.62 |
| 1383969_at | XR_086123 | LOC100362658 | zinc finger protein 658-like | 0.62 |
| 1375575_at | NM_001042354 /// NM_001042356 /// NM_021739 | Camk2b | calcium/calmodulin-dependent protein kinase II beta | 0.62 |
| 1392593_a_at | XM_001080668 /// XM_341852 | Prr12 | proline rich 12 | 0.62 |
| 1391338_at | XM_002725600 /// XM_574407 | Eid2b | EP300 interacting inhibitor of differentiation 2B | 0.62 |
| 1397836_at | NM_001107147 | Sirt4 | sirtuin (silent mating type information regulation 2 homolog) 4 (S. cerevisiae) | 0.62 |
| 1381153_at | NM_001107220 | Anapc4 | anaphase promoting complex subunit 4 | 0.61 |
| 1376525_at | NM_133602 | Khsrp | KH-type splicing regulatory protein | 0.61 |
| 1380581_at | NM_001109677 | LOC100125361 | zinc finger protein LOC100125361 | 0.60 |
| 1385351_at | NM_001162535 | RGD1305090 | similar to CD2-associated protein | 0.60 |
| 1391976_at | NM_001106739 | Six4 | SIX homeobox 4 | 0.60 |
| 1390928_at | XM_002726369 /// XM_002729319 | RGD1559612 | similar to tigger transposable element derived 2 | 0.59 |
| 1375988_at | XM_001056376 /// XM_002727022 /// XM_002727023 /// XM_002727024 /// XM_002729924 /// XM_002729925 | LOC680262 | hypothetical protein LOC680262 | 0.59 |
| 1388661_at | NM_001006960 | Mtp18 | mitochondrial protein 18 kDa | 0.59 |
| 1375531_at | XM_001055013 /// XM_216905 | Lrig3 | leucine-rich repeats and immunoglobulin-like domains 3 | 0.58 |
| 1384312_at | NM_001107331 | Irx1 | iroquois homeobox 1 | 0.58 |
| 1391819_at | NM_001108308 | RGD1307394 | similar to hypothetical protein ET | 0.58 |
| 1395794_at | NM_001034068 /// NM_001034069 /// NM_001034070 /// NM_001034071 /// NM_001034072 /// NM_001034073 /// NM_001034074 /// NM_001034075 /// NM_019131 | Tpm1 | tropomyosin 1, alpha | 0.58 |
| 1386721_at | NM_001107250 | Znf503 | zinc finger protein 503 | 0.57 |
| 1381798_at | NM_001001515 | Lmo7 | LIM domain 7 | 0.57 |
| 1380556_at | XM_001071167 /// XM_342829 | Fbxo10 | F-box protein 10 | 0.57 |
| 1383342_at | NM_001024354 | Lrrc14 | leucine rich repeat containing 14 | 0.57 |
| 1389002_at | NM_001039025 | Tln1 | talin 1 | 0.57 |
| 1398482_at | NM_001109422 | Bcl3 | B-cell CLL/lymphoma 3 | 0.56 |
| 1393175_at | XR_085713 /// XR_086122 | RGD1562094 | Similar to RIKEN cDNA 2810426N06 | 0.56 |
| 1392064_at | NM_001100531 | Dlx1 | distal-less homeobox 1 | 0.54 |
| 1388525_at | NM_001017453 | Pik3ip1 | phosphoinositide-3-kinase interacting protein 1 | 0.54 |
| 1379457_at | NM_001106779 | Nedd1 | neural precursor cell expressed, developmentally down-regulated 1 | 0.48 |
| 1378369_at | XM_001077448 /// XM_002726752 /// XM_002729635 /// XM_576044 | Dact1 | dapper, antagonist of beta-catenin, homolog 1 (Xenopus laevis) | 0.48 |
| 1393990_at | NM_001107250 | Znf503 | zinc finger protein 503 | 0.48 |
| 1368025_at | NM_080906 | Ddit4 | DNA-damage-inducible transcript 4 | 0.46 |
| 1369463_at | NM_013148 | Htr5a | 5-hydroxytryptamine (serotonin) receptor 5A | 0.45 |
| 1380530_at | XM_001072241 /// XM_002726984 | Maml2 | mastermind like 2 (Drosophila) | 0.40 |
| 1371131_a_at | NM_001008767 | Txnip | thioredoxin interacting protein | 0.34 |
| 1393083_at | NM_001108654 | Tox | Thymocyte selection-associated high mobility group box | 0.31 |

a Fold change greater than 1.0 represents increases, while less than 1.0 indicates decreases in stretching versus control group.
