# Supplementary material for: Gene Expression Changes under Cyclic Mechanical Stretching in Rat Retinal Glial (Müller) Cells
Source: PLoS One. 2013 May 27;8(5):e63467. doi: 10.1371/journal.pone.0063467 (PMC3664568; doi:10.1371/journal.pone.0063467)
Supplement: Table S2 — Up- and downregulated Genes in Müller cells after stretching for 24 h. (DOC) [file pone.0063467.s002.doc]

| Table S2. Up- and downregulated Genes (p<0.05) in Müller cells after stretching for 24 h | | | | |
| --- | --- | --- | --- | --- |
| **ProbeSetID** | **Accession#** | **Gene** | **GeneTitle** | **fold^a^** |
| 1382351_at | NM_001106637 | Gem | GTP binding protein (gene overexpressed in skeletal muscle) | 2.70 |
| 1384035_at | XM_001063122 /// XM_002724859 | LOC685277 | Similar to liver-specific bHLH-Zip transcription factor | 2.66 |
| 1386969_at | NM_053346 | Nrn1 | neuritin 1 | 2.60 |
| 1389735_at | NM_001191721 /// XM_001061436 /// XM_228473 | Rps6ka6 | Ribosomal protein S6 kinase polypeptide 6 | 2.60 |
| 1396206_at | NM_001109344 | RGD1562846 | similar to Docking protein 5 (Downstream of tyrosine kinase 5) (Protein dok-5) | 2.55 |
| 1370315_a_at | NM_019176 | Stmn4 | stathmin-like 4 | 2.40 |
| 1371913_at | NM_053802 | Tgfbi | transforming growth factor, beta induced | 2.33 |
| 1390943_at | NM_001014193 | RGD1359529 | similar to chromosome 1 open reading frame 63 | 2.28 |
| 1383205_at | NM_001107464 | Dact2 | dapper, antagonist of beta-catenin, homolog 2 (Xenopus laevis) | 2.28 |
| 1393563_at | NM_001167840 /// NM_012968 | Il1rap | interleukin 1 receptor accessory protein | 2.27 |
| 1380681_at | NM_001134986 | Rnf180 | ring finger protein 180 | 2.25 |
| 1375684_at | NM_031522 | Neu1 | sialidase 1 (lysosomal sialidase) | 2.20 |
| 1369193_at | NM_130812 | Cdkn2b | cyclin-dependent kinase inhibitor 2B (p15, inhibits CDK4) | 2.19 |
| 1384252_at | NM_001106909 | RGD1309095 | similar to hypothetical protein BC015148 | 2.15 |
| 1368924_at | NM_017094 | Ghr | growth hormone receptor | 2.06 |
| 1380474_at | NM_001106047 | Loxl2 | Lysyl oxidase-like 2 | 2.05 |
| 1373368_at | NM_001127640 | PCOLCE2 | procollagen C-endopeptidase enhancer 2 | 1.98 |
| 1386120_at | XM_001056121 /// XM_001069739 | LOC689147 | Hypothetical protein LOC689147 | 1.97 |
| 1377970_at | XM_001068789 /// XM_215491 | Parp8 | poly (ADP-ribose) polymerase family, member 8 | 1.97 |
| 1385853_at | NM_001170568 /// XM_001079067 /// XM_234156 | Baz1a | bromodomain adjacent to zinc finger domain, 1A | 1.97 |
| 1388721_at | NM_053612 | Hspb8 | heat shock protein B8 | 1.95 |
| 1393191_at | XM_001062503 /// XM_575920 | Nkain1 | Na+/K+ transporting ATPase interacting 1 | 1.92 |
| 1390397_at | NM_001025020 | Tmem90b | transmembrane protein 90B | 1.90 |
| 1369012_at | NM_017128 | Inhba | inhibin beta-A | 1.89 |
| 1369871_at | NM_017123 | Areg | amphiregulin | 1.89 |
| 1368947_at | NM_024127 | Gadd45a | growth arrest and DNA-damage-inducible, alpha | 1.88 |
| 1372827_at | NM_001004279 | Ppid | peptidylprolyl isomerase D (cyclophilin D) | 1.87 |
| 1392650_at | NM_053824 | Csnk2a1 | casein kinase 2, alpha 1 polypeptide | 1.85 |
| 1383096_at | XM_002729883 | LOC100363366 | amyloid beta (A4) precursor-like protein 2-like | 1.84 |
| 1370541_at | NM_147210 | Nr1d2 | nuclear receptor subfamily 1, group D, member 2 | 1.83 |
| 1397964_at | NM_001107637 | Sec63 | SEC63 homolog (S. cerevisiae) | 1.82 |
| 1393947_at | NM_001047880 | Slc25a15 | Solute carrier family 25 (mitochondrial carrier; ornithine transporter) member 15 | 1.81 |
| 1392406_at | NM_001106679 | Ipp | intracisternal A particle-promoted polypeptide | 1.80 |
| 1369737_at | NM_001110860 /// NM_017334 | Crem | cAMP responsive element modulator | 1.79 |
| 1391083_at | NM_001107297 | Arhgap22 | Rho GTPase activating protein 22 | 1.78 |
| 1385808_at | NM_001012211 | Phf7 | PHD finger protein 7 | 1.78 |
| 1394943_at | NM_001106279 | RGD1565584 | Similar to tyrosine kinase-associated leucine zipper protein LAZipII | 1.77 |
| 1390886_at | NM_001107667 | Ccdc39 | Coiled-coil domain containing 39 | 1.77 |
| 1387290_at | NM_130742 | Galnt10 | UDP-N-acetyl-alpha-D-galactosamine:polypeptide N-acetylgalactosaminyltransferase 10 (GalNAc-T10) | 1.76 |
| 1392979_at | NM_001004208 | Cacybp | calcyclin binding protein | 1.76 |
| 1370357_at | NM_172066 | Slc30a4 | solute carrier family 30 (zinc transporter), member 4 | 1.74 |
| 1382263_at | NM_001134708 | Odf2l | outer dense fiber of sperm tails 2-like | 1.73 |
| 1372897_at | NM_001142915 /// NM_175869 | Plod2 | procollagen lysine, 2-oxoglutarate 5-dioxygenase 2 | 1.71 |
| 1389521_at | NM_001047085 | Ivns1abp | influenza virus NS1A binding protein | 1.71 |
| 1387824_at | NM_020092 | Sfrs12 | splicing factor, arginine/serine-rich 12 | 1.70 |
| 1373884_at | XM_001073589 /// XM_214331 | Klhl2 | kelch-like 2, Mayven (Drosophila) | 1.69 |
| 1369452_a_at | NM_053554 | Picalm | phosphatidylinositol binding clathrin assembly protein | 1.67 |
| 1367841_a_at | NM_134385 | Prl8a9 | prolactin family 8, subfamily a, member 9 | 1.67 |
| 1377902_a_at | NM_001106617 | Rad52 | RAD52 homolog (S. cerevisiae) | 1.67 |
| 1373169_at | NM_001134744 | Agpat5 | 1-acylglycerol-3-phosphate O-acyltransferase 5 (lysophosphatidic acid acyltransferase, epsilon) | 1.65 |
| 1393317_at | NM_001170546 /// NM_001170547 /// NM_001170548 /// XM_001078607 /// XM_575638 | Thumpd3 | THUMP domain containing 3 | 1.62 |
| 1387875_at | NM_013081 | Ptk2 | PTK2 protein tyrosine kinase 2 | 1.62 |
| 1392083_at | NM_001130540 /// XM_001065900 /// XM_216920 | Ext1 | exostoses (multiple) 1 | 1.62 |
| 1393306_at | NM_022683 | Cul5 | Cullin 5 | 1.62 |
| 1390107_at | NM_001108492 | Sytl2 | synaptotagmin-like 2 | 1.61 |
| 1387116_at | NM_012699 | Dnajb9 | DnaJ (Hsp40) homolog, subfamily B, member 9 | 1.61 |
| 1385741_at | NM_001017447 | RGD1305235 | similar to RIKEN cDNA 1700052N19 | 1.61 |
| 1376285_at | NM_001013171 | Gulp1 | GULP, engulfment adaptor PTB domain containing 1 | 1.61 |
| 1392079_at | NM_001001801 | Akap7 | A kinase (PRKA) anchor protein 7 | 1.60 |
| 1377772_at | NM_023020 | Tmeff1 | transmembrane protein with EGF-like and two follistatin-like domains 1 | 1.60 |
| 1391454_at | NM_001106279 | Crebzf | CREB/ATF bZIP transcription factor | 1.60 |
| 1388771_at | NM_001105900 | Cggbp1 | CGG triplet repeat binding protein 1 | 1.59 |
| 1380646_at | NM_001109506 | Elmod2 | ELMO/CED-12 domain containing 2 | 1.59 |
| 1378936_at | NM_001131014 | Cstf2 | cleavage stimulation factor, 3' pre-RNA subunit 2 | 1.59 |
| 1398311_a_at | NM_053795 | Kidins220 | kinase D-interacting substrate 220 | 1.59 |
| 1396233_at | NM_001047917 | Arsk | Arylsulfatase family, member K | 1.59 |
| 1387808_at | NM_031341 | Slc7a7 | solute carrier family 7 (cationic amino acid transporter, y+ system), member 7 | 1.59 |
| 1387841_at | NM_022204 | Exoc5 | exocyst complex component 5 | 1.58 |
| 1379729_at | NM_001106785 | Cpsf6 | cleavage and polyadenylation specific factor 6 | 1.57 |
| 1391578_at | XR_005468 /// XR_009637 | RGD1307526 | similar to modulator of estrogen induced transcription | 1.57 |
| 1384268_at | XM_001072068 /// XM_237211 | Ccnyl1 | cyclin Y-like 1 | 1.57 |
| 1380682_at | NM_001191626 /// XM_001068105 /// XM_218846 | Mex3b | mex3 homolog B (C. elegans) | 1.56 |
| 1367815_at | NM_130746 | Slc5a6 | solute carrier family 5 (sodium-dependent vitamin transporter), member 6 | 1.56 |
| 1370112_at | NM_031606 | Pten | phosphatase and tensin homolog | 1.56 |
| 1372847_at | NM_001047914 | Acn9 | ACN9 homolog (S. cerevisiae) | 1.56 |
| 1376010_at | NM_001011923 | Prpf4b | PRP4 pre-mRNA processing factor 4 homolog B (yeast) | 1.55 |
| 1376883_at | NM_001007642 | Phospho2 | phosphatase, orphan 2 | 1.55 |
| 1377187_at | XM_001053414 /// XM_232819 | Rbm12b | RNA binding motif protein 12B | 1.54 |
| 1387408_at | NM_134457 | Siah2 | seven in absentia 2 | 1.54 |
| 1380726_at | NM_001014008 | Aspn | Asporin | 1.53 |
| 1385035_at | NM_001166576 | Usp12 | ubiquitin specific peptidase 12 | 1.53 |
| 1382315_at | NM_001044284 | Tsc22d4 | TSC22 domain family, member 4 | 1.53 |
| 1382784_at | NM_001135119 | Angel2 | angel homolog 2 (Drosophila) | 1.53 |
| 1383354_a_at | NM_001108023 | Fbxo33 | F-box protein 33 | 1.53 |
| 1387374_at | NM_013176 | Tcf12 | transcription factor 12 | 1.52 |
| 1382434_at | NM_199394 | Entpd5 | ectonucleoside triphosphate diphosphohydrolase 5 | 1.52 |
| 1388607_at | NM_001107061 | Smurf2 | SMAD specific E3 ubiquitin protein ligase 2 | 1.51 |
| 1389961_at | NM_001013135 | Sdccag3 | serologically defined colon cancer antigen 3 | 1.51 |
| 1375533_at | NM_001015004 | Vgll4 | vestigial like 4 (Drosophila) | 1.50 |
| 1389459_at | NM_001107356 | RGD1306613 | similar to RIKEN cDNA 1600012F09 | 1.50 |
| 1369443_at | NM_133569 | Angptl2 | angiopoietin-like 2 | 1.50 |
| 1371038_at | NM_012831 | Cebpg | CCAAT/enhancer binding protein (C/EBP), gamma | 1.50 |
| 1389533_at | XM_001062865 /// XM_001073286 | Fbln2 | fibulin 2 | 1.50 |
| 1389204_at | XM_001068906 /// XM_341552 | RGD1564456 | similar to chromosome 10 open reading frame 18 | 1.49 |
| 1373977_at | NM_001107730 | Kif5c | Kinesin family member 5C | 1.49 |
| 1389152_at | NM_199104 | Zfyve27 | zinc finger, FYVE domain containing 27 | 1.49 |
| 1379665_at | NM_001106406 | Ppwd1 | peptidylprolyl isomerase domain and WD repeat containing 1 | 1.49 |
| 1383160_at | NM_001108128 | Chordc1 | cysteine and histidine-rich domain (CHORD)-containing 1 | 1.49 |
| 1389381_at | NM_175843 /// NM_181550 | Sqstm1 | sequestosome 1 | 1.48 |
| 1387142_at | NM_017141 | Polb | polymerase (DNA directed), beta | 1.48 |
| 1372675_at | NM_001008288 | RGD1306954 | similar to RIKEN cDNA 1110004E09 | 1.48 |
| 1373445_at | NM_001108408 | Nol8 | nucleolar protein 8 | 1.48 |
| 1387750_at | NM_053530 | Twist1 | twist homolog 1 (Drosophila) | 1.48 |
| 1392514_at | NM_001106391 | Bxdc1 | brix domain containing 1 | 1.48 |
| 1380938_at | NM_199463 | Dstyk | dual serine/threonine and tyrosine protein kinase | 1.47 |
| 1374232_at | NM_133399 /// XM_001059350 /// XM_001063573 | Pik3ca | phosphoinositide-3-kinase, catalytic, alpha polypeptide | 1.47 |
| 1384005_at | NM_001011914 | Dr1 | down-regulator of transcription 1 | 1.47 |
| 1379282_at | NM_001024761 | Lrrfip2 | leucine rich repeat (in FLII) interacting protein 2 | 1.47 |
| 1379287_at | NM_001017380 /// XM_002728578 /// XR_085832 /// XR_086249 | Cyld /// LOC100362727 /// RGD1565647 | cylindromatosis (turban tumor syndrome) /// ubiquitin carboxyl-terminal hydrolase CYLD /// similar to cylindromatosis (turban tumor syndrome) | 1.46 |
| 1394473_at | NM_001108743 | Elk3 | ELK3, member of ETS oncogene family | 1.46 |
| 1374401_at | NM_001106135 | Snx2 | sorting nexin 2 | 1.46 |
| 1382466_at | NM_001106107 | Snrnp48 | small nuclear ribonucleoprotein 48k (U11/U12) | 1.46 |
| 1392985_at | NM_001013108 | Arih1 | ariadne ubiquitin-conjugating enzyme E2 binding protein homolog 1 (Drosophila) | 1.46 |
| 1368326_at | NM_031599 | Eif2ak3 | eukaryotic translation initiation factor 2 alpha kinase 3 | 1.45 |
| 1383106_at | NM_001108417 | Cul2 | cullin 2 | 1.45 |
| 1392207_at | NM_001024269 | Luc7l | LUC7-like (S. cerevisiae) | 1.45 |
| 1390500_at | XM_001055784 /// XM_223651 | Aftph | aftiphilin | 1.44 |
| 1368177_at | NM_057107 | Acsl3 | acyl-CoA synthetase long-chain family member 3 | 1.44 |
| 1376050_at | NM_001106365 | Taf5 | TAF5 RNA polymerase II, TATA box binding protein (TBP)-associated factor | 1.43 |
| 1384937_at | NM_001191664 /// XM_001072631 /// XM_221920 | Rbak | RB-associated KRAB zinc finger | 1.43 |
| 1372294_at | XM_001053103 /// XM_002726729 | Pxdn | peroxidasin homolog (Drosophila) | 1.43 |
| 1368172_a_at | NM_017061 | Lox | lysyl oxidase | 1.43 |
| 1387430_at | NM_031694 | Hsf2 | heat shock transcription factor 2 | 1.43 |
| 1367860_a_at | NM_031056 | Mmp14 | matrix metallopeptidase 14 (membrane-inserted) | 1.43 |
| 1373012_at | NM_001034912 | Fam134b | Family with sequence similarity 134, member B | 1.42 |
| 1388758_at | NM_017107 | Ogt | O-linked N-acetylglucosamine (GlcNAc) transferase (UDP-N-acetylglucosamine:polypeptide-N-acetylglucosaminyl transferase) | 1.42 |
| 1387784_at | NM_053925 | Psmd10 | proteasome (prosome, macropain) 26S subunit, non-ATPase, 10 | 1.42 |
| 1381364_at | XM_001070389 /// XM_219500 | Tnpo1 | transportin 1 | 1.42 |
| 1375527_at | NM_022933 | Chd8 | chromodomain helicase DNA binding protein 8 | 1.42 |
| 1369785_at | NM_057198 | Ppat | phosphoribosyl pyrophosphate amidotransferase | 1.42 |
| 1393568_at | NM_001025421 | Cugbp1 | CUG triplet repeat, RNA binding protein 1 | 1.42 |
| 1382144_at | NM_001037183 | Mrpl47 | mitochondrial ribosomal protein L47 | 1.41 |
| 1374976_a_at | NM_031118 | Soat1 | Sterol O-acyltransferase 1 | 1.41 |
| 1397556_at | NM_001105881 | Nat13 | N-acetyltransferase 13 | 1.41 |
| 1389672_at | NM_001108753 | Uhrf1bp1l | UHRF1 binding protein 1-like | 1.41 |
| 1395199_at | NM_001077670 | Eif3j | eukaryotic translation initiation factor 3, subunit J | 1.41 |
| 1396170_at | NM_053766 | Wbp4 | WW domain binding protein 4 (formin binding protein 21) | 1.40 |
| 1372878_at | XM_001056210 /// XM_345169 | Zfr | zinc finger RNA binding protein | 1.40 |
| 1374027_at | XM_001060246 /// XM_002725277 /// XM_002728484 /// XM_577579 | Zfp187 | zinc finger protein 187 | 1.40 |
| 1392505_at | NM_001083624 | Pds5a | PDS5, regulator of cohesion maintenance, homolog A (S. cerevisiae) | 1.40 |
| 1367688_at | NM_031725 | Scamp4 | secretory carrier membrane protein 4 | 1.40 |
| 1390249_at | NM_001025011 | RGD1305464 | similar to human chromosome 15 open reading frame 39 | 1.40 |
| 1374025_at | NM_001013224 | Nmnat3 | nicotinamide nucleotide adenylyltransferase 3 | 1.40 |
| 1375872_at | NM_001108667 | Fktn | Fukutin | 1.40 |
| 1370360_at | NM_138865 | RGD621352 | similar to RIKEN cDNA 1500031L02 | 1.40 |
| 1389191_at | NM_001106092 | Vps36 | vacuolar protein sorting 36 homolog (S. cerevisiae) | 1.40 |
| 1384413_at | NM_001191927 /// XM_001066912 /// XM_573283 | Slc35a5 | Solute carrier family 35, member A5 | 1.39 |
| 1389510_at | NM_001011911 | Lyar | Ly1 antibody reactive homolog (mouse) | 1.39 |
| 1392054_at | XM_001059164 /// XM_240329 | Pbrm1 | polybromo 1 | 1.39 |
| 1368088_at | NM_080885 | Cdk5 | cyclin-dependent kinase 5 | 1.39 |
| 1395071_at | NM_001108953 | Zbtb6 | zinc finger and BTB domain containing 6 | 1.39 |
| 1392468_at | NM_001134503 | Camsap1l1 | calmodulin regulated spectrin-associated protein 1-like 1 | 1.39 |
| 1393799_at | NM_022207 | Unc5b | Unc-5 homolog B (C. elegans) | 1.39 |
| 1391091_at | NM_001017380 /// XM_002728590 /// XR_085832 /// XR_086249 | Cyld /// LOC100360670 /// RGD1565647 | cylindromatosis (turban tumor syndrome) /// probable ubiquitin carboxyl-terminal hydrolase CYLD-like /// similar to cylindromatosis (turban tumor syndrome) | 1.39 |
| 1386078_at | NM_001107924 | Slc35a1 | solute carrier family 35 (CMP-sialic acid transporter), member A1 | 1.38 |
| 1396123_at | NM_001109054 | Tctex1d2 | Tctex1 domain containing 2 | 1.38 |
| 1371232_a_at | NM_001170558 /// NM_001170559 /// NM_001170560 /// NM_053663 /// XM_001058357 /// XM_215451 | Vcan | versican | 1.38 |
| 1383447_at | NM_001107082 | Etv5 | ets variant 5 | 1.38 |
| 1380854_at | NM_053549 | Vegfb | vascular endothelial growth factor B | 1.38 |
| 1383477_at | NM_001012149 | Uchl5 | ubiquitin carboxyl-terminal hydrolase L5 | 1.38 |
| 1372067_at | NM_001024800 | Tmx1 | thioredoxin-related transmembrane protein 1 | 1.38 |
| 1377338_at | NM_001106419 | Rad1 | RAD1 homolog (S. pombe) | 1.37 |
| 1367487_at | NM_001009539 | B4galt3 | UDP-Gal:betaGlcNAc beta 1,4-galactosyltransferase, polypeptide 3 | 1.37 |
| 1367515_at | NM_001107313 | Cnot7 | CCR4-NOT transcription complex, subunit 7 | 1.37 |
| 1373214_at | NM_001108217 | Kdelc1 | KDEL (Lys-Asp-Glu-Leu) containing 1 | 1.37 |
| 1375960_at | NM_001010956 /// NM_001033931 | Gmcl1 | germ cell-less homolog 1 (Drosophila) | 1.37 |
| 1370826_at | NM_053561 | Nap1l1 | nucleosome assembly protein 1-like 1 | 1.37 |
| 1374586_at | XM_001069431 /// XM_002725902 | Arl15 | ADP-ribosylation factor-like 15 | 1.37 |
| 1383336_at | NM_001109023 | Pnn | pinin, desmosome associated protein | 1.37 |
| 1372543_at | NM_001108818 | RGD1562502 | similar to RIKEN cDNA 2610029G23 | 1.37 |
| 1399118_at | NM_001107048 | Msl1 | male-specific lethal 1 homolog (Drosophila) | 1.37 |
| 1383528_at | XM_001056752 /// XM_234528 | RGD1563945 | Similar to mKIAA0215 protein | 1.37 |
| 1373885_at | NM_001106797 | Cbx5 | chromobox homolog 5 (HP1 alpha homolog, Drosophila) | 1.37 |
| 1380663_at | NM_001107552 | Fbxl19 | F-box and leucine-rich repeat protein 19 | 1.37 |
| 1375915_at | NM_001106843 | Irak1bp1 | interleukin-1 receptor-associated kinase 1 binding protein 1 | 1.36 |
| 1388194_at | NM_031025 | Dlat | dihydrolipoamide S-acetyltransferase | 1.36 |
| 1384435_at | NM_001107903 | Tmem68 | transmembrane protein 68 | 1.36 |
| 1399033_at | NM_001013191 | Cbfb | core-binding factor, beta subunit | 1.36 |
| 1385349_at | XM_001067602 /// XM_002725933 | Cetn4 | centrin 4 | 1.36 |
| 1393105_at | NM_001137646 | Zfp259 | zinc finger protein 259 | 1.36 |
| 1396034_at | NM_001012056 | Ces7 | carboxylesterase 7 | 1.36 |
| 1389358_at | NM_001109376 | LOC679692 | Similar to lysophosphatidylglycerol acyltransferase 1 | 1.36 |
| 1375542_at | NM_001005889 | Rdx | Radixin | 1.36 |
| 1392654_at | NM_001025723 | Mtrf1l | mitochondrial translational release factor 1-like | 1.36 |
| 1373245_at | NM_001135009 | Col4a1 | collagen, type IV, alpha 1 | 1.36 |
| 1377998_at | NM_001037095 | Cpox | coproporphyrinogen oxidase | 1.36 |
| 1373015_at | XM_001067720 | LOC683844 | similar to RING finger protein 11 (NEDD4 WW domain-binding protein 2) (Sid 1669) | 1.36 |
| 1384187_at | NM_001127531 | Ap1s2 | adaptor-related protein complex 1, sigma 2 subunit | 1.36 |
| 1396317_at | NM_001108770 | Ccpg1 | cell cycle progression 1 | 1.35 |
| 1375686_at | NM_175707 | Ppil3 | peptidylprolyl isomerase (cyclophilin)-like 3 | 1.35 |
| 1372997_at | NM_001134997 | LOC687713 | similar to Protein C22orf13 homolog | 1.35 |
| 1373742_at | NM_001009660 | Spsb2 | splA/ryanodine receptor domain and SOCS box containing 2 | 1.35 |
| 1368426_at | NM_031987 | Crot | carnitine O-octanoyltransferase | 1.35 |
| 1376157_at | NM_001107213 | Uba6 | Ubiquitin-like modifier activating enzyme 6 | 1.35 |
| 1371551_at | NM_001107017 | Traf4 | Tnf receptor associated factor 4 | 1.35 |
| 1382396_at | NM_001034911 | Ubfd1 | ubiquitin family domain containing 1 | 1.35 |
| 1383830_a_at | NM_172023 | Osbpl1a | oxysterol binding protein-like 1A | 1.35 |
| 1371785_at | NM_181086 | Tnfrsf12a | tumor necrosis factor receptor superfamily, member 12a | 1.34 |
| 1385809_at | XR_085786 /// XR_086207 | Fastkd5 | FAST kinase domains 5 | 1.34 |
| 1398805_at | NM_134394 | Atg3 | ATG3 autophagy related 3 homolog (S. cerevisiae) | 1.34 |
| 1390214_a_at | NM_001106568 | Ciz1 | CDKN1A interacting zinc finger protein 1 | 1.34 |
| 1374642_at | NM_001012093 | Zfp64 | zinc finger protein 64 | 1.34 |
| 1372812_at | NM_001168527 | Secisbp2l | SECIS binding protein 2-like | 1.34 |
| 1395152_at | NM_001014002 | Mak16 | MAK16 homolog (S. cerevisiae) | 1.34 |
| 1389682_at | NM_001025016 | Chac2 | ChaC, cation transport regulator homolog 2 (E. coli) | 1.34 |
| 1368843_at | NM_053682 | Yme1l1 | YME1-like 1 (S. cerevisiae) | 1.34 |
| 1373071_at | NM_001106245 | Lin37 | lin-37 homolog (C. elegans) | 1.34 |
| 1373632_at | NM_001012463 /// NM_001037310 /// NM_184048 | Taf9 | TAF9 RNA polymerase II, TATA box binding protein (TBP)-associated factor | 1.34 |
| 1367960_at | NM_019186 | Arl4a | ADP-ribosylation factor-like 4A | 1.34 |
| 1389526_at | NM_001130564 | Efr3a | EFR3 homolog A (S. cerevisiae) | 1.33 |
| 1372214_at | NM_001047863 | Mrps33 | mitochondrial ribosomal protein S33 | 1.33 |
| 1376628_at | NM_001107930 | Zfp189 | zinc finger protein 189 | 1.33 |
| 1382765_at | XM_001080091 /// XM_002726164 /// XM_002726165 | LOC691918 | similar to Centrosomal protein of 27 kDa (Cep27 protein) | 1.33 |
| 1373820_at | NM_001025126 | Rfwd2 | ring finger and WD repeat domain 2 | 1.33 |
| 1392641_at | NM_001109645 | LOC691543 | Hypothetical protein LOC691543 | 1.33 |
| 1373507_at | NM_001077635 | Acbd5 | Acyl-Coenzyme A binding domain containing 5 | 1.33 |
| 1372710_at | NM_019251 | Bet1 | blocked early in transport 1 homolog (S. cerevisiae) | 1.33 |
| 1372283_at | NM_001106733 | Trappc6b | trafficking protein particle complex 6B | 1.33 |
| 1398937_at | NM_001191597 /// XM_001054651 /// XM_214053 | Dhx15 | DEAH (Asp-Glu-Ala-His) box polypeptide 15 | 1.33 |
| 1378036_at | NM_001107668 | Dcun1d1 | DCN1, defective in cullin neddylation 1, domain containing 1 (S. cerevisiae) | 1.33 |
| 1382228_at | XM_002729052 | LOC100359945 | rCG41562-like | 1.32 |
| 1375431_at | NM_001108219 | RGD1306941 | similar to CG31122-PA | 1.32 |
| 1382045_at | XM_001078627 /// XM_002726886 /// XM_002729785 /// XM_345825 | Tbc1d15 | TBC1 domain family, member 15 | 1.32 |
| 1392144_at | NM_001106393 | Nt5dc1 | 5'-nucleotidase domain containing 1 | 1.32 |
| 1369981_at | NM_031624 | Igbp1 | immunoglobulin (CD79A) binding protein 1 | 1.32 |
| 1372969_at | NM_001100568 | Fbxl3 | F-box and leucine-rich repeat protein 3 | 1.32 |
| 1393290_at | NM_001014035 | Mef2a | myocyte enhancer factor 2a | 1.32 |
| 1368565_at | NM_019225 | Slc1a3 | solute carrier family 1 (glial high affinity glutamate transporter), member 3 | 1.32 |
| 1376201_at | XR_085570 /// XR_085968 | Mett10d | methyltransferase 10 domain containing | 1.32 |
| 1376641_at | NM_001047850 | Thoc1 | THO complex 1 | 1.32 |
| 1387201_at | NM_053588 | Rnf138 | ring finger protein 138 | 1.32 |
| 1388573_at | NM_001108718 | Alkbh | AlkB, alkylation repair homolog (E. coli) | 1.32 |
| 1383375_at | NM_001024900 | Tmem55a | transmembrane protein 55A | 1.31 |
| 1374409_at | NM_001107575 | Taf6l | TAF6-like RNA polymerase II, p300/CBP-associated factor (PCAF)-associated factor | 1.31 |
| 1388896_at | NM_001191094 /// XM_001068666 /// XM_001080019 | Usp33 | ubiquitin specific peptidase 33 | 1.31 |
| 1380116_at | XM_001057462 /// XM_002726714 /// XM_237502 | RGD1562291 | similar to Human T-cell leukemia virus enhancer factor (Forkhead box protein N2) | 1.31 |
| 1377955_at | NM_001170403 | Orai2 | ORAI calcium release-activated calcium modulator 2 | 1.31 |
| 1399050_at | NM_001105975 | Adss | adenylosuccinate synthase | 1.31 |
| 1384149_at | NM_001108700 | Ccdc75 | coiled-coil domain containing 75 | 1.31 |
| 1393085_at | NM_001009714 | Mitd1 | MIT, microtubule interacting and transport, domain containing 1 | 1.31 |
| 1382146_at | NM_001100672 | Tspan6 | tetraspanin 6 | 1.31 |
| 1393915_at | NM_001012189 | Lpcat3 | lysophosphatidylcholine acyltransferase 3 | 1.31 |
| 1382643_at | NM_022289 | Snx16 | sorting nexin 16 | 1.30 |
| 1384347_at | NM_001108065 | Shc2 | SHC (Src homology 2 domain containing) transforming protein 2 | 1.30 |
| 1392548_at | NM_001047959 /// XM_001062863 /// XM_342453 | Cwc22 /// LOC500684 | CWC22 spliceosome-associated protein homolog (S. cerevisiae) /// hypothetical protein LOC500684 | 1.30 |
| 1374404_at | NM_021835 | Jun | Jun oncogene | 1.30 |
| 1392997_at | NM_001106613 | Ppp4r2 | protein phosphatase 4, regulatory subunit 2 | 1.30 |
| 1370348_at | NM_012867 | Ninj1 | ninjurin 1 | 1.30 |
| 1388141_at | NM_001191842 /// XM_001061358 /// XM_342168 | Cetn3 | centrin, EF-hand protein, 3 (CDC31 homolog, yeast) | 1.30 |
| 1379335_at | NM_001108937 | Paip1 | poly(A) binding protein interacting protein 1 | 1.30 |
| 1369955_at | NM_134452 | Col5a1 | Collagen, type V, alpha 1 | 1.30 |
| 1373380_at | NM_001010963 | Zc3h15 | zinc finger CCCH-type containing 15 | 1.30 |
| 1374537_at | NM_001106268 | Chsy1 | chondroitin sulfate synthase 1 | 1.30 |
| 1387732_at | NM_053499 | Mterf | mitochondrial transcription termination factor | 1.30 |
| 1384255_at | NM_001107384 | Napg | N-ethylmaleimide-sensitive factor attachment protein, gamma | 1.30 |
| 1392453_at | NM_053363 | Clcn3 | chloride channel 3 | 1.30 |
| 1372311_at | NM_001134995 | Gnpda1 | glucosamine-6-phosphate deaminase 1 | 1.30 |
| 1385090_at | NM_001024778 | Rad17 | RAD17 homolog (S. pombe) | 1.30 |
| 1394095_at | NM_001025012 | Gin1 | gypsy retrotransposon integrase 1 | 1.30 |
| 1370044_at | NM_080895 | Faim | Fas apoptotic inhibitory molecule | 1.29 |
| 1393453_at | NM_001097581 | Sav1 | salvador homolog 1 (Drosophila) | 1.29 |
| 1394318_at | NM_031660 | Arpp19 | cAMP-regulated phosphoprotein 19 | 1.29 |
| 1398993_at | NM_001013207 | Rbm39 | RNA binding motif protein 39 | 1.29 |
| 1369008_a_at | NM_053573 | Olfm1 | olfactomedin 1 | 1.29 |
| 1372339_at | NM_001013240 | Cdc26 | cell division cycle 26 | 1.29 |
| 1371929_at | NM_001034112 | Mlx | MAX-like protein X | 1.29 |
| 1373000_at | NM_001108243 | Srpx2 | sushi-repeat-containing protein, X-linked 2 | 1.29 |
| 1395654_at | NM_001108382 | Ccdc25 | coiled-coil domain containing 25 | 1.29 |
| 1398794_at | NM_022593 | Tceb1 | transcription elongation factor B (SIII), polypeptide 1 | 1.29 |
| 1379186_at | NM_212519 | Tomm70a | translocase of outer mitochondrial membrane 70 homolog A (S. cerevisiae) | 1.28 |
| 1388651_at | NM_001007618 | Rchy1 | ring finger and CHY zinc finger domain containing 1 | 1.28 |
| 1376161_at | NM_001107238 | Tmed4 | transmembrane emp24 protein transport domain containing 4 | 1.28 |
| 1377827_at | NM_001005536 | Srfbp1 | serum response factor binding protein 1 | 1.28 |
| 1398452_at | NM_001109099 | RGD1559923 | similar to chromosome 14 open reading frame 35 | 1.28 |
| 1381972_at | NM_001015003 | Crbn | cereblon | 1.28 |
| 1379525_at | NM_001014258 | Crls1 | cardiolipin synthase 1 | 1.28 |
| 1383108_at | NM_001107716 | Taf13 | TAF13 RNA polymerase II, TATA box binding protein (TBP)-associated factor | 1.28 |
| 1373154_at | XR_007629 /// XR_086285 | RGD1563583 | Similar to mKIAA0998 protein | 1.28 |
| 1374459_at | NM_001100710 | Alg2 | asparagine-linked glycosylation 2, alpha-1,3-mannosyltransferase homolog (S. cerevisiae) | 1.28 |
| 1380619_at | NM_001108822 | RGD1305537 | similar to RIKEN cDNA 3110001I22 | 1.28 |
| 1379615_at | NM_001109257 | Klhdc5 | kelch domain containing 5 | 1.27 |
| 1390895_at | NM_001106950 | LOC302495 | hypothetical LOC302495 | 1.27 |
| 1390688_at | NM_001013198 | Ddx50 | DEAD (Asp-Glu-Ala-Asp) box polypeptide 50 | 1.27 |
| 1371853_at | NM_001106782 | Mrpl42 | mitochondrial ribosomal protein L42 | 1.27 |
| 1391928_at | NM_001107631 | Gopc | golgi associated PDZ and coiled-coil motif containing | 1.27 |
| 1388977_at | NM_001007699 | Cept1 | choline/ethanolamine phosphotransferase 1 | 1.27 |
| 1390189_at | XM_001077681 /// XM_216701 | Zfp277 | zinc finger protein 277 | 1.27 |
| 1367485_at | NM_001025735 | Tcea1 | transcription elongation factor A (SII) 1 | 1.27 |
| 1391050_at | NM_001100898 | Mtf2 | metal response element binding transcription factor 2 | 1.27 |
| 1373426_at | NM_053842 | Mapk1 | mitogen activated protein kinase 1 | 1.27 |
| 1372627_at | NM_001013051 | Sugt1 | SGT1, suppressor of G2 allele of SKP1 (S. cerevisiae) | 1.27 |
| 1370221_at | NM_031716 | Wisp1 | WNT1 inducible signaling pathway protein 1 | 1.27 |
| 1382615_at | NM_199256 | Sec61a1 | Sec61 alpha 1 subunit (S. cerevisiae) | 1.27 |
| 1382154_at | NM_057115 | Ptpn12 | protein tyrosine phosphatase, non-receptor type 12 | 1.27 |
| 1392486_at | NM_001004267 | Tmem106b | transmembrane protein 106B | 1.26 |
| 1375896_at | NM_001109307 | Stradb | STE20-related kinase adaptor beta | 1.26 |
| 1389557_at | NM_001017537 | Tex261 | testis expressed 261 | 1.26 |
| 1374538_at | NM_001007747 | Pomgnt1 | protein O-linked mannose beta1,2-N-acetylglucosaminyltransferase | 1.26 |
| 1372093_at | NM_013160 /// XM_002728898 | LOC100360898 /// Mxi1 | Max interactor 1-like /// MAX interactor 1 | 1.26 |
| 1368668_at | NM_053866 | Plaa | phospholipase A2, activating protein | 1.26 |
| 1390365_at | NM_001106218 | Leng1 | leukocyte receptor cluster (LRC) member 1 | 1.26 |
| 1388324_at | NM_001082580 /// NM_182668 | Nit1 | nitrilase 1 | 1.26 |
| 1378326_at | NM_001014257 | Trub2 | TruB pseudouridine (psi) synthase homolog 2 (E. coli) | 1.25 |
| 1384011_a_at | NM_001082574 | Fastkd3 | FAST kinase domains 3 | 1.25 |
| 1375550_at | NM_001106647 | Bag1 | BCL2-associated athanogene | 1.25 |
| 1373911_at | NM_001108550 | Postn | periostin, osteoblast specific factor | 1.25 |
| 1393352_at | NM_001106838 | RGD1310552 | similar to hypothetical protein MGC38960 | 1.25 |
| 1398829_at | NM_013102 | Fkbp1a | FK506 binding protein 1a | 1.25 |
| 1379255_at | XM_001060853 /// XM_217592 | Atp6ap2 | ATPase, H+ transporting, lysosomal accessory protein 2 | 1.25 |
| 1380548_at | NM_001107093 | Ift57 | intraflagellar transport 57 homolog (Chlamydomonas) | 1.25 |
| 1368899_at | NM_030849 | Bmpr1a | bone morphogenetic protein receptor, type IA | 1.25 |
| 1371212_at | NM_031588 | Nrg1 | neuregulin 1 | 1.25 |
| 1398395_at | NM_001106719 | Itgb1bp1 | integrin beta 1 binding protein 1 | 1.25 |
| 1393760_at | NM_001168641 | Wars2 | tryptophanyl tRNA synthetase 2 (mitochondrial) | 1.25 |
| 1389608_at | NM_001109666 | Abcf2 | ATP-binding cassette, sub-family F (GCN20), member 2 | 1.25 |
| 1376690_at | NM_001107895 | Med21 | mediator complex subunit 21 | 1.25 |
| 1374874_at | XR_085615 /// XR_086012 | RGD1561042 | similar to RIKEN cDNA 5730509K17 gene | 1.25 |
| 1370193_at | NM_031579 | Ptp4a1 | protein tyrosine phosphatase 4a1 | 1.25 |
| 1388517_at | NM_001024865 | Mrpl40 | mitochondrial ribosomal protein L40 | 1.25 |
| 1383782_at | NM_001108702 | Spast | spastin | 1.25 |
| 1398473_at | NM_001037349 | Bloc1s2 | biogenesis of lysosomal organelles complex-1, subunit 2 | 1.24 |
| 1377100_at | NM_001101805 /// NM_001102383 | Pds5b | PDS5, regulator of cohesion maintenance, homolog B (S. cerevisiae) | 1.24 |
| 1385883_at | NM_001107097 | Zbtb11 | zinc finger and BTB domain containing 11 | 1.24 |
| 1385601_at | XM_001066552 | LOC686103 | similar to FRG1 protein (FSHD region gene 1 protein) | 1.24 |
| 1371685_at | NM_001107861 | Kbtbd2 | kelch repeat and BTB (POZ) domain containing 2 | 1.24 |
| 1377235_a_at | NM_181379 | Ppp2r5b | protein phosphatase 2, regulatory subunit B', beta isoform | 1.24 |
| 1389026_at | NM_001135013 | Ankrd42 | ankyrin repeat domain 42 | 1.24 |
| 1368571_at | NM_021997 | Clip2 | CAP-GLY domain containing linker protein 2 | 1.24 |
| 1382161_at | NM_001106340 | Mphosph10 | M-phase phosphoprotein 10 (U3 small nucleolar ribonucleoprotein) | 1.24 |
| 1390210_at | NM_022626 | Phka1 | Phosphorylase kinase, alpha 1 | 1.24 |
| 1372176_at | NM_001105713 | Prkca | protein kinase C, alpha | 1.24 |
| 1387340_at | NM_001009953 /// NM_080909 | Rtn3 | reticulon 3 | 1.24 |
| 1373898_at | XM_001068449 /// XM_002724789 /// XM_002727930 /// XM_346914 | Pan3 | PAN3 polyA specific ribonuclease subunit homolog (S. cerevisiae) | 1.24 |
| 1388341_at | NM_001012199 | Rangap1 | RAN GTPase activating protein 1 | 1.23 |
| 1393360_at | NM_001135018 | Zfp426l | zinc finger protein 426-like | 1.23 |
| 1390234_at | NM_053426 | Sf3b1 | splicing factor 3b, subunit 1 | 1.23 |
| 1382158_at | NM_001109290 | Zfp202 | zinc finger protein 202 | 1.23 |
| 1392830_at | NM_001107465 | Chd1 | chromodomain helicase DNA binding protein 1 | 1.23 |
| 1384416_at | NM_001127597 | Rnf168 | ring finger protein 168 | 1.23 |
| 1398906_at | XR_007263 /// XR_009606 | LOC287274 | sedlin-like | 1.23 |
| 1368537_at | NM_053404 | Dctn4 | dynactin 4 | 1.23 |
| 1373013_at | NM_001014148 | Paip2 | poly(A) binding protein interacting protein 2 | 1.23 |
| 1382779_at | NM_001108539 | Hace1 | HECT domain and ankyrin repeat containing, E3 ubiquitin protein ligase 1 | 1.23 |
| 1367874_at | NM_053522 | Rhoq | ras homolog gene family, member Q | 1.23 |
| 1368649_at | NM_133419 | Dkc1 | dyskeratosis congenita 1, dyskerin | 1.23 |
| 1389815_at | NM_172045 | Ppp1r14b | protein phosphatase 1, regulatory (inhibitor) subunit 14B | 1.23 |
| 1381259_at | NM_001108179 | Stag1 | stromal antigen 1 | 1.23 |
| 1372298_at | NM_001008317 | Erp44 | endoplasmic reticulum protein 44 | 1.23 |
| 1389686_at | NM_001033963 | Prkx | protein kinase, X-linked | 1.23 |
| 1391689_at | XM_002726483 /// XM_002729425 | Jarid1a | jumonji, AT rich interactive domain 1A (Rbp2 like) | 1.23 |
| 1375415_at | NM_001108976 | Lsm10 | LSM10, U7 small nuclear RNA associated | 1.23 |
| 1390379_at | NM_001009708 | Lmo4 | LIM domain only 4 | 1.22 |
| 1367591_at | NM_022540 | Prdx3 | peroxiredoxin 3 | 1.22 |
| 1373365_at | NM_001025655 | Cmpk1 | cytidine monophosphate (UMP-CMP) kinase 1 | 1.22 |
| 1368099_at | NM_053722 | Clasp2 | cytoplasmic linker associated protein 2 | 1.22 |
| 1389644_at | NM_001134842 | Wdr67 | WD repeat domain 67 | 1.22 |
| 1373155_at | NM_001013068 | Mrpl46 | mitochondrial ribosomal protein L46 | 1.22 |
| 1389217_at | NM_021849 | Rfng | RFNG O-fucosylpeptide 3-beta-N-acetylglucosaminyltransferase | 1.22 |
| 1372042_at | NM_001106164 | Cmtm3 | CKLF-like MARVEL transmembrane domain containing 3 | 1.22 |
| 1383463_at | NM_001169120 | Zfp91 | zinc finger protein 91 | 1.22 |
| 1370162_at | NM_080907 | Ppp4r1 | protein phosphatase 4, regulatory subunit 1 | 1.22 |
| 1373375_at | NM_001107546 | Dennd5a | DENN/MADD domain containing 5A | 1.22 |
| 1375843_at | XM_001054447 /// XM_579042 | Ids | iduronate 2-sulfatase | 1.22 |
| 1374079_at | NM_001108359 | Dcun1d4 | DCN1, defective in cullin neddylation 1, domain containing 4 (S. cerevisiae) | 1.21 |
| 1399114_at | NM_001107318 | Gtf2e2 | general transcription factor IIE, polypeptide 2, beta | 1.21 |
| 1368060_at | NM_031714 | Hrsp12 | heat-responsive protein 12 | 1.21 |
| 1369642_at | NM_022387 | Pafah1b2 | platelet-activating factor acetylhydrolase, isoform 1b, subunit 2 | 1.21 |
| 1370437_at | NM_139091 | Nupl1 | nucleoporin like 1 | 1.21 |
| 1395620_at | NM_001024799 | Fam133b | family with sequence similarity 133, member B | 1.21 |
| 1387152_at | NM_022186 | Nrbf2 | nuclear receptor binding factor 2 | 1.21 |
| 1370984_at | NM_001106691 | Zfp46 | zinc finger protein 46 | 1.21 |
| 1399135_at | NM_001004243 | Rpap3 | RNA polymerase II associated protein 3 | 1.21 |
| 1371044_at | NM_031080 | Pde7a | phosphodiesterase 7A | 1.21 |
| 1398811_at | NM_019213 | Jtb | jumping translocation breakpoint | 1.21 |
| 1372364_a_at | NM_001025124 | Ntan1 | N-terminal asparagine amidase | 1.21 |
| 1372877_at | NM_178101 | Plod3 | procollagen-lysine, 2-oxoglutarate 5-dioxygenase 3 | 1.21 |
| 1382104_at | NM_001107398 | Wdr33 | WD repeat domain 33 | 1.21 |
| 1388810_at | NM_001108446 | Abce1 | ATP-binding cassette, sub-family E (OABP), member 1 | 1.21 |
| 1374321_at | NM_001109161 | RGD1560108 | similar to RIKEN cDNA 2700081O15 | 1.20 |
| 1380305_at | NM_001024360 | nod3l | NOD3-like protein | 1.20 |
| 1398965_at | NM_001191080 /// XM_001061798 /// XM_001077249 | Golga4 | golgi autoantigen, golgin subfamily a, 4 | 1.20 |
| 1398976_at | XM_001077495 /// XM_577103 | Ncor1 | nuclear receptor co-repressor 1 | 1.20 |
| 1390933_a_at | NM_001013090 | Rg9mtd3 | RNA (guanine-9-) methyltransferase domain containing 3 | 1.20 |
| 1377812_a_at | NM_001011996 | C2cd2l | C2 calcium-dependent domain containing 2-like | 1.20 |
| 1373824_at | NM_199378 | Cfdp1 | craniofacial development protein 1 | 1.20 |
| 1392610_at | NM_001108658 | Topors | topoisomerase I binding, arginine/serine-rich | 1.20 |
| 1390387_at | XM_001072742 /// XM_002725986 /// XM_002725987 /// XM_002725988 /// XM_002729085 /// XM_002729086 /// XM_002729087 /// XM_215597 | Sh3d19 | SH3 domain containing 19 | 1.20 |
| 1392908_at | NM_001030054 | Akirin1 | akirin 1 | 1.20 |
| 1383171_at | NM_001008293 | Tfb2m | transcription factor B2, mitochondrial | 1.20 |
| 1375857_at | NM_001191636 /// XM_001080627 /// XM_220031 | Myof | myoferlin | 1.20 |
| 1374864_at | NM_001012046 | Spry2 | sprouty homolog 2 (Drosophila) | 1.20 |
| 1386287_at | NM_001107214 | Slain2 | SLAIN motif family, member 2 | 1.20 |
| 1372272_at | XM_001081273 | LOC692032 | Hypothetical protein LOC692032 | 1.20 |
| 1370132_at | NM_022675 | Fkbp1b | FK506 binding protein 1b | 1.20 |
| 1387678_at | NM_080766 | Nras | neuroblastoma ras oncogene | 1.20 |
| 1371401_at | NM_001109025 | Rnf5 | ring finger protein 5 | 1.20 |
| 1389639_at | XM_002726522 /// XM_002729477 | RGD1565350 | Similar to Shb protein | 1.20 |
| 1387002_at | NM_053556 | Bud31 | BUD31 homolog (S. cerevisiae) | 1.19 |
| 1370321_at | NM_031356 | Aifm1 | apoptosis-inducing factor, mitochondrion-associated 1 | 1.19 |
| 1369984_at | NM_053540 | Cox17 | COX17 cytochrome c oxidase assembly homolog (S. cerevisiae) | 1.19 |
| 1368877_at | NM_052798 | Zfp354a | zinc finger protein 354A | 1.19 |
| 1389757_at | NM_001134537 | RGD1310159 | similar to acetyl-coA dehydrogenase -related (111.6 kD) (5G231) | 1.19 |
| 1390147_at | NM_001107915 | RGD1559904 | similar to mKIAA1429 protein | 1.19 |
| 1387244_at | NM_053899 | Cgrrf1 | cell growth regulator with ring finger domain 1 | 1.19 |
| 1368470_at | NM_012960 | Ggh | gamma-glutamyl hydrolase (conjugase, folylpolygammaglutamyl hydrolase) | 1.19 |
| 1389363_at | NM_199097 | Adi1 | acireductone dioxygenase 1 | 1.19 |
| 1379353_at | NM_001106798 | Aasdhppt | aminoadipate-semialdehyde dehydrogenase-phosphopantetheinyl transferase | 1.19 |
| 1389109_at | NM_053926 | Pip4k2a | phosphatidylinositol-5-phosphate 4-kinase, type II, alpha | 1.19 |
| 1395875_at | XM_001059136 /// XM_226606 | Ttc37 | tetratricopeptide repeat domain 37 | 1.19 |
| 1398798_at | NM_022539 | Metap2 | methionyl aminopeptidase 2 | 1.19 |
| 1373462_at | NM_001106278 | Eed | embryonic ectoderm development | 1.19 |
| 1388732_at | NM_001105950 | Slc35f5 | solute carrier family 35, member F5 | 1.18 |
| 1370177_at | NM_017076 | PVR | poliovirus receptor | 1.18 |
| 1379419_at | NM_178330 | Tmem184c | transmembrane protein 184C | 1.18 |
| 1373675_at | NM_001013034 | Glrx2 | glutaredoxin 2 | 1.18 |
| 1369998_at | NM_024152 | Arf6 | ADP-ribosylation factor 6 | 1.18 |
| 1379450_at | NM_001107712 | Cttnbp2nl | CTTNBP2 N-terminal like | 1.18 |
| 1388873_at | NM_001031659 | Trip12 | thyroid hormone receptor interactor 12 | 1.18 |
| 1388325_at | NM_199386 | Atp6v1d | ATPase, H+ transporting, lysosomal V1 subunit D | 1.18 |
| 1372183_at | NM_198726 | Kpna1 | Karyopherin alpha 1 | 1.18 |
| 1383328_x_at | NM_022265 | Pdcd4 | programmed cell death 4 | 1.18 |
| 1388615_at | NM_001005765 | Rap1a | RAP1A, member of RAS oncogene family | 1.17 |
| 1371938_at | NM_001012185 | Caprin1 | Cell cycle associated protein 1 | 1.17 |
| 1391412_at | NM_001108596 | Xrn2 | 5'-3' exoribonuclease 2 | 1.17 |
| 1388523_at | NM_001100840 | Txndc12 | thioredoxin domain containing 12 (endoplasmic reticulum) | 1.17 |
| 1367551_a_at | NM_001014126 | RGD1306410 | similar to CG14980-PB | 1.17 |
| 1374387_at | NM_023972 | Arl6ip5 | ADP-ribosylation-like factor 6 interacting protein 5 | 1.17 |
| 1383491_at | NM_001108487 | Aen | apoptosis enhancing nuclease | 1.17 |
| 1398262_at | NM_012634 | Prps2 | phosphoribosyl pyrophosphate synthetase 2 | 1.17 |
| 1370181_at | NM_013019 | Rab4a | RAB4A, member RAS oncogene family | 1.17 |
| 1395013_at | NM_001107978 | RGD1559610 | similar to CGI-94 protein | 1.17 |
| 1371505_at | NM_001025633 | Hnrnpc | heterogeneous nuclear ribonucleoprotein C (C1/C2) | 1.17 |
| 1372360_at | NM_024397 | Abi1 | abl-interactor 1 | 1.17 |
| 1371478_at | NM_001013922 | RGD1307752 | similar to RIKEN cDNA 1110008F13 | 1.17 |
| 1372282_at | NM_001106411 | Gpx8 | glutathione peroxidase 8 | 1.16 |
| 1376260_at | NM_001106470 | Mettl14 | methyltransferase like 14 | 1.16 |
| 1390526_at | NM_001107944 | Klhl9 | kelch-like 9 (Drosophila) | 1.16 |
| 1370295_at | NM_138548 | Nme1 | non-metastatic cells 1, protein (NM23A) expressed in | 1.16 |
| 1389850_at | NM_001106635 | Arid1a | AT rich interactive domain 1A (SWI-like) | 1.15 |
| 1370200_at | NM_012570 | Glud1 | glutamate dehydrogenase 1 | 1.15 |
| 1368862_at | NM_033230 | Akt1 | v-akt murine thymoma viral oncogene homolog 1 | 1.15 |
| 1392967_at | NM_001024905 | MGC116121 | similar to RIKEN cDNA 2700062C07 | 1.15 |
| 1367681_at | NM_022523 | Cd151 | CD151 molecule (Raph blood group) | 1.15 |
| 1379377_at | NM_001013201 | Gdap2 | ganglioside-induced differentiation-associated-protein 2 | 1.15 |
| 1382019_at | NM_001025407 | Alg5 | asparagine-linked glycosylation 5, dolichyl-phosphate beta-glucosyltransferase homolog (S. cerevisiae) | 1.15 |
| 1367898_at | NM_080888 | Bnip3l | BCL2/adenovirus E1B interacting protein 3-like | 1.15 |
| 1383829_at | NM_001079938 | Bbx | bobby sox homolog (Drosophila) | 1.15 |
| 1373424_at | NM_001107080 | Dgcr6 | DiGeorge syndrome critical region gene 6 | 1.15 |
| 1373215_at | NM_001105814 | Abr | active BCR-related gene | 1.15 |
| 1387807_at | NM_031763 | Pafah1b1 | platelet-activating factor acetylhydrolase, isoform 1b, subunit 1 | 1.15 |
| 1376906_at | NM_001134361 | Med1 | mediator complex subunit 1 | 1.15 |
| 1374701_at | NM_001024271 | Rnf185 | ring finger protein 185 | 1.15 |
| 1388120_at | NM_001029910 | Pdcd6ip | programmed cell death 6 interacting protein | 1.15 |
| 1389780_at | NM_001177321 /// NM_017200 | Tfpi | tissue factor pathway inhibitor (lipoprotein-associated coagulation inhibitor) | 1.15 |
| 1391411_at | XM_001066238 /// XM_002726151 /// XM_575134 | RGD1560248 | Similar to formin-like 2 isoform B | 1.15 |
| 1382175_at | NM_001113542 /// NM_001113543 /// NM_001113544 | Wtap | Wilms tumor 1 associated protein | 1.14 |
| 1376168_at | NM_001106970 | Mmgt1 | membrane magnesium transporter 1 | 1.14 |
| 1373842_at | NM_001110365 | Wasl | Wiskott-Aldrich syndrome-like | 1.14 |
| 1371372_at | NM_001130989 | Ptges3 | prostaglandin E synthase 3 (cytosolic) | 1.14 |
| 1367941_at | NM_031326 | Tfam | transcription factor A, mitochondrial | 1.14 |
| 1383269_at | NM_001025667 | Rnf2 | ring finger protein 2 | 1.14 |
| 1372395_at | XM_001065952 /// XM_215517 | 40974 | Membrane-associated ring finger (C3HC4) 6 | 1.14 |
| 1373761_at | NM_001134711 /// NM_001134712 | Fam60a | family with sequence similarity 60, member A | 1.14 |
| 1390924_at | XM_002725301 /// XM_002728492 | LOC100360100 | TAF3 RNA polymerase II, TATA box binding protein (TBP)-associated factor | 1.14 |
| 1376085_at | NM_001135712 | Tmem185a | transmembrane protein 185A | 1.14 |
| 1390485_at | NM_001106372 | 40973 | membrane-associated ring finger (C3HC4) 5 | 1.13 |
| 1392454_at | NM_001106203 | Znhit6 | zinc finger, HIT type 6 | 1.13 |
| 1389468_at | NM_001108632 | Rpia | ribose 5-phosphate isomerase A | 1.13 |
| 1372217_at | NM_001024992 | Tmem199 | transmembrane protein 199 | 1.13 |
| 1387917_at | NM_145092 | Tor1aip1 | torsin A interacting protein 1 | 1.13 |
| 1371656_at | NM_182814 | Cct4 | chaperonin containing Tcp1, subunit 4 (delta) | 1.13 |
| 1384472_at | NM_001191064 /// XM_001053592 /// XM_002726676 | Heatr5b | HEAT repeat containing 5B | 1.13 |
| 1373238_at | NM_001037980 | Tada1l | transcriptional adaptor 1 (HFI1 homolog, yeast) like | 1.13 |
| 1390298_at | NM_001107652 | Snx18 | sorting nexin 18 | 1.13 |
| 1387921_at | NM_001033951 /// NM_138920 | Zc3h14 | zinc finger CCCH type containing 14 | 1.13 |
| 1379858_at | NM_001108839 | Mettl2 | methyltransferase like 2 | 1.13 |
| 1389451_at | NM_001109670 | Gpr172b | G protein-coupled receptor 172B | 1.12 |
| 1373242_at | NM_001127201 | Tbpl1 | TATA box binding protein-like 1 | 1.12 |
| 1372625_at | NM_001191946 /// XM_001071078 /// XM_574359 | Ppp1r12c | protein phosphatase 1, regulatory (inhibitor) subunit 12C | 1.12 |
| 1389434_at | XM_001074631 /// XM_235942 | Bbs9 | Bardet-Biedl syndrome 9 | 1.12 |
| 1367494_at | NM_001044235 | RGD1310899 | similar to CGI-35 protein | 1.12 |
| 1372815_at | NM_001100536 | Magoh | mago-nashi homolog, proliferation-associated (Drosophila) | 1.12 |
| 1388476_at | NM_001013193 | Tial1 | Tia1 cytotoxic granule-associated RNA binding protein-like 1 | 1.12 |
| 1371587_at | NM_001008375 | Map2k1ip1 | mitogen-activated protein kinase kinase 1 interacting protein 1 | 1.12 |
| 1390802_at | NM_001106347 | RGD1306839 | similar to RIKEN cDNA 5033414D02 | 1.12 |
| 1382115_at | XM_001053561 /// XM_220007 | Tctn3 | tectonic family member 3 | 1.12 |
| 1392918_at | NM_001105897 | Cct8 | chaperonin containing Tcp1, subunit 8 (theta) | 1.11 |
| 1398608_at | NM_001033061 | Cenpl | centromere protein L | 1.11 |
| 1389343_at | NM_001173556 /// XM_001071412 /// XM_002726642 /// XM_002726754 /// XM_002729554 /// XM_002729637 /// XM_342957 | LOC100361944 /// RGD1560187 | hypothetical protein LOC100361944 /// similar to Hypothetical UPF0327 protein | 1.11 |
| 1373978_at | NM_001014785 | Ncbp1 | nuclear cap binding protein subunit 1, 80kDa | 1.11 |
| 1369932_a_at | NM_012639 | Raf1 | v-raf-leukemia viral oncogene 1 | 1.11 |
| 1389563_at | NM_001173528 /// XM_001079323 /// XM_228065 | Trappc10 | trafficking protein particle complex 10 | 1.11 |
| 1377937_at | NM_001105963 | Mrps14 | mitochondrial ribosomal protein S14 | 1.10 |
| 1389544_at | NM_001135174 | Tomm7 | translocase of outer mitochondrial membrane 7 homolog (yeast) | 1.10 |
| 1377593_at | NM_001106544 | Dpm1 | dolichyl-phosphate mannosyltransferase polypeptide 1, catalytic subunit | 1.10 |
| 1373319_at | NM_053414 | Ddx1 | DEAD (Asp-Glu-Ala-Asp) box polypeptide 1 | 1.10 |
| 1371495_at | NM_001007700 | M6pr | mannose-6-phosphate receptor, cation dependent | 1.10 |
| 1398788_at | NM_017319 | Pdia3 | protein disulfide isomerase family A, member 3 | 1.10 |
| 1377731_at | NM_001106021 | C1d | nuclear DNA binding protein | 1.09 |
| 1373033_at | NM_001008302 | Sft2d1 | SFT2 domain containing 1 | 1.09 |
| 1393657_at | NM_001106281 | Prcp | prolylcarboxypeptidase (angiotensinase C) | 1.08 |
| 1372445_at | NM_001126095 | Srp9 | signal recognition particle 9 | 1.08 |
| 1370947_at | NM_001113747 | Setd4 | SET domain containing 4 | 1.07 |
| 1398626_s_at | NM_001009268 | Actr2 | ARP2 actin-related protein 2 homolog (yeast) | 1.07 |
| 1376575_at | NM_001108811 | Twsg1 | twisted gastrulation homolog 1 (Drosophila) | 1.06 |
| 1389454_at | NM_001106247 | Pdcd5 | programmed cell death 5 | 1.05 |
| 1383720_at | XM_002727525 /// XM_002727526 /// XM_002727527 /// XM_002727528 /// XM_002727529 /// XM_002730185 | Utx | ubiquitously transcribed tetratricopeptide repeat, X chromosome | 1.05 |
| 1392966_at | NM_001108042 | Zfp410 | zinc finger protein 410 | 1.04 |
| 1372092_at | NM_133560 | Trak2 | trafficking protein, kinesin binding 2 | 1.03 |
| 1393240_at | NM_001005907 | Efemp2 | EGF-containing fibulin-like extracellular matrix protein 2 | 0.95 |
| 1367579_a_at | NM_001011995 /// NM_001044270 /// NM_022298 | Tuba1a /// Tuba1b /// Tuba1c | tubulin, alpha 1A /// tubulin, alpha 1B /// tubulin, alpha 1C | 0.95 |
| 1375108_at |  | ND3 | NADH dehydrogenase subunit 3 | 0.94 |
| 1375912_a_at | NM_001106196 | Galnt2 | UDP-N-acetyl-alpha-D-galactosamine:polypeptide N-acetylgalactosaminyltransferase 2 (GalNAc-T2) | 0.93 |
| 1370156_at | NM_012631 | Prnp | prion protein | 0.93 |
| 1382909_at | NM_001134509 | RGD1311783 | similar to RIKEN cDNA 2010012O05 | 0.93 |
| 1367457_at | NM_001034117 /// NM_053739 | Becn1 | beclin 1, autophagy related | 0.93 |
| 1371319_at | NM_001006963 | Itm2b | integral membrane protein 2B | 0.93 |
| 1370287_a_at | NM_001034068 /// NM_001034069 /// NM_001034070 /// NM_001034071 /// NM_001034072 /// NM_001034073 /// NM_001034074 /// NM_001034075 /// NM_019131 | Tpm1 | tropomyosin 1, alpha | 0.92 |
| 1389323_at | NM_001025743 | Wdr61 | WD repeat domain 61 | 0.92 |
| 1388725_at | NM_020099 | Leprot | leptin receptor overlapping transcript | 0.92 |
| 1393987_s_at | NM_001112712 /// NM_001112713 /// NM_031657 | Grk6 | G protein-coupled receptor kinase 6 | 0.92 |
| 1371559_at | NM_001109678 | Nadk | NAD kinase | 0.91 |
| 1367900_at | NM_031043 | Gyg1 | glycogenin 1 | 0.91 |
| 1399091_at | NM_001005903 | Capzb | capping protein (actin filament) muscle Z-line, beta | 0.90 |
| 1373228_at | XR_085644 /// XR_086050 | RGD1560755 | similar to D8Ertd354e protein | 0.89 |
| 1371662_at | NM_001006967 | Kars | lysyl-tRNA synthetase | 0.89 |
| 1380008_at | NM_001107329 | Zfp828 | zinc finger protein 828 | 0.89 |
| 1371725_at | NM_013194 | Myh9 | Myosin, heavy chain 9, non-muscle | 0.89 |
| 1371362_at | NM_001015018 | Ddx17 | DEAD (Asp-Glu-Ala-Asp) box polypeptide 17 | 0.89 |
| 1374547_at | NM_001098782 | Serf2 | small EDRK-rich factor 2 | 0.89 |
| 1388113_at | NM_134345 | Cox8a | cytochrome c oxidase subunit VIIIa | 0.89 |
| 1379036_at | NM_001031643 | Trdmt1 | tRNA aspartic acid methyltransferase 1 | 0.88 |
| 1375245_at | NM_057140 | Ppp2r1a | protein phosphatase 2 (formerly 2A), regulatory subunit A, alpha isoform | 0.88 |
| 1376638_at | NM_001009676 | Anks3 | ankyrin repeat and sterile alpha motif domain containing 3 | 0.88 |
| 1374436_at | NM_001008289 | Sbds | Shwachman-Bodian-Diamond syndrome homolog (human) | 0.88 |
| 1387891_at | NM_053512 | Prdx4 | peroxiredoxin 4 | 0.88 |
| 1371334_at | NM_001009674 | Itm2c | integral membrane protein 2C | 0.88 |
| 1393905_at | NM_001024770 | Sec3l1 | SEC3-like 1 (S. cerevisiae) | 0.88 |
| 1371414_at | NM_001004080 | Gsn | gelsolin | 0.88 |
| 1391014_at | NM_198770 | Zmynd19 | zinc finger, MYND-type containing 19 | 0.88 |
| 1392590_at | NM_001012032 | Arhgap24 | Rho GTPase activating protein 24 | 0.87 |
| 1368379_at | NM_054001 | Scarb2 | scavenger receptor class B, member 2 | 0.87 |
| 1371740_at | NM_001012182 | Sike | suppressor of IKK epsilon | 0.87 |
| 1388838_at | XM_001073409 /// XM_002726259 | Chmp4b | chromatin modifying protein 4B | 0.87 |
| 1372835_at | NM_001008320 | Rhoj | ras homolog gene family, member J | 0.87 |
| 1373492_at | NM_001008371 | Sdhaf2 | succinate dehydrogenase complex assembly factor 2 | 0.87 |
| 1390755_at | XM_001080802 /// XM_344891 | Sergef | secretion regulating guanine nucleotide exchange factor | 0.87 |
| 1395285_at | XM_002725224 /// XR_086059 | LOC100361684 /// LOC100365086 | hypothetical LOC100361684 /// RIKEN cDNA 4732471D19-like | 0.87 |
| 1391978_at | NM_001170327 /// NM_001170328 | Son | Son DNA binding protein | 0.87 |
| 1387892_at | NM_173102 | Tubb5 | tubulin, beta 5 | 0.87 |
| 1377049_at | NM_144738 | Pnpla7 | patatin-like phospholipase domain containing 7 | 0.87 |
| 1390046_at | NM_001107560 | Tubgcp2 | tubulin, gamma complex associated protein 2 | 0.86 |
| 1367863_at | NM_080897 | Bnip1 | BCL2/adenovirus E1B interacting protein 1 | 0.86 |
| 1384383_at | NM_001047849 | Agpat6 | 1-acylglycerol-3-phosphate O-acyltransferase 6 (lysophosphatidic acid acyltransferase, zeta) | 0.86 |
| 1369679_a_at | NM_012988 | Nfia | nuclear factor I/A | 0.86 |
| 1382223_at | NM_001107982 | Zmym4 | zinc finger, MYM-type 4 | 0.86 |
| 1389546_at | NM_031717 | Amotl2 | angiomotin like 2 | 0.86 |
| 1367931_a_at | NM_022516 | Ptbp1 | polypyrimidine tract binding protein 1 | 0.86 |
| 1379590_at | NM_001017504 | Zrsr1 | zinc finger (CCCH type), RNA binding motif and serine/arginine rich 1 | 0.86 |
| 1375173_at | NM_139255 | Plbd2 | phospholipase B domain containing 2 | 0.86 |
| 1373209_at | NM_001024970 | RGD1305572 | similar to hypothetical protein MGC30618 | 0.86 |
| 1372767_at | XM_001073605 /// XM_002727216 /// XM_578699 | Fam168b /// LOC503175 | family with sequence similarity 168, member B /// similar to Protein KIAA0280 | 0.86 |
| 1370277_at | NM_139100 | Slc25a3 | solute carrier family 25 (mitochondrial carrier, phosphate carrier), member 3 | 0.86 |
| 1398797_at | NM_057141 | Hnrnpk | heterogeneous nuclear ribonucleoprotein K | 0.86 |
| 1393336_at | NM_001106288 | Swap70 | SWAP switching B-cell complex 70 | 0.85 |
| 1372232_at | NM_001106600 | Wbp1 | WW domain binding protein 1 | 0.85 |
| 1371626_at | NM_001108840 | Srp68 | signal recognition particle 68 | 0.85 |
| 1376674_at | NM_001107527 | Man2a2 | Mannosidase 2, alpha 2 | 0.85 |
| 1373623_at | NM_178094 | Itpkc | inositol 1,4,5-trisphosphate 3-kinase C | 0.85 |
| 1373455_at | NM_001024898 | Paf1 | Paf1, RNA polymerase II associated factor, homolog (S. cerevisiae) | 0.85 |
| 1389365_at | XM_001073988 /// XM_002725609 /// XM_002728734 | LOC690000 | similar to CG3740-PA | 0.85 |
| 1385787_at | NM_001001800 | rnf141 | ring finger protein 141 | 0.85 |
| 1371916_at | NM_001044285 | Sepx1 | selenoprotein X, 1 | 0.85 |
| 1379849_at | NM_001106059 | Thoc3 | THO complex 3 | 0.85 |
| 1379085_at | XM_002726270 /// XM_002729218 | RGD1561878 | similar to mKIAA0978 protein | 0.85 |
| 1371425_at | NM_001107986 | Srrm1 | serine/arginine repetitive matrix 1 | 0.85 |
| 1399110_at | NM_001009172 | Zbtb22 | zinc finger and BTB domain containing 22 | 0.85 |
| 1399138_at | NM_001014231 | Dcaf8 | DDB1 and CUL4 associated factor 8 | 0.85 |
| 1379364_at | NM_001168549 | Camsap1 | calmodulin regulated spectrin-associated protein 1 | 0.84 |
| 1376502_at | NM_001017451 | RGD1309228 | similar to putative protein, with at least 9 transmembrane domains, of eukaryotic origin (43.9 kD) (2G415) | 0.84 |
| 1384648_at | NM_001107661 | Fam164a | Family with sequence similarity 164, member A | 0.84 |
| 1370190_at | NM_053985 | H3f3b | H3 histone, family 3B | 0.84 |
| 1371752_at | NM_001105790 | Rangrf | RAN guanine nucleotide release factor | 0.84 |
| 1379028_at | NM_001107016 | Spag7 | sperm associated antigen 7 | 0.83 |
| 1398286_at | NM_001134454 /// NM_021750 | Csad | cysteine sulfinic acid decarboxylase | 0.83 |
| 1370298_at | NM_001109047 | Ccdc56 | coiled-coil domain containing 56 | 0.83 |
| 1373750_at | NM_001106620 | Leprel2 | leprecan-like 2 | 0.83 |
| 1372746_at | NM_001107305 | RGD1566239 | similar to RIKEN cDNA 2810428I15 | 0.83 |
| 1389520_at | NM_001014135 | Wdr1 | WD repeat domain 1 | 0.83 |
| 1388401_at | NM_001107288 | Flnb | filamin, beta | 0.83 |
| 1377777_at | NM_001107453 | Brd9 | bromodomain containing 9 | 0.83 |
| 1387111_at | NM_022297 | Ddah1 | dimethylarginine dimethylaminohydrolase 1 | 0.83 |
| 1388531_at | NM_001008374 | Pgrmc2 | progesterone receptor membrane component 2 | 0.83 |
| 1373254_at | NM_001008804 | Krt10 | keratin 10 | 0.83 |
| 1388647_at | XM_001069610 | LOC684258 | similar to coiled-coil-helix-coiled-coil-helix domain containing 7 | 0.83 |
| 1389334_at | NM_182671 /// NM_199495 | Ndufa10 /// Ndufa10l1 | NADH dehydrogenase (ubiquinone) 1 alpha subcomplex 10 /// NADH dehydrogenase (ubiquinone) 1 alpha subcomplex 10-like 1 | 0.83 |
| 1372219_at | NM_001024345 | Tpm2 | tropomyosin 2, beta | 0.83 |
| 1391530_a_at | NM_001100508 | Oxsm | 3-oxoacyl-ACP synthase, mitochondrial | 0.82 |
| 1375700_at | NM_001107079 | Klhl22 | kelch-like 22 (Drosophila) | 0.82 |
| 1371810_at | NM_001031822 | Copg | Coatomer protein complex, subunit gamma | 0.82 |
| 1398769_at | NM_019222 | Coro1b | coronin, actin-binding protein, 1B | 0.82 |
| 1389621_at | NM_001008295 | Fip1l1 | FIP1 like 1 (S. cerevisiae) | 0.82 |
| 1378638_a_at | NM_001169105 | RGD1311747 | similar to 2700029M09Rik protein | 0.82 |
| 1367697_at | NM_031020 | Mapk14 | mitogen activated protein kinase 14 | 0.82 |
| 1371012_at | NM_053493 | Hacl1 | 2-hydroxyacyl-CoA lyase 1 | 0.82 |
| 1384443_at | NM_001106499 | Vps18 | vacuolar protein sorting 18 homolog (S. cerevisiae) | 0.82 |
| 1385564_at | NM_001013864 | Lpp | LIM domain containing preferred translocation partner in lipoma | 0.82 |
| 1389598_at | NM_001191794 /// XM_001074158 /// XM_236325 | Cln6 | ceroid-lipofuscinosis, neuronal 6 | 0.82 |
| 1371251_at | NM_001013089 | Galt | galactose-1-phosphate uridylyltransferase | 0.82 |
| 1377899_at | NM_001113775 | RGD1304982 | similar to RIKEN cDNA 2810025M15 | 0.82 |
| 1372475_at | NM_001106694 | Pink1 | PTEN induced putative kinase 1 | 0.82 |
| 1373253_at | NM_001012013 | Acbd4 | acyl-Coenzyme A binding domain containing 4 | 0.81 |
| 1388118_at | NM_022243 | Hibadh | 3-hydroxyisobutyrate dehydrogenase | 0.81 |
| 1368399_a_at | NM_031640 | Pgcp | plasma glutamate carboxypeptidase | 0.81 |
| 1390524_at | NM_001024892 | Rlim | ring finger protein, LIM domain interacting | 0.81 |
| 1383358_at | NM_053665 | Akap1 | A kinase (PRKA) anchor protein 1 | 0.81 |
| 1369975_at | NM_023027 | Secp43 | tRNA selenocysteine associated protein | 0.81 |
| 1398782_at | NM_080585 | Napa | N-ethylmaleimide-sensitive factor attachment protein, alpha | 0.81 |
| 1390573_a_at | NM_001107264 | Nfatc4 | nuclear factor of activated T-cells, cytoplasmic, calcineurin-dependent 4 | 0.81 |
| 1367663_at | NM_017264 | Psme1 | proteasome (prosome, macropain) activator subunit 1 | 0.81 |
| 1387114_at | NM_133307 | Prkcd | protein kinase C, delta | 0.81 |
| 1393148_at | NM_001105978 | Gpr137b | G protein-coupled receptor 137B | 0.81 |
| 1371336_at | NM_001005876 | Hn1 | hematological and neurological expressed 1 | 0.81 |
| 1392223_at | NM_001033914 /// NM_021852 | Epn2 | epsin 2 | 0.81 |
| 1373151_at | NM_001109183 | Lhfp | lipoma HMGIC fusion partner | 0.81 |
| 1395535_at | NM_001014073 | Fam98a | family with sequence similarity 98, member A | 0.81 |
| 1376581_at | NM_001108981 | RGD1309138 | similar to hypothetical protein MGC9912 | 0.81 |
| 1372205_at | NM_001107231 | Zfp278 | zinc finger protein 278 | 0.81 |
| 1379370_at | NM_001109255 | Sspn | sarcospan | 0.80 |
| 1374602_at | NM_001013033 | Tspyl1 | TSPY-like 1 | 0.80 |
| 1374441_at | NM_001037765 | Spryd4 | SPRY domain containing 4 | 0.80 |
| 1384084_at | NM_001134575 | RGD1308106 | LOC361719 | 0.80 |
| 1389570_at | NM_001013859 | Inpp5k | inositol polyphosphate-5-phosphatase K | 0.80 |
| 1374947_at | NM_001107722 | Bcar3 | breast cancer anti-estrogen resistance 3 | 0.80 |
| 1374294_at | XM_001078497 /// XM_002728812 | LOC687796 | hypothetical protein LOC687796 | 0.80 |
| 1371813_at | NM_001025725 | Hirip3 | HIRA interacting protein 3 | 0.80 |
| 1383181_at | NM_001108865 | Dnajc9 | DnaJ (Hsp40) homolog, subfamily C, member 9 | 0.80 |
| 1375559_at | NM_001127570 | LOC686323 | similar to thyroid receptor-interacting protein 6 | 0.80 |
| 1371754_at | NM_145677 | Slc25a25 | solute carrier family 25 (mitochondrial carrier, phosphate carrier), member 25 | 0.80 |
| 1385046_at | NM_001109435 | Fam55c | family with sequence similarity 55, member C | 0.80 |
| 1398387_at | NM_199105 | Fam198b | family with sequence similarity 198, member B | 0.80 |
| 1371816_at | XM_001072887 /// XM_002726251 | Zcchc3 | zinc finger, CCHC domain containing 3 | 0.80 |
| 1370318_at | NM_022301 | Pi4ka | phosphatidylinositol 4-kinase, catalytic, alpha | 0.80 |
| 1380203_at | NM_001107809 | Znf512b | zinc finger protein 512B | 0.80 |
| 1371681_at | NM_173324 | Tox4 | TOX high mobility group box family member 4 | 0.80 |
| 1387907_at | NM_001007235 | Itpr1 | inositol 1,4,5-triphosphate receptor, type 1 | 0.80 |
| 1377602_at | NM_207596 | Nudt2 | nudix (nucleoside diphosphate linked moiety X)-type motif 2 | 0.79 |
| 1378399_at | NM_001017485 | LOC498145 | similar to RIKEN cDNA 2810453I06 | 0.79 |
| 1371963_at | NM_019330 | Pcca | propionyl-coenzyme A carboxylase, alpha polypeptide | 0.79 |
| 1387919_at | NM_130894 | Mfn2 | mitofusin 2 | 0.79 |
| 1377116_at | NM_182673 | Rnasel | Ribonuclease L (2',5'-oligoisoadenylate synthetase-dependent) | 0.79 |
| 1374112_at | NM_001122782 | Mapk1ip1 | mitogen-activated protein kinase 1 interacting protein 1 | 0.79 |
| 1388936_at | NM_053392 | Cdh11 | cadherin 11 | 0.79 |
| 1392957_at | XM_001065491 /// XM_342171 | Ssbp2 | single-stranded DNA binding protein 2 | 0.79 |
| 1388300_at | NM_001191594 /// XM_001076104 /// XM_213943 | Mgst3 | microsomal glutathione S-transferase 3 | 0.79 |
| 1373335_at | NM_001039016 | Zdhhc9 | zinc finger, DHHC-type containing 9 | 0.79 |
| 1371402_at | NM_057213 | Atp6v1b2 | ATPase, H transporting, lysosomal V1 subunit B2 | 0.78 |
| 1376337_at | NM_001004446 | Smarca2 | SWI/SNF related, matrix associated, actin dependent regulator of chromatin, subfamily a, member 2 | 0.78 |
| 1384423_at | XM_002728891 | LOC100363253 | 5'-nucleotidase, cytosolic II | 0.78 |
| 1371633_at | NM_001024870 | Ctnnbl1 | catenin, beta like 1 | 0.78 |
| 1381983_at | NM_001191747 /// XM_001054730 /// XM_231184 | Dennd1a | DENN/MADD domain containing 1A | 0.78 |
| 1375279_at | NM_001024903 | Sertad2 | SERTA domain containing 2 | 0.78 |
| 1387870_at | NM_133290 | Zfp36 | zinc finger protein 36 | 0.78 |
| 1376700_at | NM_001191615 /// XM_001064221 /// XM_217039 | Lima1 | LIM domain and actin binding 1 | 0.78 |
| 1397588_at | XM_001077638 /// XM_002724481 /// XM_002727702 /// XM_340757 | RGD1561796 | RGD1561796 | 0.78 |
| 1395316_at | NM_001013250 | Mageh1 | melanoma antigen, family H, 1 | 0.78 |
| 1383217_at | XM_002724888 /// XM_002728000 | RGD1564952 | similar to zinc finger, RAN-binding domain containing 3 | 0.78 |
| 1391425_at | NM_001106857 | Nckipsd | NCK interacting protein with SH3 domain | 0.78 |
| 1370415_at | NM_019365 | Rassf5 | Ras association (RalGDS/AF-6) domain family member 5 | 0.77 |
| 1367872_at | NM_017277 | Ap1b1 | adaptor-related protein complex 1, beta 1 subunit | 0.77 |
| 1367522_at | NM_001109323 | F8a1 | coagulation factor VIII-associated (intronic transcript) 1 | 0.77 |
| 1375739_at | NM_139324 | Ehd4 | EH-domain containing 4 | 0.77 |
| 1388566_at | NM_032613 | Lasp1 | LIM and SH3 protein 1 | 0.77 |
| 1389282_at | NM_001108292 | Itga3 | Integrin, alpha 3 | 0.77 |
| 1374624_at | NM_199393 | Galnt11 | UDP-N-acetyl-alpha-D-galactosamine:polypeptide N-acetylgalactosaminyltransferase 11 (GalNAc-T11) | 0.77 |
| 1398622_at | NM_001169103 | Crim1 | Cysteine rich transmembrane BMP regulator 1 (chordin like) | 0.77 |
| 1375767_at | XM_001080822 /// XM_002726757 /// XM_002729654 /// XM_234320 | Plekhg3 | pleckstrin homology domain containing, family G (with RhoGef domain) member 3 | 0.77 |
| 1383349_at | NM_001107694 | Rfx5 | regulatory factor X, 5 (influences HLA class II expression) | 0.77 |
| 1392568_at | XM_001081219 /// XM_220843 | RGD1562272 | similar to TAF11 RNA polymerase II, TATA box binding protein (TBP)-associated factor | 0.77 |
| 1371602_at | NM_001107890 | Tspan9 | tetraspanin 9 | 0.77 |
| 1372297_at | NM_001106840 | Gsta4 | glutathione S-transferase alpha 4 | 0.76 |
| 1390915_at | XM_001053814 /// XM_002726191 /// XM_002730290 /// XM_236536 | Morc4 | MORC family CW-type zinc finger 4 | 0.76 |
| 1379239_at | NM_001013884 | Uimc1 | Ubiquitin interaction motif containing 1 | 0.76 |
| 1393020_at | XM_002726625 /// XM_002729531 | LOC100361631 | hypothetical protein LOC100361631 | 0.76 |
| 1384335_at | NM_001106817 | Bcl9l | B-cell CLL/lymphoma 9-like | 0.76 |
| 1375845_at | NM_001134425 | Aig1 | androgen-induced 1 | 0.76 |
| 1370886_a_at | NM_001081972 /// NM_001081973 /// NM_001081974 | Klc1 | kinesin light chain 1 | 0.76 |
| 1392542_at | NM_001126089 | Cdc42se2 | CDC42 small effector 2 | 0.76 |
| 1371027_at | NM_133601 | Cblb | Cas-Br-M (murine) ecotropic retroviral transforming sequence b | 0.76 |
| 1375847_at | NM_001127554 | Nudt16 | nudix (nucleoside diphosphate linked moiety X)-type motif 16 | 0.76 |
| 1398708_at | NM_001107675 | Maml3 | Mastermind like 3 (Drosophila) | 0.76 |
| 1369735_at | NM_057100 | Gas6 | growth arrest specific 6 | 0.76 |
| 1387354_at | NM_001012226 /// NM_001034164 /// NM_032612 | Stat1 /// Stat4 | signal transducer and activator of transcription 1 /// signal transducer and activator of transcription 4 | 0.76 |
| 1385274_at | XM_001056051 /// XM_001065373 | Pcdhb19 | protocadherin beta 19 | 0.76 |
| 1387855_at | NM_017088 | Gdi1 | GDP dissociation inhibitor 1 | 0.76 |
| 1373394_at | NM_001167806 | Smpd4 | sphingomyelin phosphodiesterase 4 | 0.76 |
| 1375417_at | NM_001100838 | Zmynd8 | zinc finger, MYND-type containing 8 | 0.75 |
| 1394310_at | NM_001106215 | Smoc2 | SPARC related modular calcium binding 2 | 0.75 |
| 1396407_at | NM_001039030 | Gas8 | growth arrest specific 8 | 0.75 |
| 1381224_at | XM_001078065 | LOC691396 | Similar to Zinc finger protein 551 (Zinc finger protein KOX23) | 0.75 |
| 1374403_at | NM_017089 | Efnb1 | ephrin B1 | 0.75 |
| 1399078_at | NM_001106768 | Med16 | mediator complex subunit 16 | 0.75 |
| 1398330_at | NM_013038 | Stxbp1 | syntaxin binding protein 1 | 0.75 |
| 1369197_at | NM_023979 | Apaf1 | apoptotic peptidase activating factor 1 | 0.75 |
| 1391858_at | NM_001034924 | Sfrs8 | splicing factor, arginine/serine-rich 8 | 0.75 |
| 1371738_at | XM_001078269 /// XM_236774 | Ano10 | anoctamin 10 | 0.75 |
| 1374239_at | NM_001108233 | Farp2 | FERM, RhoGEF and pleckstrin domain protein 2 | 0.75 |
| 1375523_at | XM_002728965 | Marcks | Myristoylated alanine rich protein kinase C substrate | 0.75 |
| 1368302_at | NM_031059 | Msx1 | msh homeobox 1 | 0.75 |
| 1371531_at | XM_001053139 | LOC678880 | similar to mammalian retrotransposon derived 8b | 0.74 |
| 1371947_at | NM_001008558 | Ndn | necdin homolog (mouse) | 0.74 |
| 1385369_at | NM_001014095 | Dzip1l | DAZ interacting protein 1-like | 0.74 |
| 1375023_at | XM_001080668 /// XM_341852 | Prr12 | proline rich 12 | 0.74 |
| 1384132_at | NM_001012201 | Cadm1 | cell adhesion molecule 1 | 0.74 |
| 1371042_at | NM_133407 | Map4k3 | mitogen-activated protein kinase kinase kinase kinase 3 | 0.74 |
| 1387377_a_at | NM_017198 | Pak1 | p21 protein (Cdc42/Rac)-activated kinase 1 | 0.74 |
| 1386992_at | NM_017175 | Pkn1 | protein kinase N1 | 0.74 |
| 1375212_at | NM_001191875 /// XM_001070685 /// XM_343139 | Ankrd52 | Ankyrin repeat domain 52 | 0.74 |
| 1378238_at | NM_001107771 | Anapc1 | anaphase promoting complex subunit 1 | 0.74 |
| 1373934_at | NM_001108747 | Rassf3 | Ras association (RalGDS/AF-6) domain family member 3 | 0.74 |
| 1383689_at | NM_001012018 | B4galt4 | UDP-Gal:betaGlcNAc beta 1,4-galactosyltransferase, polypeptide 4 | 0.74 |
| 1373921_at | NM_001101010 | Echdc3 | enoyl Coenzyme A hydratase domain containing 3 | 0.74 |
| 1370952_at | NM_177426 | Gstm2 | glutathione S-transferase mu 2 | 0.74 |
| 1373527_at | XM_002725783 /// XM_002728863 | LOC100362920 | rCG48622-like | 0.74 |
| 1370819_at | NM_139060 | Csnk1d | casein kinase 1, delta | 0.74 |
| 1376658_at | NM_001108798 | Raph1 | Ras association (RalGDS/AF-6) and pleckstrin homology domains 1 | 0.73 |
| 1388350_at | NM_001107375 /// NM_001134777 | Pex19 | peroxisomal biogenesis factor 19 | 0.73 |
| 1388542_at | NM_001169100 /// XM_574708 | LOC499392 /// Use1 | hypothetical LOC499392 /// unconventional SNARE in the ER 1 homolog (S. cerevisiae) | 0.73 |
| 1390483_at | NM_001010958 | Slc25a29 | solute carrier family 25, member 29 | 0.73 |
| 1388991_at | NM_001134514 | Sestd1 | SEC14 and spectrin domains 1 | 0.73 |
| 1389229_at | NM_001007710 | Acpl2 | acid phosphatase-like 2 | 0.73 |
| 1371707_at | NM_001107166 | Tnpo2 | transportin 2 (importin 3, karyopherin beta 2b) | 0.73 |
| 1372549_at | NM_001128155 | Tmem88 | transmembrane protein 88 | 0.73 |
| 1389145_at | NM_001009689 | Cdc42ep2 | CDC42 effector protein (Rho GTPase binding) 2 | 0.73 |
| 1386914_at | NM_057188 | Gmpr | guanosine monophosphate reductase | 0.73 |
| 1374668_at | NM_001107235 | Sf3a1 | splicing factor 3a, subunit 1 | 0.73 |
| 1368345_at | NM_017204 | Map6 | microtubule-associated protein 6 | 0.73 |
| 1372643_at | XM_001053351 /// XM_220117 | Epb4.1l2 | erythrocyte membrane protein band 4.1-like 2 | 0.73 |
| 1388918_at | NM_172039 | Hdlbp | High density lipoprotein binding protein | 0.73 |
| 1382312_at | NM_001107624 | Arid5b | AT rich interactive domain 5B (Mrf1 like) | 0.72 |
| 1375910_at | NM_001048044 | Cdc42ep3 | CDC42 effector protein (Rho GTPase binding) 3 | 0.72 |
| 1389447_at | XM_001075237 /// XM_217285 | P4htm | prolyl 4-hydroxylase, transmembrane | 0.72 |
| 1370129_at | NM_131904 | Mgea5 | meningioma expressed antigen 5 (hyaluronidase) | 0.72 |
| 1372905_at | NM_001107248 | Vcl | vinculin | 0.72 |
| 1387129_at | NM_053435 | Xrcc1 | X-ray repair complementing defective repair in Chinese hamster cells 1 | 0.72 |
| 1389170_at | NM_022260 | Casp7 | caspase 7 | 0.72 |
| 1373112_at | NM_001037191 | Tecpr1 | Tectonin beta-propeller repeat containing 1 | 0.72 |
| 1373371_a_at | NM_001037795 | Fmc1 | formation of mitochondrial complexes 1 homolog (S. cerevisiae) | 0.72 |
| 1371744_at | XM_001055035 | LOC679203 | similar to Negative elongation factor D (NELF-D) (TH1-like protein) | 0.72 |
| 1376062_at | NM_013026 | Sdc1 | syndecan 1 | 0.72 |
| 1388034_at | NM_057200 | Kif1b /// Pgd | kinesin family member 1B /// phosphogluconate dehydrogenase | 0.72 |
| 1393349_x_at | NM_001106563 | Vav2 | vav 2 guanine nucleotide exchange factor | 0.71 |
| 1388831_at | NM_053811 | Slc9a3r2 | solute carrier family 9 (sodium/hydrogen exchanger), member 3 regulator 2 | 0.71 |
| 1370857_at | NM_031004 | Acta2 | smooth muscle alpha-actin | 0.71 |
| 1372995_at | NM_001013895 | Prkd2 | protein kinase D2 | 0.71 |
| 1370638_at | NM_001033984 /// NM_031805 | Ank3 | ankyrin 3, epithelial | 0.71 |
| 1388480_at | NM_001134413 | Gltp | glycolipid transfer protein | 0.71 |
| 1389814_at | XR_005443 /// XR_009642 | LOC501546 | hypothetical protein LOC501546 | 0.71 |
| 1388356_at | NM_001108557 | S100a16 | S100 calcium binding protein A16 | 0.71 |
| 1368390_at | NM_001033663 /// NM_022532 | Araf | v-raf murine sarcoma 3611 viral oncogene homolog | 0.71 |
| 1388837_at | NM_001134715 | Slc44a2 | solute carrier family 44, member 2 | 0.71 |
| 1369768_at | NM_133285 | Hist1h1d | histone cluster 1, H1d | 0.71 |
| 1388813_at | NM_024150 | Arf2 | ADP-ribosylation factor 2 | 0.71 |
| 1387225_at | NM_133585 | Opa1 | optic atrophy 1 homolog (human) | 0.71 |
| 1375706_at | XM_002728724 | LOC100361913 | rCG54286-like | 0.71 |
| 1391604_at | NM_001034000 | Ncapd3 | non-SMC condensin II complex, subunit D3 | 0.70 |
| 1370910_at | NM_053786 | Rfc2 | replication factor C (activator 1) 2 | 0.70 |
| 1391419_at | NM_001134956 | Ahdc1 | AT hook, DNA binding motif, containing 1 | 0.70 |
| 1372487_at | NM_001034958 | Fam104a | family with sequence similarity 104, member A | 0.70 |
| 1383433_at | NM_001134504 | Klhl23 | kelch-like 23 (Drosophila) | 0.70 |
| 1392405_at | NM_001106847 | Tfdp2 | transcription factor Dp-2 (E2F dimerization partner 2) | 0.70 |
| 1372277_at | NM_001134698 | LOC641316 | similar to aldehyde dehydrogenase 4 family, member A1 | 0.70 |
| 1383309_at | NM_207602 | St3gal6 | ST3 beta-galactoside alpha-2,3-sialyltransferase 6 | 0.70 |
| 1370127_at | NM_021662 | Pold1 | polymerase (DNA directed), delta 1, catalytic subunit | 0.70 |
| 1390891_at | NM_001169112 | Kif11 | kinesin family member 11 | 0.70 |
| 1370461_at | NM_012964 | Hmmr | hyaluronan mediated motility receptor (RHAMM) | 0.70 |
| 1388165_at | NM_173122 | Porf-2 | preoptic regulatory factor-2 | 0.70 |
| 1376249_at | NM_001004218 | Fuca2 | fucosidase, alpha-L- 2, plasma | 0.70 |
| 1387964_a_at | NM_138528 | Ero1l | ERO1-like (S. cerevisiae) | 0.70 |
| 1386880_at | NM_130433 | Acaa2 | acetyl-Coenzyme A acyltransferase 2 | 0.70 |
| 1371527_at | NM_012843 | Emp1 | epithelial membrane protein 1 | 0.70 |
| 1374312_at | NM_001107831 | Uck1 | uridine-cytidine kinase 1 | 0.70 |
| 1374033_at | NM_001025637 | Psmb10 | proteasome (prosome, macropain) subunit, beta type 10 | 0.70 |
| 1370237_at | NM_057186 | Hadh | hydroxyacyl-Coenzyme A dehydrogenase | 0.69 |
| 1369974_at | NM_012663 | Vamp2 | vesicle-associated membrane protein 2 | 0.69 |
| 1390938_at | NM_001191815 /// XM_001054365 /// XM_237535 | Arhgap28 | Rho GTPase activating protein 28 | 0.69 |
| 1383953_at | NM_001106910 | Ercc5 | excision repair cross-complementing rodent repair deficiency, complementation group 5 | 0.69 |
| 1388955_at | NM_001024897 | Ehd2 | EH-domain containing 2 | 0.69 |
| 1378184_at | XM_001067018 /// XM_577181 | Cd200r2 | Cd200 receptor 2 | 0.69 |
| 1388213_a_at | NM_001008886 | RT1-S3 | RT1 class Ib, locus S3 | 0.69 |
| 1370224_at | NM_012747 | Stat3 | signal transducer and activator of transcription 3 | 0.69 |
| 1367881_at | NM_013016 | Sirpa | signal-regulatory protein alpha | 0.69 |
| 1390729_x_at | NM_001014245 | Cpsf7 | cleavage and polyadenylation specific factor 7, 59kDa | 0.69 |
| 1373301_at | NM_001108062 | Ppp1r13b | protein phosphatase 1, regulatory (inhibitor) subunit 13B | 0.69 |
| 1392970_at | XM_001069716 /// XM_217247 | Tmem108 | transmembrane protein 108 | 0.68 |
| 1377390_at | NM_001002802 | Bace2 | beta-site APP-cleaving enzyme 2 | 0.68 |
| 1370813_at | NM_172038 | Gstm5 | glutathione S-transferase, mu 5 | 0.68 |
| 1374943_at | NM_001106547 | RGD1311378 | similar to RIKEN cDNA 2010011I20 | 0.68 |
| 1373337_at | NM_001113754 | Grhpr | glyoxylate reductase/hydroxypyruvate reductase | 0.68 |
| 1370218_at | NM_012595 | Ldhb | lactate dehydrogenase B | 0.68 |
| 1379693_at | NM_032106 | Robo2 | roundabout, axon guidance receptor, homolog 2 (Drosophila) | 0.68 |
| 1374925_at | NM_001134874 | Nab2 | Ngfi-A binding protein 2 | 0.68 |
| 1369106_at | NM_057098 | Tcea2 | transcription elongation factor A (SII), 2 | 0.68 |
| 1373584_at | NM_001109056 | RGD1559643 | similar to hypothetical protein A430031N04 | 0.68 |
| 1383653_at | NM_001012012 | Snx11 | sorting nexin 11 | 0.68 |
| 1376610_a_at | XM_001080633 /// XM_222260 | LOC304558 | similar to TPR repeat-containing protein KIAA1043 | 0.68 |
| 1367746_a_at | NM_031830 | Flot2 | flotillin 2 | 0.67 |
| 1374222_at | NM_001004260 | Slc22a18 | solute carrier family 22, member 18 | 0.67 |
| 1370459_at | NM_145093 | Aard | alanine and arginine rich domain containing protein | 0.67 |
| 1382467_at | NM_001037365 /// NM_001077435 | Bex1 /// Bex2 | brain expressed gene 1 /// brain expressed X-linked 2 | 0.67 |
| 1371081_at | XM_001060956 /// XM_002726175 /// XM_002729173 /// XM_215985 | Rapgef4 | Rap guanine nucleotide exchange factor (GEF) 4 | 0.67 |
| 1375183_at | NM_175582 | Id4 | Inhibitor of DNA binding 4 | 0.67 |
| 1381423_at | NM_001005553 | Git2 | G protein-coupled receptor kinase interacting ArfGAP 2 | 0.67 |
| 1374707_at | NM_001170472 | RGD1309922 | similar to 2610301G19Rik protein | 0.67 |
| 1371091_at | NM_001168633 | Irs2 | insulin receptor substrate 2 | 0.66 |
| 1368202_a_at | NM_024159 | Dab2 | disabled homolog 2 (Drosophila) | 0.66 |
| 1398380_at | NM_001013938 | Vwa1 | von Willebrand factor A domain containing 1 | 0.66 |
| 1373889_at | NM_001168284 /// XM_340957 /// XR_009483 | Igsf7 /// LOC363715 /// RGD1561778 | immunoglobulin superfamily, member 7 /// similar to CLM3 /// similar to dendritic cell-derived immunoglobulin(Ig)-like receptor 1, DIgR1 - mouse | 0.66 |
| 1385637_at | XM_001065678 /// XM_002726537 | Svep1 | Sushi, von Willebrand factor type A, EGF and pentraxin domain containing 1 | 0.66 |
| 1380243_at | NM_001135780 | RGD1304693 | similar to CG14803-PA | 0.66 |
| 1387018_at | NM_053770 | Sorbs2 | sorbin and SH3 domain containing 2 | 0.66 |
| 1379304_at | NM_001098793 | Prkrip1 | Prkr interacting protein 1 (IL11 inducible) | 0.66 |
| 1367796_at | NM_030861 | Mgat1 | mannosyl (alpha-1,3-)-glycoprotein beta-1,2-N-acetylglucosaminyltransferase | 0.66 |
| 1378968_at | NM_001024338 | Bcl2l14 | Bcl2-like 14 (apoptosis facilitator) | 0.66 |
| 1373773_at | NM_178105 | Gpm6a | glycoprotein m6a | 0.66 |
| 1369972_at | NM_001108338 | Fbxo21 | F-box protein 21 | 0.65 |
| 1369651_at | NM_012673 | Thy1 | Thy-1 cell surface antigen | 0.65 |
| 1373590_at | NM_001011965 | Stom | stomatin | 0.65 |
| 1382621_at | NM_001107941 | Dcaf12l1 | DDB1 and CUL4 associated factor 12-like 1 | 0.65 |
| 1378163_at | NM_001105974 | Grem2 | gremlin 2, cysteine knot superfamily, homolog (Xenopus laevis) | 0.65 |
| 1368079_at | NM_053826 | Pdk1 | pyruvate dehydrogenase kinase, isozyme 1 | 0.65 |
| 1379235_x_at | NM_001105866 | Cdc45l | CDC45 cell division cycle 45-like (S. cerevisiae) | 0.65 |
| 1384056_at | NM_053429 | Fgfr3 | Fibroblast growth factor receptor 3 | 0.65 |
| 1381975_at | XM_002725748 /// XM_002728837 | RGD1311350 | similar to kIAA0367 | 0.65 |
| 1381190_at | NM_001001515 | Lmo7 | LIM domain 7 | 0.64 |
| 1387260_at | NM_053713 | Klf4 | Kruppel-like factor 4 (gut) | 0.64 |
| 1374237_at | NM_001107179 | Lmod1 | leiomodin 1 (smooth muscle) | 0.64 |
| 1376051_at | NM_175757 | Cryl1 | crystallin, lambda 1 | 0.64 |
| 1371988_at | NM_001033656 | Man1a1 | mannosidase, alpha, class 1A, member 1 | 0.64 |
| 1373315_at | NM_012781 | Arnt2 | aryl hydrocarbon receptor nuclear translocator 2 | 0.64 |
| 1395900_at | NM_001126495 /// NM_001194951 | Chtf8 | CTF8, chromosome transmission fidelity factor 8 homolog (S. cerevisiae) | 0.64 |
| 1384280_at | NM_001107762 | Nusap1 | nucleolar and spindle associated protein 1 | 0.64 |
| 1372640_at | NM_001170481 | Pi16 | peptidase inhibitor 16 | 0.64 |
| 1389024_at | NM_001017499 | Scfd2 | sec1 family domain containing 2 | 0.64 |
| 1369391_at | NM_022202 | Grm8 | glutamate receptor, metabotropic 8 | 0.63 |
| 1379195_s_at | NM_001024268 /// NM_030855 | Lig1 | ligase I, DNA, ATP-dependent | 0.63 |
| 1374616_at | NM_001011921 | Pdgfrl | platelet-derived growth factor receptor-like | 0.63 |
| 1369273_a_at | NM_012868 | Npr3 | natriuretic peptide receptor C/guanylate cyclase C (atrionatriuretic peptide receptor C) | 0.63 |
| 1371959_at | XM_001062079 /// XM_345255 | Hist2h2aa3 | histone cluster 2, H2aa3 | 0.63 |
| 1390412_at | NM_133315 | Slc40a1 | solute carrier family 39 (iron-regulated transporter), member 1 | 0.63 |
| 1387401_at | NM_017131 | Casq2 | calsequestrin 2 (cardiac muscle) | 0.63 |
| 1374983_at | NM_001107438 | RGD1561676 | similar to Kelch domain containing 4 | 0.63 |
| 1395347_at | NM_001034933 | Arsa | arylsulfatase A | 0.62 |
| 1371803_at | NM_172335 | Gm2a | GM2 ganglioside activator | 0.62 |
| 1368114_at | NM_053428 | Fgf13 | fibroblast growth factor 13 | 0.62 |
| 1387294_at | NM_054011 | Sh3bp5 | SH3-domain binding protein 5 (BTK-associated) | 0.62 |
| 1373240_at | NM_001037199 | Dhrs3 | dehydrogenase/reductase (SDR family) member 3 | 0.62 |
| 1373108_at | NM_001012072 | Ppp1r3c | protein phosphatase 1, regulatory (inhibitor) subunit 3C | 0.61 |
| 1372483_at | NM_001107123 | Zfp469 | zinc finger protein 469 | 0.61 |
| 1393601_at | NM_001126268 | Tigd5 | tigger transposable element derived 5 | 0.61 |
| 1387669_a_at | NM_001034090 /// NM_012844 | Ephx1 | epoxide hydrolase 1, microsomal | 0.61 |
| 1372907_at | NM_001002253 | Atp6v0e2 | ATPase, H+ transporting V0 subunit e2 | 0.61 |
| 1373897_at | NM_053905 | Lmnb1 | Lamin B1 | 0.60 |
| 1392973_at | NM_138529 | Nav2 | neuron navigator 2 | 0.60 |
| 1373134_at | NM_001134834 | Fahd2a | fumarylacetoacetate hydrolase domain containing 2A | 0.60 |
| 1388792_at | NM_001077640 | Gadd45g | growth arrest and DNA-damage-inducible, gamma | 0.60 |
| 1379732_at | NM_001025638 | Stx11 | syntaxin 11 | 0.60 |
| 1369428_a_at | NM_024394 | Htr3a | 5-hydroxytryptamine (serotonin) receptor 3a | 0.59 |
| 1383692_at | XM_001060034 /// XM_002725332 | Prelid2 | PRELI domain containing 2 | 0.59 |
| 1372440_at | NM_019197 | Serpine2 | serine (or cysteine) peptidase inhibitor, clade E, member 2 | 0.59 |
| 1390415_at | NM_001011930 | Trip13 | thyroid hormone receptor interactor 13 | 0.59 |
| 1390233_at | NM_001107169 | Gli2 | GLI family zinc finger 2 | 0.59 |
| 1385876_at | XM_001077762 /// XM_002725130 | LOC691984 | similar to Glypican-6 precursor | 0.59 |
| 1374743_at | XM_002729506 | Inadl2 | InaD-like 2 (Drosophila) | 0.59 |
| 1371643_at | NM_171992 | Ccnd1 | cyclin D1 | 0.59 |
| 1371074_a_at | NM_017287 | Mcm6 | minichromosome maintenance complex component 6 | 0.59 |
| 1367676_at | NM_017187 | Hmgb2 | high mobility group box 2 | 0.59 |
| 1371021_at | NM_033443 | Arsb | arylsulfatase B | 0.59 |
| 1395026_at | NM_144561 /// NM_144562 | Fmo4 | flavin containing monooxygenase 4 | 0.58 |
| 1374284_at | NM_001024275 | Rassf4 | Ras association (RalGDS/AF-6) domain family member 4 | 0.58 |
| 1374235_at | NM_175578 | Rcan2 | regulator of calcineurin 2 | 0.58 |
| 1368540_at | NM_031807 | Tpbg | trophoblast glycoprotein | 0.58 |
| 1368480_at | NM_001033852 /// NM_053492 | Slc44a1 | solute carrier family 44, member 1 | 0.57 |
| 1389142_at | NM_001047913 | Sqrdl | sulfide quinone reductase-like (yeast) | 0.57 |
| 1389100_at | XM_001076356 /// XM_002727140 /// XM_236659 | Epm2aip1 | EPM2A (laforin) interacting protein 1 | 0.57 |
| 1382452_at | NM_001007712 | Sdpr | serum deprivation response | 0.57 |
| 1395376_at | XM_001070646 /// XM_237570 | Ddx11 | DEAD/H (Asp-Glu-Ala-Asp/His) box polypeptide 11 (CHL1-like helicase homolog, S. cerevisiae) | 0.56 |
| 1376569_at | NM_001007684 | Klf2 | Kruppel-like factor 2 (lung) | 0.56 |
| 1398582_at | NM_001108048 | Rps6ka5 | ribosomal protein S6 kinase, polypeptide 5 | 0.56 |
| 1368008_at | NM_001110137 /// NM_021751 | Prom1 | prominin 1 | 0.56 |
| 1368059_at | NM_053955 | Crym | crystallin, mu | 0.55 |
| 1372980_at | NM_001109227 | Tspan33 | tetraspanin 33 | 0.55 |
| 1383684_at | NM_001107160 | Asf1b | ASF1 anti-silencing function 1 homolog B (S. cerevisiae) | 0.55 |
| 1387325_at | NM_024143 | Slc27a5 | solute carrier family 27 (fatty acid transporter), member 5 | 0.55 |
| 1369083_at | NM_031147 | Cirbp | cold inducible RNA binding protein | 0.55 |
| 1369625_at | NM_012778 | Aqp1 | aquaporin 1 | 0.55 |
| 1388618_at | NM_001012005 | Nid2 | nidogen 2 | 0.55 |
| 1370178_at | NM_053851 | Cacnb2 | calcium channel, voltage-dependent, beta 2 subunit | 0.55 |
| 1392686_at | NM_001107454 | Nkd2 | naked cuticle homolog 2 (Drosophila) | 0.55 |
| 1376039_at | NM_153296 | Aurka | aurora kinase A | 0.55 |
| 1368256_at | NM_053779 | Serpini1 | serine (or cysteine) peptidase inhibitor, clade I, member 1 | 0.55 |
| 1383578_at | NM_001109204 | Rad51 | RAD51 homolog (RecA homolog, E. coli) (S. cerevisiae) | 0.55 |
| 1376170_at | NM_001130548 | Col14a1 | collagen, type XIV, alpha 1 | 0.55 |
| 1374176_at | NM_001025022 | RGD1308059 | similar to DNA segment, Chr 4, Brigham & Womens Genetics 0951 expressed | 0.55 |
| 1370962_at | NM_173114 | Parm1 | prostate androgen-regulated mucin-like protein 1 | 0.55 |
| 1385327_at | XM_001055698 /// XM_001065031 | Pcdhb13 | protocadherin beta 13 | 0.54 |
| 1370346_at | NM_171991 | Ccnb1 | cyclin B1 | 0.54 |
| 1387025_at | NM_019234 | Dync1i1 | dynein cytoplasmic 1 intermediate chain 1 | 0.54 |
| 1388962_at | NM_001105836 | Pcgf2 | polycomb group ring finger 2 | 0.53 |
| 1371131_a_at | NM_001008767 | Txnip | thioredoxin interacting protein | 0.53 |
| 1388602_at | NM_001077642 | Cfd | complement factor D (adipsin) | 0.53 |
| 1380236_at | XM_001061864 /// XM_002727107 | Itga9 | Integrin alpha 9 | 0.53 |
| 1382274_at | NM_001014790 | Rarres1 | retinoic acid receptor responder (tazarotene induced) 1 | 0.53 |
| 1390474_at | NM_001130559 | Nipal2 | NIPA-like domain containing 2 | 0.52 |
| 1377497_at | NM_001009681 | Oasl | 2'-5'-oligoadenylate synthetase-like | 0.52 |
| 1388484_at | NM_001106542 | Ube2c | ubiquitin-conjugating enzyme E2C | 0.52 |
| 1393806_at | NM_001109603 | Mansc1 | MANSC domain containing 1 | 0.52 |
| 1391013_at | NM_022868 | Pcdh8 | Protocadherin 8 | 0.51 |
| 1385215_at | NM_001108657 | Runx1t1 | runt-related transcription factor 1; translocated to, 1 (cyclin D-related) | 0.51 |
| 1383219_at | NM_001013942 | Clip4 | CAP-GLY domain containing linker protein family, member 4 | 0.51 |
| 1373333_at | NM_001024267 | MGC109340 | similar to Microsomal signal peptidase 23 kDa subunit (SPase 22 kDa subunit) (SPC22/23) | 0.51 |
| 1376185_at | NM_001005878 | Kifc1 | kinesin family member C1 | 0.51 |
| 1371731_at | NM_001106929 | RGD1566215 | Similar to Coatomer gamma-2 subunit (Gamma-2 coat protein) (Gamma-2 COP) | 0.50 |
| 1390037_at | NM_001106550 | Nkain4 | Na+/K+ transporting ATPase interacting 4 | 0.50 |
| 1391317_at | NM_001106134 | Ska1 | spindle and kinetochore associated complex subunit 1 | 0.50 |
| 1370125_at | NM_019189 | Hapln1 | hyaluronan and proteoglycan link protein 1 | 0.49 |
| 1393452_at | NM_001107956 | Car9 | carbonic anhydrase 9 | 0.49 |
| 1369928_at | NM_019212 | Acta1 | actin, alpha 1, skeletal muscle | 0.49 |
| 1372186_a_at | NM_022183 | Top2a | topoisomerase (DNA) II alpha | 0.49 |
| 1375247_at | NM_138502 | Mgll | Monoglyceride lipase | 0.48 |
| 1394912_at | NM_001106623 | RGD1311164 | similar to DNA segment, Chr 6, Wayne State University 163, expressed | 0.48 |
| 1387759_s_at | NM_001039549 /// NM_001039691 /// NM_012683 /// NM_057105 /// NM_130407 /// NM_175846 /// NM_201423 /// NM_201424 /// NM_201425 | Ugt1a1 /// Ugt1a2 /// Ugt1a3 /// Ugt1a5 /// Ugt1a6 /// Ugt1a7c /// Ugt1a8 /// Ugt1a9 | UDP glucuronosyltransferase 1 family, polypeptide A1 /// UDP glucuronosyltransferase 1 family, polypeptide A2 /// UDP glycosyltransferase 1 family, polypeptide A3 /// UDP glucuronosyltransferase 1 family, polypeptide A5 /// UDP glucuronosyltransferase 1 family, polypeptide A6 /// UDP glucuronosyltransferase 1 family, polypeptide A7C /// UDP glycosyltransferase 1 family, polypeptide A8 /// UDP glucuronosyltransferase 1 family, polypeptide A9 | 0.48 |
| 1370951_at | NM_138905 | Ppap2b | phosphatidic acid phosphatase type 2B | 0.48 |
| 1372615_at | NM_031582 | Aoc3 | amine oxidase, copper containing 3 (vascular adhesion protein 1) | 0.48 |
| 1368064_a_at | NM_012545 | Ddc | dopa decarboxylase (aromatic L-amino acid decarboxylase) | 0.47 |
| 1376311_at | NM_001106465 | Ntng1 | netrin G1 | 0.47 |
| 1387219_at | NM_012715 | Adm | adrenomedullin | 0.46 |
| 1381533_at | NM_001013222 | Rnd1 | Rho family GTPase 1 | 0.46 |
| 1373148_at | NM_001106306 | Cpxm2 | carboxypeptidase X (M14 family), member 2 | 0.45 |
| 1374449_at | NM_001007648 | Cdca3 | cell division cycle associated 3 | 0.45 |
| 1374794_at | NM_181635 | Kif15 | kinesin family member 15 | 0.45 |
| 1383641_at | NM_012550 | Ednra | endothelin receptor type A | 0.44 |
| 1390159_at | NM_001108009 | Rasgrp3 | RAS guanyl releasing protein 3 (calcium and DAG-regulated) | 0.44 |
| 1386935_at | NM_024388 | Nr4a1 | nuclear receptor subfamily 4, group A, member 1 | 0.44 |
| 1370334_at | NM_172033 | Plekhb1 | pleckstrin homology domain containing, family B (evectins) member 1 | 0.43 |
| 1387961_at | NM_053848 | Opcml | opioid binding protein/cell adhesion molecule-like | 0.43 |
| 1385051_at | XM_001078892 /// XM_227762 | Gbp4 | guanylate binding protein 4 | 0.42 |
| 1384392_at | NM_181087 | Cyp26b1 | cytochrome P450, family 26, subfamily b, polypeptide 1 | 0.42 |
| 1380182_at | XM_001069190 /// XM_214519 | RGD1563437 | Similar to KIAA1217 | 0.41 |
| 1389448_at | NM_001011893 | 41156 | septin 4 | 0.41 |
| 1370019_at | NM_031834 | Sult1a1 | sulfotransferase family, cytosolic, 1A, phenol-preferring, member 1 | 0.41 |
| 1395765_at | XM_001059692 /// XM_341029 | RGD1307396 | similar to RIKEN cDNA 6330406I15 | 0.40 |
| 1387306_a_at | NM_053633 | Egr2 | early growth response 2 | 0.40 |
| 1374986_x_at | XM_001056542 | LOC679475 | hypothetical protein LOC679475 | 0.39 |
| 1387091_at | NM_017226 | Padi2 | peptidyl arginine deiminase, type II | 0.39 |
| 1379281_at | NM_153737 | Sostdc1 | sclerostin domain containing 1 | 0.39 |
| 1367648_at | NM_013122 | Igfbp2 | insulin-like growth factor binding protein 2 | 0.36 |
| 1393632_at | NM_001107221 | C1qtnf7 | C1q and tumor necrosis factor related protein 7 | 0.34 |
| 1370157_at | NM_022707 | Pln | phospholamban | 0.34 |
| 1375043_at | NM_022197 | Fos | FBJ osteosarcoma oncogene | 0.33 |
| 1367896_at | NM_019292 | Car3 | carbonic anhydrase 3 | 0.32 |
| 1390585_at | NM_022257 | Masp1 | mannan-binding lectin serine peptidase 1 | 0.31 |
| 1369144_a_at | NM_031739 | Kcnd3 | potassium voltage-gated channel, Shal-related subfamily, member 3 | 0.31 |
| 1386965_at | NM_012598 | Lpl | lipoprotein lipase | 0.30 |
| 1384112_at | NM_021576 | Nt5e | 5' nucleotidase, ecto | 0.26 |
| 1384707_at | NM_001135855 | Scara5 | scavenger receptor class A, member 5 (putative) | 0.21 |

a Fold change greater than 1.0 represents increases, while less than 1.0 indicates decreases in stretching versus control group.
