# Supplementary material for: Gene Expression Changes under Cyclic Mechanical Stretching in Rat Retinal Glial (Müller) Cells
Source: PLoS One. 2013 May 27;8(5):e63467. doi: 10.1371/journal.pone.0063467 (PMC3664568; doi:10.1371/journal.pone.0063467)
Supplement: Table S3 — Significantly Upregulated Gene Categories after stretching for 1 h. (DOCX) [file pone.0063467.s003.docx]

| Table S3. Significantly Upregulated Gene Categories after stretching for 1 h | | | |
| --- | --- | --- | --- |
| **Gene Ontology ID** | **Gene Categories** | **Genes (n)** | **P-value** |
| **GO:0003674** | **molecular_function** |  |  |
| *GO:0005488* | *binding* |  |  |
| GO:0001871 | pattern binding | 2 | 0.038 |
| GO:0003676 | nucleic acid binding | 11 | 0.029 |
| GO:0005515 | protein binding | 28 | 0.002 |
| *GO:0030528* | *transcription regulator activity* |  |  |
| GO:0003700 | transcription factor activity | 10 | 0.000 |
| GO:0003702 | RNA polymerase II transcription factor activity | 2 | 0.032 |
| GO:0016563 | transcription activator activity | 5 | 0.001 |
| **GO:0005575** | **cellular_component** |  |  |
| *GO:0031974* | *membrane-enclosed lumen* |  |  |
| GO:0043233 | organelle lumen | 9 | 0.007 |
| *GO:0043226* | *organelle* |  |  |
| GO:0043227 | membrane-bounded organelle | 23 | 0.013 |
| *GO:0044421* | *extracellular region part* |  |  |
| GO:0005615 | extracellular space | 4 | 0.036 |
| *GO:0044422* | *organelle part* |  |  |
| GO:0043233 | organelle lumen | 9 | 0.007 |
| *GO:0044464* | *cell part* |  |  |
| GO:0044424 | intracellular part | 27 | 0.050 |
| **GO:0008150** | **biological_process** |  |  |
| *GO:0001906* | *cell killing* |  |  |
| GO:0001909 | leukocyte mediated cytotoxicity | 2 | 0.005 |
| GO:0031341 | regulation of cell killing | 2 | 0.004 |
| GO:0031343 | positive regulation of cell killing | 2 | 0.003 |
| *GO:0002376* | *immune system process* |  |  |
| GO:0002252 | immune effector process | 3 | 0.016 |
| GO:0006955 | immune response | 4 | 0.046 |
| GO:0002682 | regulation of immune system process | 5 | 0.003 |
| GO:0002684 | positive regulation of immune system process | 4 | 0.003 |
| *GO:0008152* | *metabolic process* |  |  |
| GO:0009058 | biosynthetic process | 20 | 0.000 |
| GO:0043170 | macromolecule metabolic process | 26 | 0.000 |
| GO:0044237 | cellular metabolic process | 28 | 0.000 |
| GO:0044238 | primary metabolic process | 26 | 0.002 |
| GO:0009892 | negative regulation of metabolic process | 8 | 0.000 |
| GO:0009893 | positive regulation of metabolic process | 11 | 0.000 |
| GO:0019222 | regulation of metabolic process | 18 | 0.000 |
| *GO:0009987* | *cellular process* |  |  |
| GO:0006928 | cell motion | 6 | 0.004 |
| GO:0007049 | cell cycle | 7 | 0.001 |
| GO:0007154 | cell communication | 18 | 0.017 |
| GO:0008219 | cell death | 13 | 0.000 |
| GO:0008283 | cell proliferation | 14 | 0.000 |
| GO:0016043 | cellular component organization | 11 | 0.015 |
| GO:0044237 | cellular metabolic process | 28 | 0.000 |
| GO:0048468 | cell development | 12 | 0.000 |
| GO:0048469 | cell maturation | 2 | 0.019 |
| GO:0048869 | cellular developmental process | 16 | 0.000 |
| GO:0051716 | cellular response to stimulus | 7 | 0.000 |
| GO:0048522 | positive regulation of cellular process | 19 | 0.000 |
| GO:0048523 | negative regulation of cellular process | 17 | 0.000 |
| GO:0050794 | regulation of cellular process | 29 | 0.000 |
| *GO:0010926* | *anatomical structure formation* |  |  |
| GO:0048646 | anatomical structure formation involved in morphogenesis | 6 | 0.000 |
| *GO:0022414* | *reproductive process* |  |  |
| GO:0007565 | female pregnancy | 2 | 0.026 |
| *GO:0032501* | *multicellular organismal process* |  |  |
| GO:0003008 | system process | 11 | 0.020 |
| GO:0007275 | multicellular organismal development | 19 | 0.000 |
| GO:0030534 | adult behavior | 2 | 0.025 |
| GO:0048771 | tissue remodeling | 3 | 0.007 |
| GO:0051239 | regulation of multicellular organismal process | 10 | 0.000 |
| GO:0051240 | positive regulation of multicellular organismal process | 3 | 0.023 |
| GO:0051241 | negative regulation of multicellular organismal process | 3 | 0.008 |
| *GO:0032502* | *developmental process* |  |  |
| GO:0001503 | ossification | 3 | 0.009 |
| GO:0007275 | multicellular organismal development | 19 | 0.000 |
| GO:0007568 | aging | 3 | 0.007 |
| GO:0009653 | anatomical structure morphogenesis | 12 | 0.000 |
| GO:0009791 | post-embryonic development | 2 | 0.019 |
| GO:0021700 | developmental maturation | 3 | 0.003 |
| GO:0031077 | post-embryonic camera-type eye development | 1 | 0.018 |
| GO:0048532 | anatomical structure arrangement | 1 | 0.015 |
| GO:0048589 | developmental growth | 3 | 0.004 |
| GO:0048646 | anatomical structure formation involved in morphogenesis | 6 | 0.000 |
| GO:0048856 | anatomical structure development | 19 | 0.000 |
| GO:0048869 | cellular developmental process | 16 | 0.000 |
| GO:0050793 | regulation of developmental process | 19 | 0.000 |
| GO:0051093 | negative regulation of developmental process | 11 | 0.000 |
| GO:0051094 | positive regulation of developmental process | 12 | 0.000 |
| *GO:0040007* | *growth* |  |  |
| GO:0048589 | developmental growth | 3 | 0.004 |
| *GO:0040011* | *locomotion* |  |  |
| GO:0048870 | cell motility | 4 | 0.038 |
| *GO:0048511* | *rhythmic process* |  |  |
| GO:0007622 | rhythmic behavior | 2 | 0.002 |
| GO:0007623 | circadian rhythm | 3 | 0.001 |
| *GO:0050896* | *response to stimulus* |  |  |
| GO:0006950 | response to stress | 18 | 0.000 |
| GO:0006955 | immune response | 4 | 0.046 |
| GO:0007610 | behavior | 7 | 0.000 |
| GO:0009605 | response to external stimulus | 14 | 0.000 |
| GO:0009607 | response to biotic stimulus | 7 | 0.000 |
| GO:0009628 | response to abiotic stimulus | 10 | 0.000 |
| GO:0009719 | response to endogenous stimulus | 10 | 0.000 |
| GO:0042221 | response to chemical stimulus | 20 | 0.000 |
| GO:0051716 | cellular response to stimulus | 7 | 0.000 |
| GO:0048583 | regulation of response to stimulus | 4 | 0.022 |
| GO:0048584 | positive regulation of response to stimulus | 4 | 0.003 |
| *GO:0051179* | *localization* |  |  |
| GO:0051674 | localization of cell | 6 | 0.004 |
| GO:0032879 | regulation of localization | 5 | 0.020 |
| *GO:0051704* | *multi-organism process* |  |  |
| GO:0007565 | female pregnancy | 2 | 0.026 |
| GO:0051707 | response to other organism | 4 | 0.005 |
| *GO:0065007* | *biological regulation* |  |  |
| GO:0050789 | regulation of biological process | 29 | 0.000 |
| GO:0065008 | regulation of biological quality | 10 | 0.003 |
| GO:0065009 | regulation of molecular function | 12 | 0.000 |
| *GO:0048518* | *positive regulation of biological process* |  |  |
| GO:0002684 | positive regulation of immune system process | 4 | 0.003 |
| GO:0009893 | positive regulation of metabolic process | 11 | 0.000 |
| GO:0031343 | positive regulation of cell killing | 2 | 0.003 |
| GO:0045768 | positive regulation of anti-apoptosis | 3 | 0.000 |
| GO:0048522 | positive regulation of cellular process | 19 | 0.000 |
| GO:0048584 | positive regulation of response to stimulus | 4 | 0.003 |
| GO:0051094 | positive regulation of developmental process | 12 | 0.000 |
| GO:0051240 | positive regulation of multicellular organismal process | 3 | 0.023 |
| *GO:0048519* | *negative regulation of biological process* |  |  |
| GO:0009892 | negative regulation of metabolic process | 8 | 0.000 |
| GO:0048523 | negative regulation of cellular process | 17 | 0.000 |
| GO:0051093 | negative regulation of developmental process | 11 | 0.000 |
| GO:0051241 | negative regulation of multicellular organismal process | 3 | 0.008 |
| *GO:0050789* | *regulation of biological process* |  |  |
| GO:0002682 | regulation of immune system process | 5 | 0.003 |
| GO:0019222 | regulation of metabolic process | 18 | 0.000 |
| GO:0031341 | regulation of cell killing | 2 | 0.004 |
| GO:0032879 | regulation of localization | 5 | 0.020 |
| GO:0042752 | regulation of circadian rhythm | 2 | 0.003 |
| GO:0045767 | regulation of anti-apoptosis | 3 | 0.000 |
| GO:0048518 | positive regulation of biological process | 21 | 0.000 |
| GO:0048519 | negative regulation of biological process | 17 | 0.000 |
| GO:0048583 | regulation of response to stimulus | 4 | 0.022 |
| GO:0050793 | regulation of developmental process | 19 | 0.000 |
| GO:0050794 | regulation of cellular process | 29 | 0.000 |
| GO:0051239 | regulation of multicellular organismal process | 10 | 0.000 |
