# Supplementary material for: Gene Expression Changes under Cyclic Mechanical Stretching in Rat Retinal Glial (Müller) Cells
Source: PLoS One. 2013 May 27;8(5):e63467. doi: 10.1371/journal.pone.0063467 (PMC3664568; doi:10.1371/journal.pone.0063467)
Supplement: Table S4 — Significantly downregulated Gene Categories after stretching for 24 h. (DOCX) [file pone.0063467.s004.docx]

Table S4. Significantly downregulated Gene Categories after stretching for 24 h

| **Gene Ontology ID** | **Gene Categories** | **Genes (n)** | **P-value** |
| --- | --- | --- | --- |
| **GO:0003674** | **molecular_function** |  |  |
| *GO:0003824* | *catalytic activity* |  |  |
| GO:0016829 | lyase activity | 3 | 0.005 |
| *GO:0005488* | *binding* |  |  |
| GO:0001871 | pattern binding | 2 | 0.038 |
| **GO:0005575** | **cellular_component** |  |  |
| *GO:0005576* | *extracellular region* |  |  |
| GO:0044421 | extracellular region part | 8 | 0.000 |
| *GO:0044421* | *extracellular region part* |  |  |
| GO:0005615 | extracellular space | 7 | 0.000 |
| **GO:0008150** | **biological_process** |  |  |
| *GO:0000003* | *reproduction* |  |  |
| GO:0022414 | reproductive process | 7 | 0.001 |
| *GO:0008152* | *metabolic process* |  |  |
| GO:0006807 | nitrogen compound metabolic process | 5 | 0.004 |
| GO:0042445 | hormone metabolic process | 2 | 0.039 |
| *GO:0009987* | *cellular process* |  |  |
| GO:0007059 | chromosome segregation | 2 | 0.010 |
| GO:0016043 | cellular component organization | 13 | 0.002 |
| GO:0019725 | cellular homeostasis | 5 | 0.007 |
| GO:0048468 | cell development | 6 | 0.031 |
| GO:0048469 | cell maturation | 2 | 0.019 |
| GO:0048610 | reproductive cellular process | 1 | 0.043 |
| GO:0048869 | cellular developmental process | 10 | 0.007 |
| GO:0051301 | cell division | 3 | 0.010 |
| *GO:0010926* | *anatomical structure formation* |  |  |
| GO:0048646 | anatomical structure formation involved in morphogenesis | 4 | 0.012 |
| *GO:0016032* | *viral reproduction* |  |  |
| GO:0048524 | positive regulation of viral reproduction | 1 | 0.023 |
| GO:0050792 | regulation of viral reproduction | 1 | 0.043 |
| *GO:0022414* | *reproductive process* |  |  |
| GO:0007565 | female pregnancy | 4 | 0.000 |
| GO:0048610 | reproductive cellular process | 1 | 0.043 |
| GO:0032501 | multicellular organismal process |  |  |
| GO:0007275 | multicellular organismal development | 15 | 0.001 |
| *GO:0032502* | *developmental process* |  |  |
| GO:0007275 | multicellular organismal development | 15 | 0.001 |
| GO:0007389 | pattern specification process | 4 | 0.004 |
| GO:0007568 | aging | 3 | 0.007 |
| GO:0009653 | anatomical structure morphogenesis | 9 | 0.004 |
| GO:0009790 | embryonic development | 5 | 0.017 |
| GO:0021700 | developmental maturation | 2 | 0.031 |
| GO:0048532 | anatomical structure arrangement | 1 | 0.015 |
| GO:0048646 | anatomical structure formation involved in morphogenesis | 4 | 0.012 |
| GO:0048856 | anatomical structure development | 14 | 0.001 |
| GO:0048869 | cellular developmental process | 10 | 0.007 |
| *GO:0050896* | *response to stimulus* |  |  |
| GO:0006950 | response to stress | 11 | 0.001 |
| GO:0009605 | response to external stimulus | 7 | 0.006 |
| GO:0009628 | response to abiotic stimulus | 5 | 0.004 |
| GO:0009719 | response to endogenous stimulus | 7 | 0.001 |
| GO:0042221 | response to chemical stimulus | 12 | 0.033 |
| *GO:0051179* | *localization* |  |  |
| GO:0051235 | maintenance of location | 2 | 0.015 |
| *GO:0051704* | *multi-organism process* |  |  |
| GO:0007565 | female pregnancy | 4 | 0.000 |
| *GO:0065007* | *biological regulation* |  |  |
| GO:0065008 | regulation of biological quality | 10 | 0.003 |
| *GO:0048518* | *positive regulation of biological process* |  |  |
| GO:0010884 | positive regulation of lipid storage | 1 | 0.020 |
| GO:0048524 | positive regulation of viral reproduction | 1 | 0.023 |
| *GO:0050789* | *regulation of biological process* |  |  |
| GO:0050792 | regulation of viral reproduction | 1 | 0.043 |
